# Distribution patterns of small-molecule ligands in the protein universe and implications for origin of life and drug discovery

**DOI:** 10.1186/gb-2007-8-8-r176

**Published:** 2007-08-29

**Authors:** Hong-Fang Ji, De-Xin Kong, Liang Shen, Ling-Ling Chen, Bin-Guang Ma, Hong-Yu Zhang

**Affiliations:** 1 Shandong Provincial Research Center for Bioinformatic Engineering and Technique, Center for Advanced Study, Shandong University of Technology, Zibo 255049, PR China

## Abstract

**Background:**

Extant life depends greatly on the binding of small molecules (such as ligands) with macromolecules (such as proteins), and one ligand can bind multiple proteins. However, little is known about the global patterns of ligand-protein mapping.

**Results:**

By examining 2,186 well-defined small-molecule ligands and thousands of protein domains derived from a database of druggable binding sites, we show that a few ligands bind tens of protein domains or folds, whereas most ligands bind only one, which indicates that ligand-protein mapping follows a power law. Through assigning the protein-binding orders (early or late) for bio-ligands, we demonstrate that the preferential attachment principle still holds for the power-law relation between ligands and proteins. We also found that polar molecular surface area, H-bond acceptor counts, H-bond donor counts and partition coefficient are potential factors to discriminate ligands from ordinary molecules and to differentiate super ligands (shared by three or more folds) from others.

**Conclusion:**

These findings have significant implications for evolution and drug discovery. First, the chronology of ligand-protein binding can be inferred by the power-law feature of ligand-protein mapping. Some nucleotide-containing ligands, such as ATP, ADP, GDP, NAD, FAD, dihydro-nicotinamide-adenine-dinucleotide phosphate (NDP), nicotinamide-adenine-dinucleotide phosphate (NAP), flavin mononucleotide (FMN) and AMP, are found to be the earliest cofactors bound to proteins, agreeing with the current understanding of evolutionary history. Second, the finding that about 30% of ligands are shared by two or more domains will help with drug discovery, such as in finding new functions from old drugs, developing promiscuous drugs and depending more on natural products.

## Abstract 2

Ligand-protein mapping was found to follow a power law and the preferential attachment principle, leading to the identification of the molecules, mostly nucleotide-containing compounds, that are likely to have evolved earliest.

## Background

Life is essentially a molecular network, not only in the individual sense but also at the ecosystem level [1,2]. The network depends greatly on the binding of small molecules (for example, ligands and cofactors) with macromolecules (for example, proteins). Small-molecule ligands not only participate in many basic enzymatic reactions (as coenzymes or substrates) to build metabolic networks, but also act as extra- and intra-cellular signals to help construct regulation networks [3-9]. The great potential of small-molecule ligands to make links between different proteins means that one ligand can bind to diverse targets [10-13]. In fact, some ligands are extremely powerful in contacting proteins, which are termed hubs of biochemical networks [14-17]. However, little is known about the global patterns of ligand-protein mapping, which stimulated our interest to do a comprehensive analysis and explore the biological and chemical bases underlying the mapping patterns. Since ligand-protein binding is one of the most basic biochemical processes, the present study has significant implications for tracing the important events in the origin of life and as well as for understanding the new paradigms in drug discovery.

## Results

### Distribution patterns of ligands in the protein universe

Although considerable efforts have been devoted to constructing ligand databases [18-26], it is still a great challenge to select clearly defined ligands from them. Thanks to the endeavor of Rognan and co-workers, a well-defined ligand database, the Annotated Database of Druggable Binding Sites from the PDB (sc-PDB), was released recently [27]. For this database, the ligands were collected according to the following criteria: only host proteins with high-resolution (<2.5 Å) crystal structures were considered; water molecule, metal ions and other 'unwanted molecules' (for example, solvents, detergents and covalently bound ligands) were removed; only small-molecular-weight ligands (ranging from 70 to 800 Da for heavy atoms) were selected; and only ligands with a limited solvent-exposed surface (that is, less than 50% of their surface exposed to the solvent) were picked. In addition, the corresponding binding sites were also extracted and were defined by all of the protein residues with at least one atom within 6.5 Å of any ligand atom. Taken together, the clear definition for the ligands in sc-PDB guarantees the repeatability of the present analysis, which gives sc-PDB an advantage over other ligand databases.

Through searching sc-PDB, 2,186 small-molecule ligands were selected, which are bound by 5,740 domains (the domains were counted at a non-redundant level and constituted domain space; Additional data file 1). According to SCOP 1.69 [28,29], these domains were classified into 591 folds. As one fold may cover multiple domains and bind more than one ligand, the fold occurrences amounted to 3,224, which constituted the fold universe.

**Figure 1:**
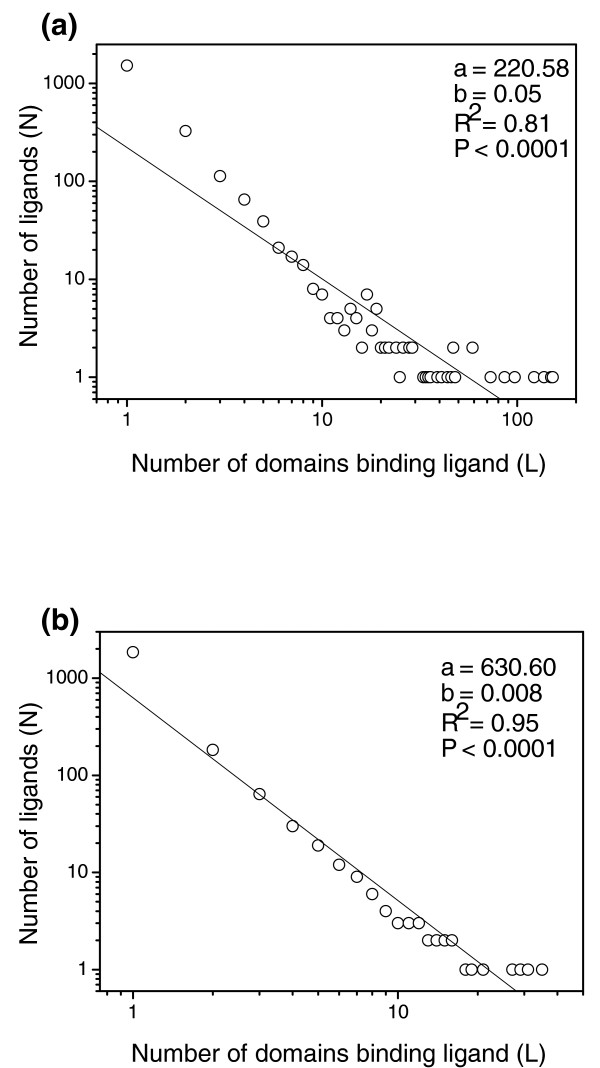
Power-law behaviors of ligand-protein binding. The number of ligands (*N*) decays with an increase in the number (*L*) of **(a) **domains and **(b) **folds that bind the ligand and follows the equation *N *= *aL*^-*b*^. The figure illustrates that a few ligands cover tens of protein domains or folds, while most ligands bind only one domain or fold.

As shown in Additional data file 1, ligands do not distribute evenly in the domain space. A few ligands cover 100+ domains, 681 ligands (31.2%) are shared by 2 or more domains and 1,505 (68.8%) bind only one. Moreover, ligands also populate unevenly in the protein architecture universe. For instance, 1,833 ligands (83.9%) are bound by only one fold, 185 (8.5%) by two, while 24 ligands (1.1%) are bound by 10+ folds (Additional data file 1). The most common ligand, ATP (adenosine-5'-triphosphate), is shared by 35 folds. As illustrated in Figure 1, the number of ligands (*N*) decays with increasing number (*L*) of domains and folds that bind the ligand and follows the power law *N *= *aL*^-*b *^(*P *< 0.0001). It is interesting to note that most of the widely shared ligands (such as those shared by 15+ folds; Additional data file 1) are hubs of metabolic networks [14-16] and are vital to metabolism (especially energy metabolism).

### Biological basis underlying the power-law behaviors of ligand-protein binding

Although power law is a central concept in network sciences and has been implicated in most biological networks [14-16], it is a challenge to elucidate the mechanisms underlying the rule. The most popular theoretical models resort to preferential attachment principle, which attributes the different connections of nodes to their different emerging orders, that is to say, the more connected nodes originated earlier than the less connected nodes [30]. Although the preferential attachment principle has been justified for protein networks [31-33], it remains unclear whether it can be applied to protein-ligand binding.

As a large part of the sc-PDB-derived ligands are synthetic, to explore the applicability of the preferential attachment principle to protein-ligand binding, we extracted bio-ligands from the ligand dataset. To do this, the MetaCyc database (9.5; a metabolic-pathway database that contains 5,253 metabolites) [34] was employed to filter the non-metabolic ligands. As a result, 128 bio-ligands were obtained, which bind to 1,662 domains (counted at a non-redundant level). According to SCOP 1.69 [28,29], these domains were classified into 207 folds. As one fold may cover multiple domains and bind more than one ligand, the fold occurrences amounted to 574. Although these ligands are only metabolism-relevant, they also follow power-law distribution in the protein universe (Additional data file 2).

As the quantity of bio-ligands is limited, to guarantee statistical significance, the 128 bio-ligands were classified into only two categories: first, 70 early ligands, which are owned by both prokaryotic (*Escherichia coli*) and eukaryotic (yeast or higher) species; and second, 54 late ligands, which are owned only by eukaryotic (yeast or higher) species (4 ligands failed in age assignment) (Additional data file 3). It is interesting to note that early ligands cover 7.1 folds on average, in contrast to late ligands, which cover only 1.2 folds on average, and that all (100%) super ligands (shared by 3+ folds) originated early, while most (64.8%) ordinary ligands (bind to 3 or less folds) appeared late. All of these findings strongly suggest that the preferential attachment principle still holds for ligand-protein binding to a large extent.

### Chemical basis underlying the power-law behaviors of ligand-protein binding

It has been widely accepted that protein folds are among the most conserved elements of life [35-37]. However, the present analysis indicates that 353 ligands (16.1%) are shared by 2 or more folds and 104 ligands (4.8%) can cover 3+ folds, which suggests that ligand binding is not constrained by the global architecture of proteins. This finding is consistent with a recent concept that the local structures around an active site are more basic than folds to describe a protein's biological space (binding site for potential ligands) [38]. This phenomenon can be elucidated, at least in part, in terms of the structure-function relationships of proteins. First, binding sites and ligands are quite flexible and plastic [39-41], and therefore, binding-site selection is, to certain extent, ligand dependent [42-44]. Second, ligand binding is governed by a few conserved residues and, thus, is a local rather than a global property of proteins [10,11]. However, the structural factors underlying the strong protein-binding ability of the super ligands still remain unknown. In addition, it is also of interest to explore the structural features discriminating ligands from ordinary molecules. Therefore, the chemical space consisting of ligands and ordinary molecules was charted to reveal the relationship between the ligand distribution patterns in the protein universe and in the chemical space.

The chemical space is composed of 2,176 ligands derived from sc-PDB (due to the lack of atomic parameters, 10 of the 2,186 ligands failed to go through the descriptor calculations) and 2,184 small molecules randomly selected from ACD-SC (Available Chemicals Directory-Screening Compounds, Version 2005.1, Molecular Design Ltd. Information Systems Inc., San Leardo, CA, USA; which collects chemicals that are commercially available and is broadly regarded as a source of ordinary molecules [45]). Seventy descriptors characterizing the structural features of these molecules were calculated, of which 13 were calculated by Sybyl (Tripos Inc., St Louis, Missouri, USA [46]), 49 by Cerius2 (Version 4.10L, Accelrys Inc., San Diego, CA, USA [47]) and 8 by an in-house program written in Perl (Table 1).

**Table 1:** Descriptors of chemical space consisting of sc-PDB-derived ligands and ACD-SC-derived ordinary molecules and corresponding loadings (Varimax normalized) for the first two factors*

Descriptors	Characterization	Factor loadings		Software
			
		1	2	
AREA	Total molecular surface area	**0.974**	0.103	Sybyl
PSA	Polar molecular surface area	0.255	**0.892**	
PV	Polar molecular volume	0.501	0.741	
VOL	Total molecular volume	**0.991**	0.062	
MOLWEIGHT	Molecular weight	**0.958**	0.206	
Acceptor	H-bond acceptor counts	0.464	**0.799**	
Donor	H-bond donor counts	0.376	**0.817**	
BondCount	Total bond counts	**0.972**	0.060	
Chiral	Counts of chiral center	0.367	0.617	
Hydrophobe	Hydrophobic fragment counts	0.767	-0.417	
RingCount	Ring counts	0.686	-0.069	
RotBonds	Number of rotatable bonds	0.630	0.428	
HeavyAtoms	Number of non-H atoms	**0.978**	0.149	

Carbons	Number of carbons atoms	**0.943**	-0.228	Perl
Oxygens	Number of oxygen atoms	0.425	0.793	
Nitrogens	Number of nitrogen atoms	0.475	0.324	
Sulfurs	Number of sulfur atoms	0.141	-0.009	
Phosphorus	Number of phosphorus atoms	0.162	0.617	
Halides	Number of halide atoms	0.076	-0.170	
DoubleBonds	Number of double bonds	0.527	0.378	
TripleBonds	Number of triple bonds	-0.009	-0.109	

RadOfGyration	Radius of gyration	0.888	0.004	Cerius 2
ShadowXY	Surface area projections	**0.967**	0.076	
ShadowXZ		**0.951**	0.053	
ShadowYZ		0.877	0.093	
ShadowXYfrac		-0.610	-0.027	
ShadowXZfrac		-0.421	-0.002	
ShadowYZfrac		-0.289	0.039	
Shadownu		0.268	-0.117	
ShadowXlength		0.849	-0.008	
ShadowYlength		0.798	0.075	
ShadowZlength		0.756	0.059	
Density	Density	-0.089	0.354	
PMImag	Principal moment of inertia	0.819	0.134	
AlogP	Log of the partition coefficient using Ghose and Crippen's method.	0.425	-0.727	
AlogP98	Log of the partition coefficient, atom-type value, using latest parameters.	0.365	**-0.852**	
Fh2o	Desolvation free energy for water	-0.479	-0.762	
Foct	Desolvation free energy for octanol	-0.578	-0.617	
LogP	Log of the partition coefficient.	-0.022	**-0.892**	
MR	Molar refractivity using Hopfinger's method.	0.835	-0.110	
MolRef	Molar refractivity using linear additive method based on AlogP atom types	**0.986**	-0.033	
JX	Balaban indices	-0.567	0.027	
Kappa1	Kappa topological indices	**0.969**	0.189	
Kappa2		**0.926**	0.026	
Kappa3		0.691	0.033	
Kappa1AM		**0.958**	0.220	
Kappa2AM		**0.901**	0.050	
Kappa3AM		0.630	0.046	
PHI	Molecular flexibility index	0.800	0.078	
SC0	Subgraph topological counts	**0.980**	0.147	
SC1		**0.973**	0.125	
SC2		**0.943**	0.186	
SC3P		**0.904**	0.141	
SC3C		0.749	0.389	
SC3CH		0.016	-0.086	
CHI0	Kier and Hall Chi connectivity indices	**0.974**	0.190	
CHI1		**0.983**	0.115	
CHI2		**0.958**	0.210	
CHI3P		**0.939**	0.136	
CHI3C		0.655	0.484	
CHI3CH		0.015	-0.087	
CHIV0		**0.990**	0.076	
CHIV1		**0.971**	0.120	
CHIV2		**0.913**	0.137	
CHIV3P		0.838	0.096	
CHIV3C		0.476	0.148	
CHIV3CH		0.016	-0.088	
Wiener	Wiener topological index	0.854	0.186	
logZ	Logarithm of Hosoya topological index	-0.220	-0.131	
Zagreb	Zagreb topological index	**0.958**	0.162	

*The first factor explains 52.8% of the variance and the second explains 12.7%. Factors with high loadings (>0.9 for first factors and >0.8 for second factors) are shown in bold.

We used factor analysis to visualize the diversity of the molecules. Factor analysis is widely used to study the patterns of relationship among many dependent variables, with the goal of discovering something about the nature of the independent variables (called factors) that affect them [48,49]. In the present analysis, two factors, which can explain 65.5% of the variance, were extracted by principal component analysis and rotated by the Varimax method [50] to chart the two-dimensional chemical space of small molecules. The factor loadings (Varimax normalized) are listed in Table 1.

From the factor loadings, we see that the first factor, explaining 52.8% of the variance, contains high loadings (>0.9; shown in bold in Table 1) from constitutional properties (such as total molecular surface area, total molecular volume, molecular weight, total bond counts, number of non-hydrogen atoms and number of carbons atoms) and topological properties (such as Kappa topological indices, subgraph topological counts, Kier and Hall Chi connectivity indices and Zagreb topological Index). In comparison, the second factor, explaining 12.7% of the variance, contains important contributions (with loadings of higher than 0.8; shown in bold in Table 1) from electronic properties, such as polar molecular surface area, H-bond acceptor counts (whose loading is 0.799), H-bond donor counts and partition coefficient (measured by AlogP98 and LogP).

**Figure 2:**
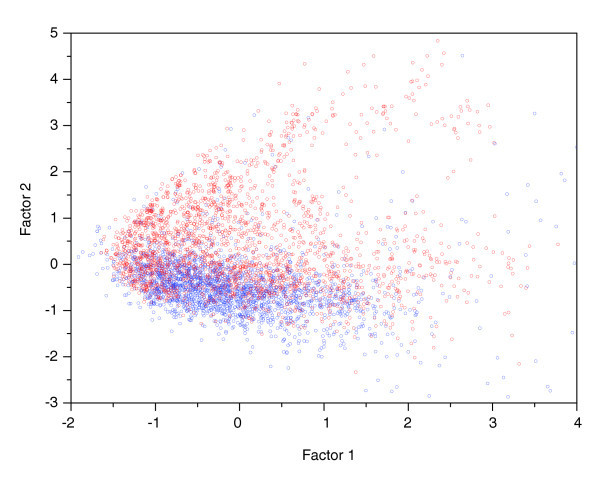
Chemical space consisting of ligands (derived from sc-PDB) and ordinary molecules (randomly selected from ACD-SC), defined by the first two factors derived from 70 descriptors. The figure illustrates that ligands (in red) occupy the relatively upper part of the space, while ordinary molecules (in blue) occupy the relatively lower part, which means that it is the second factor that discriminates ligands from ordinary molecules. From the loadings of the second factor, it can be deduced that polar molecular surface area, H-bond donor counts, H-bond acceptor counts and partition coefficient are likely responsible for the differences between ligands and ordinary molecules, which is supported by the different average values of the four kinds of parameters for ligands and ordinary molecules (Table 2).

**Figure 3:**
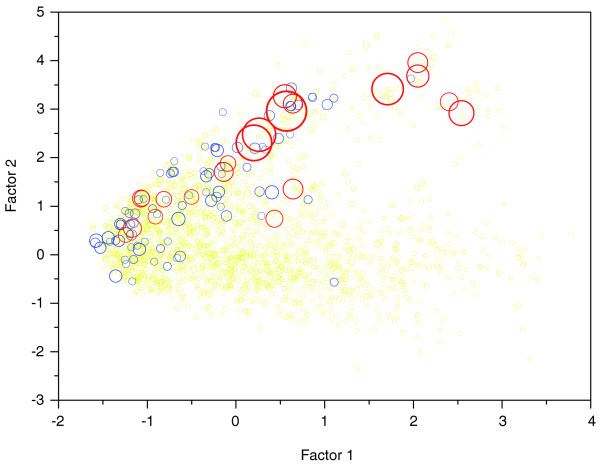
Chemical space consisting of sc-PDB-derived ligands, defined by the first two factors derived from 70 descriptors. The figure illustrates that super ligands (shared by 3+ folds; in blue), especially those that are shared by 10+ folds (in red), concentrate in the relatively upper part of the space (the area of the circle is directly proportional to the number of folds that bind the ligand), which suggests that polar molecular surface area, H-bond donor counts, H-bond acceptor counts and partition coefficient are responsible for the strong protein-binding potential of the super ligands, which is supported by the different average values of the four kinds of parameters for ligands with different protein-binding potentials (Table 2).

In the chemical space formed by the two factors (Figure 2), one can find some differences between the distribution patterns of ligands and ordinary molecules. That is, ligands (in red) occupy the relatively upper part of the space, while ordinary molecules (in blue) hold the relatively lower part, which implies that it is the second factor that discriminates ligands from ordinary molecules. As a consequence, it can be deduced that polar molecular surface area, H-bond donor counts, H-bond acceptor counts and partition coefficient are likely responsible for the differences between ligands and ordinary molecules, which agrees well with the current understanding of the chemical basis of ligand-protein binding that electrostatic interactions (including H-bond) and hydrophobic interactions make major contributions to the binding. More interestingly, as shown in Figure 3, super ligands (in blue and red) do not distribute randomly in the chemical space, but concentrate in the relatively upper part of the space, which suggests that polar molecular surface area, H-bond donor counts, H-bond acceptor counts and partition coefficient are also key factors discriminating super ligands from others.

To shed more light on the above findings, the average values of descriptors characterizing polar molecular surface area, H-bond donors, H-bond acceptors and partition coefficient were calculated for ordinary molecules, ligands and super ligands. From Table 2, it can be seen that there indeed exist correlations between protein-binding ability and the four kinds of parameters. The protein-binding potential of ligands is positively correlated with polar molecular surface area, H-bond donor and acceptor counts, and negatively correlated with partition coefficient (measured by AlogP98 and LogP).

**Table 2:** Average values of descriptors characterizing polar molecular surface area, H-bond donors, H-bond acceptors, partition coefficient and rotatable bonds for ordinary molecules, ligands and ligands with different protein-binding potentials

Descriptor*	Small molecules^†^	Average values	Standard error	Number of molecules
PSA	Molecules	111.81	1.79	2,184
	Ligands	230.59	2.79	2,176
	Ligands (≤ 3)	225.71	2.80	2,072
	Ligands (4-9)	304.28	15.67	80
	Ligands (≥ 10)	406.83	33.10	24
				
Donor	Molecules	1.51	0.04	2,184
	Ligands	3.97	0.07	2,176
	Ligands (≤ 3)	3.87	0.07	2,072
	Ligands (4-9)	5.24	0.43	80
	Ligands (≥ 10)	8.21	0.90	24
				
Acceptor	Molecules	3.35	0.05	2,184
	Ligands	5.87	0.09	2,176
	Ligands (≤ 3)	5.74	0.09	2,072
	Ligands (4-9)	7.69	0.53	80
	Ligands (≥ 10)	11.00	1.18	24
				
AlogP98	Molecules	2.87	0.05	2,184
	Ligands	0.81	0.06	2,176
	Ligands (≤ 3)	0.92	0.06	2,072
	Ligands (4-9)	-1.33	0.25	80
	Ligands (≥ 10)	-1.80	0.38	24
				
LogP	Molecules	0.77	0.08	2,184
	Ligands	-2.27	0.10	2,176
	Ligands (≤ 3)	-2.10	0.10	2,072
	Ligands (4-9)	-5.06	0.50	80
	Ligands (≥ 10)	-8.11	0.96	24
				
RotBond	Molecules	4.88	0.09	2,184
	Ligands	7.49	0.11	2,176
	Ligands (≤ 3)	7.43	0.11	2072
	Ligands (4-9)	8.00	0.50	80
	Ligands (≥ 10)	11.33	1.19	24

* PSA, polar molecular surface area; Donor, H-bond donor counts; Acceptor, H-bond acceptor counts; AlogP98, log of the partition coefficient, atom-type value, using latest parameters; LogP, log of the partition coefficient; RotBond, number of rotatable bonds. ^†^Molecules, ACD-SC-derived ordinary molecules; Ligands, sc-PDB-derived ligands; Ligands (≤ 3), ligands covering ≤ 3 folds; Ligands (4-9), ligands covering 4-9 folds; Ligands (≥ 10), ligands covering ≥ 10 folds.

Recently, through examining the conformational diversity of some very common ligands (that is, ATP, NAD and FAD) bound to proteins, Stockwell and Thornton [41] suggested that molecular flexibility is important for ligands to bind diverse proteins. This opinion is partially supported by the present analysis. Although the contribution from the number of rotatable bonds (RotBonds) to the second factor is not very strong (the loading is 0.428; Table 1), there is a correlation between the protein-binding ability of ligands and index RotBonds. As listed in Table 2, the average RotBonds for ligands is significantly higher than that for ordinary molecules (independent samples *t*-test shows that *P *< 0.0001), and it is clear that the more folds the ligands cover, the higher the average RotBonds are for the ligands.

## Discussion

Since ligand-protein binding is one of the most basic biochemical processes, the present findings have broad biological and medical implications.

### Implications for tracing the chronology of ligand binding to proteins

The most challenging issue in life sciences may be elucidating how organisms originated from inorganic scratches (gases, water and clays), during which one of the most important missions is to establish the chronology of the important biological events. Thanks to the continuing efforts of chemists and biologists, the chronologies of the evolution of amino acids and proteins have been established in principle [37,51-55]. However, as many proteins bind ligands that are essential for their functions and the ligands are likely to have originated independently of proteins [56-59], the binding of ligands with primordial proteins would also be a critical step in the origin of life. Thus, it is intriguing to explore the chronology of ligand-protein binding and answer the following questions: which ligand was first recognized by a protein and what kind of architecture did the host protein have. Nevertheless, since there is no fossil of the last universal common ancestor, let alone the more ancestral organisms, it is a great challenge to trace the protein-binding history of early ligands.

As stated above, through determining the protein-binding ages of ligands, a rough temporal order (early or late) for ligand-protein binding can be inferred (as shown in Additional data file 3). However, considering the fact that fold distribution pattern in the sequence universe helps greatly to reveal the chronology of the evolution of protein architecture [37,53,54], we speculate that the power-law distribution of ligands in the protein universe may implicate a more explicit temporal order for ligand-protein binding. In fact, the preferential attachment principle underlying the power-law behavior of ligand-protein mapping suggests that the more widely a ligand is shared, the earlier it bound to proteins. As protein architecture is more conserved than sequence [35-37], the fold-based inference is believed to be more robust than the domain-based one. Therefore, the nine bio-ligands that are most popular in the fold universe (covering 15+ folds; Table 3) are considered to have bound their host proteins relatively earlier than others and to follow the order (from early to late): ATP, ADP (adenosine-5'-diphosphate), GDP (guanosine-5'-diphosphate), NAD (nicotinamide-adenine-dinucleotide), FAD (flavin-adenine dinucleotide), NDP (dihydro-nicotinamide-adenine-dinucleotide phosphate), NAP (nicotinamide-adenine-dinucleotide phosphate), FMN (flavin mononucleotide) and AMP (adenosine monophosphate).

**Table 3:** The most prevalent bio-ligands in the fold universe (shared by 15+ folds) and the most common folds used by host proteins of each ligand

Ligands	Number of folds	Most common folds
Adenosine-5'-triphosphate (ATP)	35	P-loop containing nucleoside triphosphate hydrolases (c.37)
Adenosine-5'-diphosphate (ADP)	31	P-loop containing nucleoside triphosphate hydrolases (c.37)
Guanosine-5'-diphosphate (GDP)	29	P-loop containing nucleoside triphosphate hydrolases (c.37)
Nicotinamide-adenine-dinucleotide (NAD)	27	NAD(P)-binding Rossmann-fold domains (c.2)
Flavin-adenine dinucleotide (FAD)	21	FAD/NAD(P)-binding domain (c.3)
Dihydro-nicotinamide-adenine-dinucleotide phosphate (NDP)	18	NAD(P)-binding Rossmann-fold domains (c.2)
Nicotinamide-adenine-dinucleotide phosphate (NAP)	16	NAD(P)-binding Rossmann-fold domains (c.2)
Flavin mononucleotide (FMN)	16	Flavodoxin-like (c.23)
Adenosine monophosphate (AMP)	15	Adenine nucleotide alpha hydrolase-like (c.26)

A close inspection of ATP's host proteins reveals that although ATP covers 35 folds and 97 domains, most domains belong to a small group of folds, indicating that power law is still effective (Additional data file 4). According to the preferential attachment principle of fold usage [37], it is reasonable to infer that the most prevalent fold, P-loop hydrolase (c.37), was employed by ATP's first host (Table 3). Interestingly, c.37 is the most ancient fold predicted by a phylogenomic analysis of protein architectures [37,53,54]. Similar analyses allowed us to deduce the most ancestral host proteins of the other eight early ligands (Additional data file 4, Table 3). It is interesting to note that the predicted earliest hosts for the nine bio-ligands appeared in roughly the same order as the protein structures deduced by a phylogenomic analysis (that is, c.37 is the earliest, followed by c.2, c.23, c.3 and c.26, all of which belong to the α/β class) [37,53,54]. Although no consensus has been reached on the exact temporal order of protein architectures, α/β is generally considered to be the most ancient protein class [37,53,54,60-62]. In addition, based on an extensive analysis of sequences and structures of numerous proteins, Trifonov and co-workers [63-65] also inferred that some P-loop ATP-binding domains represent the most ancient proteins. Recently, through a phylogenomic analysis on protein architectures of modern metabolic networks, Caetano-Anollés and co-workers [66] indicated that enzymes with the P-loop hydrolase fold engaged in nucleotide (especially purine) metabolism may be the most primitive members of metabolic systems. Through examining the structures and functions of these members, we found that most (approximately 80%) of them need ATP to work normally. Therefore, the present speculations on the chronology of ligand-protein binding are self-consistent and are in line with the up-to-date knowledge on protein evolutionary history.

To get a deeper insight into the evolutionary features of ligands, the building block usage of 128 bio-ligands was analyzed. As shown in Additional data file 5, nucleic acid bases are the most frequently used building blocks, followed by carbohydrates and amino acids, which is in accordance with Nobeli *et al*.'s [67] finding that nucleic acid bases are the most common fragments of metabolites. More interestingly, many early bio-ligands (45.0%) contain nucleic acid bases; in particular, the nine earliest bio-ligands all contain one or more bases. In contrast, carbohydrates or amino acids are contained by only a small proportion of early bio-ligands (25.0% and 7.5%, respectively). This provides further evidence to support the notion that early ligands are vestiges of the RNA world [56].

As mentioned above, the presently revealed chronology of early ligands' host proteins is roughly in line with the previously deduced evolutionary history of protein architectures [37,53,54]. Thus, it is interesting to ask: is the accordance between both events fortuitous? Our answer is maybe not. Considering the prevalent ligand-induced protein folding [68-72], we conjecture that early ligands might have facilitated protein formation as catalysts (to assemble amino acids or peptide segments), as molecular chaperons (to help protein folding) and/or as selectors (because of the important functions of the early ligands), which naturally resulted in the accordance between both events. This conjecture implicates that the origin of primitive proteins benefited from ligand binding, which is reasonable in terms of the thermodynamics of ligand binding and protein folding.

It has been found that some early ligands, such as ADP and GDP, can bind proteins related to the very old P-loop hydrolase fold (for example, preprotein translocase SecA (1M74), ADP-ribosylation factor-like protein 3 (1FZQ) and GTP-binding protein (1A4R)) with an affinity (free energy) of 10-15 kcal/mol [73], which is just in the range of the free energy loss (10-20 kcal/mol) during protein folding [74,75]. Thus, the free energy release during ligand binding may meet the free energy demand during protein folding. It is tempting to examine the conjecture of ligand-induced formation and/or folding of primordial proteins through experimentation. To do that, *in vitro *selection may be an appropriate methodology [76]. It is interesting to note that *in vitro *selection of proteins (consisting of 80 residues) targeted to bind ATP has been performed [77]. The randomly generated proteins indeed belong to the α/β class, but are not related to P-loop hydrolases fold [78]. However, considering the fact that the shortest protein sequence for the P-loop hydrolase fold contains 94 residues (according to the Protein Databank), we suggest that to explore whether the formation of the most ancient proteins was induced by ATP, one should adopt longer protein sequences in the *in vitro *selection experiments and use small amino acids as building blocks, because in the primordial world only these amino acids were available [51,55].

### Implications for understanding the new paradigms in drug discovery

Nowadays, the pharmaceutical industry is facing an unprecedented challenge. Global research funding has doubled since 1991, whereas the number of approved new drugs has fallen by 50% [79,80]. To meet the more-investment-less-outcome challenge, some novel drug discovery strategies have appeared in recent years, which include finding new functions from old drugs, developing promiscuous drugs rather than selective agents and depending more on natural products than on combinatorial libraries of synthetic compounds to derive drug leads. Since the essence of drug action is the binding between drugs and target biomolecules (most of which are proteins), the ligand-protein binding features revealed in the present study have important implications for understanding these new drug discovery strategies.

As indicated above, approximately 30% of ligands are bound by two or more domains (this number gets ~15%, if counted on fold level), which suggests that if a ligand can bind to a protein, it has great potential to bind to others. Considering the fact that the US Food and Drug Administration (FDA) has approved approximately 2,000 drugs (chemical entities) and there exist only 2,000-3,000 druggable genes and 600-1,500 drug targets [81,82], it is truly possible to find new functions from these old 'safe' drugs, which supports an increasingly shared notion in drug development that the most fruitful basis for the discovery of a new drug is to start with an old drug [83-85].

Since most human diseases, such as cancer, diabetes, heart disease, arthritis and neurodegenerative diseases, involve multiple pathogenetic factors, the more-investment-less-outcome predicament is attributed in part to the limitations of the current one-drug-one-target paradigm in drug discovery [79,86]. Therefore, more and more efforts are devoted to finding new therapeutics aimed at multiple targets [86], which is becoming a new paradigm in drug discovery. To hit the multiple targets implicated in complex diseases, two strategies are conceivable. One is called the multicomponent therapeutic strategy, which incorporates two or more active ingredients in one drug [86-89], as was applied in some traditional medicines (in China and many other countries) and in recently developed drug cocktails. The other is to hit the multiple targets with a single component, which is termed the one-ligand-multiple-targets strategy or promiscuous drug strategy [89-99]. Compared with the former strategy, the latter might take advantage of lower risks of drug-drug interactions and more predictable pharmacokinetic behaviors [91,92] and thus has been paid more and more attention. The feasibility of the one-ligand-multiple-targets strategy is supported by the present findings, because a certain proportion of ligands do indeed bind to two or more domains (even folds). In addition, the presently revealed structural features of super ligands are of significance for selecting and/or designing multipotent agents. Of course, the new strategy should be treated with wariness, because of the potential side effects of the promiscuous ligands.

Another feature of the recent drug discovery paradigm shift is that more attention has been given to natural-product repositories rather than combinatorial libraries of synthetic compounds for finding novel drug leads [100,101]. Due to their biosynthetic origin, natural products are natively bound to proteins (synthases). In light of the present findings, one can conclude that natural products have more potential than synthetic compounds to bind proteins, including those of human, which helps to understand the natural product-based drug discovery strategy. In addition, it can be inferred that it is rather easy to build a protein-ligand network on the basis of naturally occurring small-molecule ligands, which definitely benefits the birth of networked life and facilitates the formation of links within different species.

## Materials and methods

### Data selection/collection

Until June 2006, 2,721 ligands had been recorded in sc-PDB. As our interest was focused on non-peptide ligands, 433 peptides were eliminated. After removing 102 repeated ligands (which have the same structures to others but were given different release names), 2,186 small-molecule ligands remained (Additional data file 1), which bind to 5,740 non-redundant domains (to remove the redundancy of domains, only one domain was chosen from each species). Domain is defined as an independently folded unit within a protein, often joined by a flexible segment of the polypeptide chain [102]. For a small proportion of ligands that are shared by two domains, both domains were counted. According to SCOP 1.69 [28,29], these domains were classified into 591 folds. As one fold may cover multiple domains and hold more than one ligand, the fold occurrences amounted to 3,224.

Since sc-PDB is a subset of the PDB, one may be concerned about the robustness of the conclusions derived when using it. However, considering the facts that the present inferences were made mainly on the level of protein fold and that folds are much more conserved than domains, and thus fold increase is much slower than that of domains in the PDB [103], it is believed that the present conclusions are solid. In fact, even if the latest data of the sc-PDB (containing 396 new ligands and 827 new domains, which were kindly provided by Dr Rognan and have not been uploaded on the website) are considered, all of the present conclusions still hold.

### Descriptor calculation

Seventy descriptors characterizing the structural features of 2,186 ligands selected from the sc-PDB and 2,184 small molecules randomly selected from ACD-SC were calculated by Sybyl (13 descriptors) [46], Cerius 2 (49 descriptors) [47] and an in-house program written in Perl (8 descriptors). Then, the calculated data were linked together with Perl for further analysis. Because of the lack of atomic parameters for ten ligands (that is, 2,3,4,5,6-pentafluorobenzyl alcohol, 2-amino-4-oxo-4,7-dihydro-3h-pyrrolo [2,3-d] pyrimidine-5-carbonitrile, 3,5,3',5'-tetraiodo-l-thyronine, 6,7-dinitroquinoxaline-2,3-dione, 9-hydroxy aristolochic acid, 3,5,7-trihydroxy-2-(4-hydroxyphenyl)-4h-chromen-4-one, 5-hydroxy-2-(4-hydroxyphenyl)-1-benzofuran-7-carbonitrile, 3,3',5,5'-tetraiodothyroacetic acid, 3,5,7,3',4'-pentahydroxyflavone and radicicol), some descriptors could not be calculated for these molecules. Hence, only 2,176 ligands went through the calculation. However, as each of the ten ligands covers only one fold, their absence has no impact on the conclusion of the present study.

### Factor analysis

SPSS 13.0 (SPSS Inc., Chicago, IL, USA) was employed to do the factor analysis. The factors were extracted by means of principal component analysis [48,49] and the parameter settings were as follows: a correlation matrix was used; and two factors were extracted to visualize the two-dimensional chemical space of ligands and ordinary molecules. In order to simplify the interpretation of the extracted factors, factor rotation was performed, during which the most popular orthogonal rotation method, Varimax, developed by Kaiser [50], was employed. For other variables, default parameters were adopted.

### Age assignment for bio-ligands

An early bio-ligand is defined as that owned by both prokaryotic (*E. coli*) and eukaryotic (yeast or higher) species, while a late bio-ligand is defined as that owned only by eukaryotic (yeast or higher) species. As there is no direct information on ligand ownership, we used the information of their host proteins to deduce their ages. That is, a ligand is early, provided that at least one of its host proteins is owned by both *E. coli *and yeast (or higher species); and a ligand is late if none of its host proteins is owned by *E. coli *but at least one is owned by yeast or higher species. During the age-assigning process, not only the host proteins recorded in sc-PDB were checked, but also the corresponding homologous proteins retrieved from Swiss-Prot [104] were considered.

## Abbreviations

ACD-SC, Available Chemicals Directory-Screening Compounds; RotBond, rotatable bond; sc-PDB, Annotated Database of Druggable Binding Sites from the PDB.

## Authors' contributions

H.-Y.Z. designed the study. H.-F.J., D.-X.K. and L.S. collected the data and performed the calculation. All authors analyzed the data. H.-Y.Z., H.-F.J. and L.-L.C. wrote the paper.

## Additional data files

The following additional data are available with the online version of this paper. Additional data file 1 lists ligands and the numbers of domains and folds that bind them. Additional data file 2 illustrates the power-law behaviors of metabolism-relevant ligands. Additional data file 3 provides building blocks and ownerships of metabolism-relevant ligands. Additional data file 4 illustrates the power-law behaviors of folds for proteins binding ATP, ADP and NAD. Additional data file 5 illustrates the building block usage of bio-ligands.

## Supplementary Material

Additional data file 1 Ligands and the numbers of domains and folds that bind them. Click here for file

Additional data file 2 Power-law behaviors of metabolism-relevant ligands. Click here for file

Additional data file 3 Building blocks and ownerships of metabolism-relevant ligands. Click here for file

Additional data file 4 Power-law behaviors of folds for proteins binding ATP, ADP and NAD. Click here for file

Additional data file 5 Building block usage of bio-ligands. Click here for file
