# Supplementary material for: Distribution patterns of small-molecule ligands in the protein universe and implications for origin of life and drug discovery
Source: Genome Biol. 2007 Aug 29;8(8):R176. doi: 10.1186/gb-2007-8-8-r176 (PMC2375006; doi:10.1186/gb-2007-8-8-r176)
Supplement: Additional data file 1 — Ligands and the numbers of domains and folds that bind them. [file gb-2007-8-8-r176-S1.doc]

**Additional data file 1**

Ligands and the numbers of domains and folds that bind the ligand.

| **Ligands** | **Full name of ligands** | Number of domains^a^ | Number of folds |
| --- | --- | --- | --- |
| 001 | 1-[2,2-difluoro-2-(3,4,5-trimethoxy-phenyl)-acetyl]-Piperidine-2-carboxylicAcid4-phenyl-1-(3-pyridin-3-Yl-propyl)-butylEster | 1 | 1 |
| 041 | 2-(3-fluoro-4-hydroxyphenyl)-7-vinyl-1,3-benzoxazol-5-Ol | 1 | 1 |
| 084 | 4-[5-[2-(1-phenyl-ethylamino)-pyrimidin-4-yl]-1-methyl-4-(3-trifluoromethylphenyl)-1h-imidazol-2-yl]-Piperidine | 1 | 1 |
| 094 | 1-(2,6-dichlorophenyl)-6-[(2,4-difluorophenyl)Sulfanyl]-7-(1,2,3,6-tetrahydro-4-pyridinyl)-3,4-Dihydropyrido[3,2-d]pyrimidin-2(1h)-one | 1 | 1 |
| 097 | N4-(2,2-dimethyl-1-methylcarbamoyl-propyl)-2,n1-Dihydroxy-3-isobutyl-succinamide | 1 | 1 |
| 100 | 1-(5-chloroindol-3-yl)-3-hydroxy-3-(2h-tetrazol-5-yl)-Propenone | 1 | 1 |
| 101 | 2'-deoxy-adenosine3'-monophosphate | 1 | 1 |
| 106 | 4-(5-bromo-2-oxo-2h-indol-3-ylazo)-benzenesulfonamide | 1 | 1 |
| 111 | (1n)-4-n-butoxyphenylsulfonyl-(2r)-n-Hydroxycarboxamido-(4s)-methanesulfonylamino-Pyrrolidine | 1 | 1 |
| 114 | Compactin | 1 | 1 |
| 115 | 7-[3-(4-fluoro-phenyl)-1-isopropyl-1h-indol-2-yl]-3,5-Dihydroxy-heptanoicAcid | 1 | 1 |
| 116 | 7-[4-(4-fluoro-phenyl)-5-hydroxymethyl-2,6-diisopropyl-pyridin-3-yl]-3,5-dihydroxy-heptanoicAcid | 1 | 1 |
| 117 | 7-[2-(4-fluoro-phenyl)-5-isopropyl-3-phenyl-4-Phenylcarbamoyl-pyrrol-1-yl]-3,5-dihydroxy-heptanoicAcid | 1 | 1 |
| 120 | 2-(2-oxo-1,2-dihydro-pyridin-3-yl)-1h-benzoimidazole-5-carboxamidine | 1 | 1 |
| 129 | [2(formyl-hydroxy-amino)-ethyl]-phosphonicAcid | 1 | 1 |
| 12H | 12-hydroxydodecanoicAcid | 1 | 1 |
| 134 | 6-fluoro-2-[2-hydroxy-3-(2-methyl-cyclohexyloxy)-Phenyl]-1h-indole-5-carboxamidine | 1 | 1 |
| 135 | N-(4-carbamimidoyl-phenyl)-2-hydroxy-benzamide | 1 | 1 |
| 136 | N-(4-carbamimidoyl-3-choro-phenyl)-2-hydroxy-3-iodo-5-Methyl-benzamide | 1 | 1 |
| 138 | N-[5'-o-phosphono-ribofuranosyl]-2-[2-hydroxy-2-[4-[glutamicAcid]-n-carbonylphenyl]-3-[2-amino-4-hydroxy-quinazolin-6-yl]-propanylamino]-acetamide | 1 | 1 |
| 139 | Adenosine-5'-[trihydrogenDiphosphate]P'-3'-esterWith2'-deoxyuridine | 1 | 1 |
| 140 | N-palmitoylglycine | 1 | 1 |
| 145 | 1-o-[o-nitrophenyl]-beta-d-galactopyranose | 1 | 1 |
| 147 | 1-o-[p-nitrophenyl]-beta-d-galactopyranose | 1 | 1 |
| 149 | D-galctopyranosyl-1-on | 1 | 1 |
| 14A | N-Allyl-5-Amidinoaminooxy-Propyloxy-3-Chloro-NCyclopentylbenzamide | 1 | 1 |
| 14W | N-(3-(aminomethyl)benzyl)acetamidine | 1 | 1 |
| 150 | 4,5-dimethyl-1,2-phenylenediamine | 1 | 1 |
| 153 | (2s)-2-[(2,4-dichloro-benzoyl)-(3-trifluoromethyl-Benzyl)-amino]-3-phenyl-propionicAcid | 1 | 1 |
| 158 | 3-(2-mercapto-acetylamino)-4-oxo-pentanoicAcid | 1 | 1 |
| 160 | 3-(3-{2-[(5-methanesulfonyl-thiophene-2-carbonyl)-Amino]-ethyldisulfanylmethyl}-Benzenesulfonylamino)-4-oxo-pentanoicAcid | 1 | 1 |
| 161 | 5-[4-(1-carboxymethyl-2-oxo-propylcarbamoyl)-Benzylsulfamoyl]-2-hydroxy-benzoicAcid | 1 | 1 |
| 162 | (2-{2-[(5-carbamimidoyl-1-methyl-1h-pyrrol-3-ylmethyl)-carbamoyl]-pyrrol-1-yl}-1-cyclohexylmethyl-2-oxo-Ethylamino)-aceticAcid | 1 | 1 |
| 163 | (2-{2-[(5-carbamimidoyl-1-methyl-1h-pyrrol-2-ylmethyl)-carbamoyl]-pyrrol-1-yl}-1-cyclohexylmethyl-2-oxo-Ethylamino)-aceticAcid | 1 | 1 |
| 164 | 2-(3-carboxypropionyl)-6-hydroxy-cyclohexa-2,4-dieneCarboxylicAcid | 1 | 1 |
| 165 | N-(benzylsulfonyl)-3-cyclohexylalanyl-n-(2-amino-1,3-Benzothiazol-6-yl)prolinamide | 1 | 1 |
| 168 | Pantothenyl-aminoethanol-acetatePivalicAcid | 1 | 1 |
| 16D | Hexane-1,6-diamine | 1 | 1 |
| 170 | (2-[6-chloro-3-{[2,2-difluoro-2-(1-oxidopyridin-2-yl)Ethyl]amino}-2-oxopyrazin-1(2h)-yl]-n-[5-chloro-2-(1h-Tetrazol-1-yl)benzyl]acetamide | 1 | 1 |
| 174 | 4-chloro-benzoicAcid | 1 | 1 |
| 186 | [1-(1-Methyl-4,5-Dioxo-Pent-2-Enylcarbamoyl)-2-PhenylEthyl]-Carbamic AcidBenzylEster | 1 | 1 |
| 189 | N-Benzyl-2-(2,6-Dimethylphenoxy)-N-[((3r,4s)-4-{[Isobutyl(Phenylsulfon Yl)Amino]Methyl}Pyrrolidin-3-Yl)Methyl]Acetamide | 1 | 1 |
| 190 | N-{(1s)-1-(3-Bromobenzyl)-4-[(4-Bromophenyl)Sulfonyl]-6-Methyl-2-Oxohe Ptyl}-2-(2,6-Dimethylphenoxy)Acetamide | 1 | 1 |
| 191 | PropionylCoenzymeA | 1 | 1 |
| 194 | 4-{2-[(3-Nitrobenzoyl)Amino]Phenoxy}PhthalicAcid | 1 | 1 |
| 195 | 4-{2,4-Bis[(3-Nitrobenzoyl)Amino]Phenoxy}PhthalicAcid | 1 | 1 |
| 1AC | 1-aminocyclopropanecarboxylicAcid | 1 | 1 |
| 1AL | AllantoateIon | 1 | 1 |
| 1AN | 2-fluoroaniline | 1 | 1 |
| 1BH | N-benzyloxycarbonyl-ala-pro-3-amino-4-phenyl-butan-2-ol | 1 | 1 |
| 1C5 | [[o-phosphono-n-acetyl-tyrosinyl]-glutamyl-3[cyclohexylmethyl]alaninyl]-amine | 1 | 1 |
| 1DA | 1-deaza-adenosine | 1 | 1 |
| 1GN | 2-deoxy-2-aminogalactose | 1 | 1 |
| 1IN | 1-[2-hydroxy-4-(2-hydroxy-5-methyl-Cyclopentylcarbamoyl)5-phenyl-pentyl]-4-(3-pyridin-3-Yl-propionyl)-piperazine-2-carboxylicAcidTert-Butylamide | 1 | 1 |
| 1MZ | 1-methylimidazole | 1 | 1 |
| 1P3 | (3r)-1-acetyl-3-methylpiperidine | 1 | 1 |
| 1PM | N-[(1,3-benzodioxol-5-yl)methyl]-1-[2-(1h-imidazol-1-Yl)pyrimidin-4-yl]-4-(methoxycarbonyl)-piperazine-2-Acetamide | 1 | 1 |
| 1PR | PhosphoricAcidMono-[5-({[5-carbamoyl-3-(5-Phosphonooxy-5-deoxy-ribofuranosyl)-3h-imidazol-4-Ylamino]-methyl}-amino)-2,3,4-trihydroxy-pentyl]Ester | 1 | 1 |
| 1PU | 1-(5-oxo-2,3,5,9b-tetrahydro-1h-pyrrolo[2,1-a]isoindol-9-yl)-3-pyridin-2-yl-urea | 1 | 1 |
| 1PY | 2-oxo-3-phenylpropionicAcid | 1 | 1 |
| 1RB | 1-alpha-d-ribofuranosyl-benzimiazole-5'-phosphate | 1 | 1 |
| 1SM | Methyl2-[({[(4,6-dimethylpyrimidin-2-yl)amino]Carbonyl}amino)sulfonyl]benzoate | 1 | 1 |
| 1UN | 2-[2-hydroxy-3-(3-hydroxy-2-methyl-benzoylamino)-4-PhenylSulfanyl-butyl]-decahydro-isoquinoline-3-CarboxylicAcidTert-butylamide | 1 | 1 |
| 207 | 1-amino-6-cyclohex-3-enylmethyloxypurine | 1 | 1 |
| 209 | 8-amino-1,3-dimethyl-3,7-dihydropurine-2,6-dione | 1 | 1 |
| 213 | 4'-((2s)-2-(1h-1,2,3-benzotriazol-1-yl)-3-{4-[difluoro(phosphono)methyl]phenyl}-2-phenylpropyl)-1,1'-biphenyl-3-ylphosphonicAcid | 1 | 1 |
| 214 | 6-[4-((2r)-2-(1h-1,2,3-benzotriazol-1-yl)-3-{4-[difluoro(phosphono)methyl]phenyl}-2-phenylpropyl)Phenyl]-2-methylquinolin-8-ylphosphonicAcid | 1 | 1 |
| 216 | [4-r-(4-alpha,6-beta,7-beta]-hexahydro-5,6-di(hydroxy)-1,3-di(allyl)-4,7-bisphenylmethyl)-2h-1,3-diazepinone | 1 | 1 |
| 239 | 6-[(z)-amino(imino)methyl]-n-[4-(aminomethyl)phenyl]-2-naphthamide | 1 | 1 |
| 244 | [5-hydroxy-2-(4-hydroxyphenyl)-1-benzofuran-7-yl]Acetonitrile | 1 | 1 |
| 24B | (2,4-difluorophenyl)methanol | 1 | 1 |
| 24T | 2-amino-4-methylthiazole | 1 | 1 |
| 256 | Phenyl(sulfo)aceticAcid | 1 | 1 |
| 25T | 2-amino-5-methylthiazole | 1 | 1 |
| 26P | 2-amino-6-oxopimelicAcid | 1 | 1 |
| 270 | 2h-benzoimidazol-2-ylamine | 1 | 1 |
| 288 | 5-Chloro-1h-Indole-2-CarboxylicAcid{[Cyclopentyl-(2 Hydroxy-Ethyl)-Carbamoyl]-Methyl}-Amide | 1 | 1 |
| 292 | N-(3-cyclopropyl-1h-pyrazol-5-yl)-2-(2-naphthyl)Acetamide | 1 | 1 |
| 2A6 | 2-anilino-6-cyclohexylmethoxypurine | 1 | 1 |
| 2AC | 2-amino-p-cresol | 1 | 1 |
| 2AP | 2-aminopyridine | 1 | 1 |
| 2BN | (1r,4s)-2-azabornane | 1 | 1 |
| 2C5 | 2-chloro-5-(3-chloro-phenyl)-6-[(4-cyano-phenyl)-(3-Methyl-3h-imidazol-4-yl)-Methoxymethyl]-Nicotinonitrile | 1 | 1 |
| 2CH | 2-chlorophenol | 1 | 1 |
| 2CM | 2-chloro-6-methyl-aniline | 1 | 1 |
| 2EZ | 2-ethylimidazole | 1 | 1 |
| 2FM | S-(difluoromethyl)homocysteine | 1 | 1 |
| 2HC | (2e)-3-(2-Hydroxyphenyl)AcrylicAcid | 1 | 1 |
| 2IN | N-(benzylsulfonyl)seryl-n~1~-{4-[amino(imino)methyl]Benzyl}glycinamide | 1 | 1 |
| 2IP | D-myo-inositol-1,4-bisphosphate | 1 | 1 |
| 2LP | 2-allylphenol | 1 | 1 |
| 2MA | 2-methyladenosine-5'-monophosphate | 1 | 1 |
| 2MC | Methacrylyl-coenzymeA | 1 | 1 |
| 2MP | 3,4-dimethylphenol | 1 | 1 |
| 2MZ | 2-methylimidazole | 1 | 1 |
| 2NP | L-2-amino-6-methylene-pimelicAcid | 1 | 1 |
| 2OS | 3-n-octanoylsucrose | 1 | 1 |
| 2PC | 3,4-dihydro-2h-pyrrolium-5-carboxylate | 1 | 1 |
| 2PH | [7,8-dihydro-pterin-6-ylMethanyl]-phosphonophosphate | 1 | 1 |
| 2PU | 1-(5-oxo-2,3,5,9b-tetrahydro-1h-pyrrolo[2,1-a]isoindol-9-yl)-3-(5-pyrrolidin-2-yl-1h-pyrazol-3-yl)-urea | 1 | 1 |
| 2SA | 2-[9-(3,4-dihydroxy-5-phosphonooxymethyl-tetrahydro-Furan-2-yl)-9h-purin-6-ylamino]-succinicAcid | 1 | 1 |
| 312 | 2-(5-{5-[amino(iminio)methyl]-1h-benzimidazol-2-yl}-2'-methoxy-6-oxido-1,1'-biphenyl-3-yl)succinate | 1 | 1 |
| 326 | 2-{(e)-[5-hydroxy-3-methyl-1-(2-methyl-4-sulfophenyl)-1h-pyrazol-4-yl]diazenyl}-4-sulfobenzoicAcid | 1 | 1 |
| 32P | 3-{2-[(5-aminopentyl)amino]-2-oxoethoxy}-5-({[1-(4-Fluorophenyl)ethyl]amino}carbonyl)phenylPhenylmethanesulfonate | 1 | 1 |
| 335 | [4-(2-(1h-1,2,3-benzotriazol-1-yl)-3-{4-[difluoro(phosphono)methyl]phenyl}-2-phenylpropyl)Phenyl](difluoro)methylphosphonicAcid | 1 | 1 |
| 336 | 4-{2-[4-(3,10-dibromo-8-chloro-6,11-dihydro-5h-Benzo[5,6]cyclohepta[1,2-b]pyridin-11-yl)piperidin-1-Yl]-2-oxoethyl}piperidine-1-carboxamide | 1 | 1 |
| 33P | {3-[(3-hydroxy-2-methyl-5-phosphonooxymethyl-pyridin-4-ylmethyl)-amino]-2-methyl-propyl}-phosphonicAcid | 1 | 1 |
| 340 | 5-methoxy-1,2-dimethyl-3-(phenoxymethyl)indole-4,7-Dione | 1 | 1 |
| 34A | 3,4-dimethylaniline | 1 | 1 |
| 358 | 1-(2,6-dichlorophenyl)-5-(2,4-difluorophenyl)-7-Piperidin-4-yl-3,4-dihydroquinolin-2(1h)-one | 1 | 1 |
| 397 | 2-(4-hydroxy-phenyl)benzofuran-5-ol | 1 | 1 |
| 39A | 6-amino-3,9-dimethyl-9h-purin-3-ium | 1 | 1 |
| 3AG | 2-[methyl-(4-methyl-pent-3-enyl)-amino]-ethyl-Diphosphate | 1 | 1 |
| 3AP | 3-aminopyridine | 1 | 1 |
| 3AR | N-omega-propyl-l-arginine | 1 | 1 |
| 3BB | 3-Bromobut-3-En-1-Ol | 1 | 1 |
| 3BT | 2-{2-[3-(2-carbamimidoylsulfanyl-ethyl)-phenyl]-ethyl}-isothiourea | 1 | 1 |
| 3CH | 3-chlorophenol | 1 | 1 |
| 3D1 | 5-(6-amino-purin-9-yl)-2-hydroxymethyl-tetrahydro-Furan-3-ol | 1 | 1 |
| 3DE | 3,5-dimethyl-1-phenyl-1h-pyrazole-4-carboxylicAcidEthylEster | 1 | 1 |
| 3FP | (2r)-1-(dimethylamino)-3-{4-[(6-{[2-fluoro-5-(trifluoromethyl)phenyl]amino}pyrimidin-4-yl)amino]Phenoxy}propan-2-ol | 1 | 1 |
| 3HA | 3-hydroxyanthranilicAcid | 1 | 1 |
| 3HB | 3-hydroxybenzoicAcid | 1 | 1 |
| 3HP | 3-hydroxyphenylacetate | 1 | 1 |
| 3IB | 3-indolebutyricAcid | 1 | 1 |
| 3ID | 3h-indole-5,6-diol | 1 | 1 |
| 3IP | 3-(benzyloxy)pyridin-2-amine | 1 | 1 |
| 3MB | 3-methoxybenzamide | 1 | 1 |
| 3MF | 3-o-methylfructoseInLinearForm | 1 | 1 |
| 3MT | 3-methylthiazoliumIon | 1 | 1 |
| 3NH | (3s)-tetrahydrofuran-3-yl(1r,2s)-3-[4-((1r)-2-{[(s)-Amino(hydroxy)methyl]oxy}-2,3-dihydro-1h-inden-1-yl)-2-benzyl-3-oxopyrrolidin-2-yl]-1-benzyl-2-Hydroxypropylcarbamate | 1 | 1 |
| 3NT | 3-Nitrotoluene | 1 | 1 |
| 3OH | 3-hydroxy-propanoicAcid | 1 | 1 |
| 3PC | (3s)-3,4-di-n-hexanoyloxybutyl-1-phosphocholine | 1 | 1 |
| 3SA | AcarboseDerivedTrisaccharide | 1 | 1 |
| 3SL | (2r)-3-sulfolacticAcid | 1 | 1 |
| 418 | 2-{4-[2-(s)-allyloxycarbonylamino-3-{4-[(2-carboxy-Phenyl)-oxalyl-amino]-phenyl}-propionylamino]-butoxy}-6-hydroxy-benzoicAcidMethylEster | 1 | 1 |
| 429 | 2-{4-[2-acetylamino-3-(4-carboxymethoxy-3-hydroxy-Phenyl)-propionylamino]-butoxy}-6-hydroxy-benzoicAcidMethylEster | 1 | 1 |
| 433 | 2-(4-{3-[1-[2-(2-chloro-6-fluoro-phenyl)-ethyl]-3-(2,3-dichloro-phenyl)-ureido]-propyl}-phenoxy)-2-methyl-PropionicAcid | 1 | 1 |
| 43P | 4-(1h-imidazol-4-yl)-3-(5-ethyl-2,4-dihydroxy-phenyl)-1h-pyrazole | 1 | 1 |
| 44B | 1,1,1,3,3,3-hexafluoro-2-{4-[(2,2,2-trifluoroethyl)Amino]phenyl}propan-2-ol | 1 | 1 |
| 460 | 2-[5-(6-methylpyridin-2-yl)-2,3-dihydro-1h-pyrazol-4-Yl]-1,5-naphthyridine | 1 | 1 |
| 485 | [2-amino-3-(4-hydroxy-phenyl)-propionylamino]-(3,4,5-Trihydroxy-6-methyl-tetrahydro-pyran-2-yl)-AceticAcid | 1 | 1 |
| 493 | {4-[2-acetylamino-2-(1-biphenyl-4-ylmethyl-2-oxo-Azepan-3-ylcarbamoyl)-ethyl]-2-phosphono-phenoxy}-AceticAcid | 1 | 1 |
| 497 | 6-[amino(imino)methyl]-n-[(4r)-4-ethyl-1,2,3,4-Tetrahydroisoquinolin-6-yl]-2-naphthamide | 1 | 1 |
| 49A | 4,9-amino-2,4-deoxy-2,3-dehydro-n-acetyl-neuraminicAcid | 1 | 1 |
| 4AN | 6-amino-benzo[de]isoquinoline-1,3-dione | 1 | 1 |
| 4AP | 4-aminopyridine | 1 | 1 |
| 4AX | (r)-4-amino-isoxazolidin-3-one | 1 | 1 |
| 4BT | 2-{2-[4-(2-carbamimidoylsulfanyl-ethyl)-phenyl]-ethyl}-isothiourea | 1 | 1 |
| 4BZ | 4-(hydroxymethyl)benzamidine | 1 | 1 |
| 4DE | 1-(4-methoxyphenyl)-3,5-dimethyl-1h-pyrazole-4-CarboxylicAcidEthylEster | 1 | 1 |
| 4FA | 4-fluorophenethylAlcohol | 1 | 1 |
| 4HA | 4-hydroxybutan-1-aminium | 1 | 1 |
| 4HC | 4-Hydroxy-2h-Chromen-2-One | 1 | 1 |
| 4HM | 4-hydroperoxy-2-methoxy-phenol | 1 | 1 |
| 4HP | 4-hydroxyphenylacetate | 1 | 1 |
| 4MV | 4-methylValericAcid | 1 | 1 |
| 4NP | 4-nitrophenylPhosphate | 1 | 1 |
| 4PN | 4-piperidino-piperidine | 1 | 1 |
| 4SP | O6-cyclohexylmethoxy-2-(4'-sulphamoylanilino)Purine | 1 | 1 |
| 4TB | 4-(2-thienyl)butyricAcid | 1 | 1 |
| 4TP | 4-hydroxy-l-threonine-5-monophosphate | 1 | 1 |
| 4TR | 2-Bromo-4-{[(4-Cyanophenyl)(4h-1,2,4-Triazol-4-Yl)Amino]Methyl}Phenyl Sulfamate | 1 | 1 |
| 4TZ | 4-{[(4-Cyanophenyl)(4h-1,2,4-Triazol-4-Yl)Amino]Methyl}Phenyl Sulfamate | 1 | 1 |
| 515 | 3-({5-[(n-acetyl-3-{4-[(carboxycarbonyl)(2-Carboxyphenyl)amino]-1-naphthyl}-l-alanyl)amino]Pentyl}oxy)-2-naphthoicAcid | 1 | 1 |
| 537 | 2,6-dihydroanthra/1,9-cd/pyrazol-6-one | 1 | 1 |
| 544 | 2-(1-methyl-3-oxo-3-phenyl-propylamino)-3-{4-[2-(5-Methyl-2-phenyl-oxazol-4-yl)-ethoxy]-phenyl}-propionicAcid | 1 | 1 |
| 572 | 4-[2-(hydroxymethyl)pyrimidin-4-yl]-n,n-Dimethylpiperazine-1-sulfonamide | 1 | 1 |
| 580 | 3-(4-fluorophenyl)-2-(6-methylpyridin-2-yl)-5,6-Dihydro-4h-pyrrolo[1,2-b]pyrazole | 1 | 1 |
| 588 | 2-{[2-(2-carbamoyl-vinyl)-4-(2-methanesulfonylamino-2-Pentylcarbamoyl-ethyl)-phenyl]-oxalyl-amino}-benzoicAcid | 1 | 1 |
| 5AN | 3,5-difluoroaniline | 1 | 1 |
| 5AP | 5-(aminomethyl)-6-(2,4-dichlorophenyl)-2-(3,5-Dimethoxyphenyl)pyrimidin-4-amine | 1 | 1 |
| 5AS | 5'-o-(n-ethyl-sulfamoyl)adenosine | 1 | 1 |
| 5BN | 5-[(2-aminoethyl)amino]-6-fluoro-3-(1h-pyrrol-2-yl)Benzo[cd]indol-2(1h)-one | 1 | 1 |
| 5DE | 1-(4-aminophenyl)-3,5-dimethyl-1h-pyrazole-4-CarboxylicAcidEthylEster | 1 | 1 |
| 5FA | Adenosine-5'-Pentaphosphate | 1 | 1 |
| 5HU | 5-hydroxymethyluridine-2'-deoxy-5'-monophosphate | 1 | 1 |
| 5IN | N-(benzylsulfonyl)-l-seryl-n~1~-{4-[amino(imino)Methyl]benzyl}-o-benzyl-l-serinamide | 1 | 1 |
| 5MB | 5-methylbenzimidazole | 1 | 1 |
| 5MD | 5-methyl-2'-deoxypseudouridine | 1 | 1 |
| 5MP | 5-methylpyrrole | 1 | 1 |
| 5NI | 5-nitroindazole | 1 | 1 |
| 5OB | 5-methoxybenzimidazole | 1 | 1 |
| 5PV | 5-phenylvalericAcid | 1 | 1 |
| 5RM | (5r)-5-(4-methoxy-3-propoxyphenyl)-5-methyl-1,3-oxazol-2(5h)-one | 1 | 1 |
| 5UD | 5-fluorouridine | 1 | 1 |
| 5YL | 5-(6-d-ribitylamino-2,4(1h,3h)pyrimidinedione-5-yl)Pentyl-1-phosphonicAcid | 1 | 1 |
| 607 | 2-(2'-amino-5-{5-[amino(iminio)methyl]-1h-benzimidazol-2-yl}-6-oxido-1,1'-biphenyl-3-yl)succinate | 1 | 1 |
| 612 | 6-cyclohexylthio-1-ethoxymethyl-5-isopropyluracil | 1 | 1 |
| 623 | 3,3'-[3,5-difluoro-4-methyl-2,6-pyridylenebis(oxy)]-Bis(benzenecarboximidamide) | 1 | 1 |
| 645 | [1-tert-butyl-3-(2,4-dichlorophenyl)-5-hydroxy-1h-Pyrazol-4-yl][2-chloro-4-(methylsulfonyl)phenyl]Methanone | 1 | 1 |
| 666 | 6-(4-{[2-(3-iodobenzyl)-3-oxocyclohex-1-en-1-yl]amino}Phenyl)-5-methyl-4,5-dihydropyridazin-3(2h)-one | 1 | 1 |
| 667 | 6-oxo-8,9,10,11-tetrahydro-7h-cyclohepta[c][1]Benzopyran-3-o-sulfamate | 1 | 1 |
| 669 | 1-(5-carboxypentyl)-5-[(2,6-dichlorobenzyl)oxy]-1H-Indole-2-carboxylicAcid | 1 | 1 |
| 675 | 6-[(z)-amino(imino)methyl]-n-phenyl-2-naphthamide | 1 | 1 |
| 678 | (3-{5-[amino(iminio)methyl]-1h-indol-2-yl}-5-bromo-4-Oxidophenyl)acetate | 1 | 1 |
| 680 | 6-methylamino-5-nitroisocytosine | 1 | 1 |
| 688 | 2-[amino(imino)methyl]-2-hydroxyphenoxy]-6-[3-(4,5-Dihydro-1h-imidazol-2-yl)phenoxy]pyridine-4-carboxylicAcid | 1 | 1 |
| 693 | 2-(5-{5-[amino(iminio)methyl]-1h-benzimidazol-2-yl}-2'-fluoro-6-oxido-1,1'-biphenyl-3-yl)succinate | 1 | 1 |
| 697 | 5-hydroxy-2-(4-hydroxyphenyl)-1-benzofuran-7-Carbonitrile | 1 | 1 |
| 6CP | 6-cyclohexylmethoxy-2-(3'-chloroanilino)Purine | 1 | 1 |
| 6DE | 1-(2-chlorophenyl)-3,5-dimethyl-1h-pyrazole-4-CarboxylicAcidEthylEster | 1 | 1 |
| 6NI | 6-nitroindazole | 1 | 1 |
| 6NP | 6((S)-3-Benzylpiperazin-1-Yl)-3-(Naphthalen-2-Yl)-4 (Pyridin-4-Yl)Pyrazine | 1 | 1 |
| 711 | 7-[[6-[[1-(1-iminoethyl)piperidin-4-yl]oxy]-2-methyl-Benzimidazol-1-yl]methyl]naphthalene-2-carboximidamid | 1 | 1 |
| 715 | (2r)-4-oxo-4-[3-(trifluoromethyl)-5,6-dihydro[1,2,4]Triazolo[4,3-a]pyrazin-7(8h)-yl]-1-(2,4,5-Trifluorophenyl)butan-2-amine | 1 | 1 |
| 745 | Trans-6-(2-phenylcyclopropyl)-naphthalene-2-Carboxamidine | 1 | 1 |
| 761 | 3-(oxalyl-amino)-naphthalene-2-carboxylicAcid | 1 | 1 |
| 771 | 4-(4-benzyloxy-2-methanesulfonylamino-5-methoxy-Benzylamino)-benzamidine | 1 | 1 |
| 772 | 2-cyanoquinolin-8-ylDihydrogenPhosphate | 1 | 1 |
| 778 | 4-[(5-{[4-(3-chlorophenyl)-3-oxopiperazin-1-yl]methyl}-1h-imidazol-1-yl)methyl]benzonitrile | 1 | 1 |
| 783 | 3-{5-[amino(iminio)methyl]-1h-indol-2-yl}-5-methoxy-1,1'-biphenyl-2-olate | 1 | 1 |
| 787 | (phenyl-phosphono-methyl)-phosphonicAcid | 1 | 1 |
| 790 | 1,2,3,4-tetrahydroquinolin-8-ylDihydrogenPhosphate | 1 | 1 |
| 791 | 2-phenylmalonicAcid | 1 | 1 |
| 794 | 2-[(carboxycarbonyl)(1-naphthyl)amino]benzoicAcid | 1 | 1 |
| 7A8 | (1r)-dimethyl-(4-methyl-cyclohex-3-enyl)-amine | 1 | 1 |
| 7DE | 3,5-dimethyl-1-(3-nitrophenyl)-1h-pyrazole-4-CarboxylicAcidEthylEster | 1 | 1 |
| 7IN | N-(benzylsulfonyl)seryl-n~1~-{4-[(z)-amino(imino)Methyl]benzyl}serinamide | 1 | 1 |
| 7NI | 7-nitroindazole | 1 | 1 |
| 7RA | 7-alpha-d-ribofuranosyl-2-aminopurine-5'-phosphate | 1 | 1 |
| 7RP | 7-alpha-d-ribofuranosyl-purine-5'-phosphate | 1 | 1 |
| 802 | 2-{5-[Amino(Iminio)Methyl]-1h-Benzimidazol-2-Yl}-4Fluorobenzenolate | 1 | 1 |
| 806 | 7-[[2-[[1-(1-Iminoethyl)Piperidin-4-Yl]Oxy]-9h Carbozol-9-Yl]Methyl]Naphthalene-2-Carboximidamid | 1 | 1 |
| 815 | Thieno[3,2-B]Pyridine-2-SulfonicAcid[1-(1-Amino Isoquinolin-7-Ylmethyl)-2-Oxo-Pyrroldin-3-Yl]-Amide | 1 | 1 |
| 818 | L-685,818(18-Hydroxyascomycin) | 1 | 1 |
| 81A | 2-Methanesulfonyl-BenzenesulfonicAcid3-Methyl-5-((1 Amidinoaminooxymethyl-Cyclopropyl)Methyloxy)Phenylester | 1 | 1 |
| 822 | N-Acetyl-N-[1-(1,1'-Biphenyl-4-Ylmethyl)-2-Oxoazepan 3-Yl]-4-[Difluoro(Phosphono)Methyl]Phenylalaninamide | 1 | 1 |
| 843 | N-Acetyl-N-[1-(1,1'-Biphenyl-4-Ylmethyl)-2-Oxoazepan 3-Yl]-O-Phosphonotyrosinamide | 1 | 1 |
| 846 | [4r--(1alpha,5alpha,7beta)]-3-[(Cycloprophylmethyl)Hexahydro-5,6-Dihyd Roxy-2-Oxo-4,7-Bis(Phenylmethyl)-1h-1,3-Diazepin]Methyl-2-Thiazolylben Zamide | 1 | 1 |
| 852 | 2-{4-[2-Acetylamino-2-(1-Biphenyl-4-Ylmethyl-2-Oxo Azepan-3-Ylcarbamoyl)-Ethyl]-2-Carboxy-PhenylMalonicAcid | 1 | 1 |
| 853 | 5-[2-Acetylamino-2-(1-Biphenyl-4-Ylmethyl-2-Oxo Azepan-3-Ylcarbamoyl)-Ethyl]-2-Carboxymethyl-BenzoicAcid | 1 | 1 |
| 876 | N-Acetyl-N-[1-(1,1'-Biphenyl-4-Ylmethyl)-2-Oxoazepan 3-Yl]-3,4-Diphosphonophenylalaninamide | 1 | 1 |
| 878 | 5-Iodo-2-(Oxalyl-Amino)-BenzoicAcid | 1 | 1 |
| 87Y | 7,8-Dihydro-6-Hydroxymethyl-7-Methyl-7-[2Phenylethyl]-Pterin | 1 | 1 |
| 892 | 3-(3,5-Dibromo-4-Hydroxy-Benzoyl)-2-Ethyl-Benzofuran6-SulfonicAcid (4-Sulfamoyl-Phenyl)-Amide | 1 | 1 |
| 8HG | 8-Hydroxy-2'-Deoxyguanosine | 1 | 1 |
| 8IG | 8-Iodo-Guanine | 1 | 1 |
| 8IN | [3-(1-Benzyl-3-Carbamoylmethyl-2-Methyl-1h-Indol-5 Yloxy)-Propyl-]-PhosphonicAcid | 1 | 1 |
| 901 | 2-[(4-{2-acetylamino-2-[4-(1-carboxy-3-methylsulfanyl-Propylcarbamoyl)-butylcarbamoyl]-ethyl}-2-ethyl-Phenyl)-oxalyl-amino]-benzoicAcid | 1 | 1 |
| 903 | 2-{4-[2-acetylamino-2-(1-biphenyl-4-ylmethyl-2-oxo-Azepan-3-ylcarbamoyl)-ethyl]-2-methoxycarbonyl-phenyl}-2-fluoro-malonicAcid | 1 | 1 |
| 905 | (r)-4-[2-(3-amino-benzenesulfonylamino)-1-(3,5-Diethoxy-2-fluorophenyl)-2-oxo-ethylamino]-2-hydroxy-Benzamidine | 1 | 1 |
| 915 | (2r)-2-{[(4-Fluoro-3-Methylphenyl)Sulfonyl]Amino}-N Hydroxy-2-Tetrahydro-2h-Pyran-4-Ylacetamide | 1 | 1 |
| 936 | 5-methoxy-1,2-dimethyl-3-(4-nitrophenoxymethyl)indole-4,7-dione | 1 | 1 |
| 941 | 2-(4-{2-tert-butoxycarbonylamino-2-[4-(3-hydroxy-2-Methoxycarbonyl-phenoxy)-butylcarbamoyl]-ethyl}-Phenoxy)-malonicAcid | 1 | 1 |
| 961 | 3-fluoro-4-[2-hydroxy-2-(5,5,8,8-tetramethyl-5,6,7,8,-Tetrahydro-naphtalen-2-yl)-acetylamino]-benzoicAcid | 1 | 1 |
| 964 | 3-(4-{2-[2-(2-bromo-acetylamino)-ethyldisulfanyl]-Ethylcarbamoyl}-cyclohexylcarbamoyl)-pyrazine-2-CarboxylicAcid | 1 | 1 |
| 965 | (3-{3-[[2-chloro-3-(trifluoromethyl)benzyl](2,2-Diphenylethyl)amino]propoxy}phenyl)aceticAcid | 1 | 1 |
| 968 | 2-[(7-hydroxy-naphthalen-1-yl)-oxalyl-amino]-benzoicAcid | 1 | 1 |
| 972 | 2-{5-[amino(iminio)methyl]-6-chloro-1h-benzimidazol-2-Yl}-6-isobutoxybenzenolate | 1 | 1 |
| 974 | 1-[2-[5-[amino(imino)methyl]-2-hydroxyphenoxy]-6-[3-(4,5-dihydro-1-methyl-1h-imidazol-2-yl)phenoxy]Pyridin-4-yl]piperidine-3-carboxylicAcid | 1 | 1 |
| 984 | Cyclopropyl-{4-[5-(3,4-dichlorophenyl)-2-[(1-methyl)-Piperidin]-4-yl-3-propyl-3h-imidazol-4-yl]-pyrimidin-2-yl}amine | 1 | 1 |
| 989 | 2-{[4-(2-acetylamino-2-pentylcarbamoyl-ethyl)-Naphthalen-1-yl]-oxalyl-amino}-benzoicAcid | 1 | 1 |
| 991 | 2-{5-[amino(iminio)methyl]-6-chloro-1h-indol-2-yl}-6-(cyclopentyloxy)benzenolate | 1 | 1 |
| 9AM | 9-amino-2-deoxy-2,3-dehydro-n-acetyl-neuraminicAcid | 1 | 1 |
| 9AR | 9-hydroxyAristolochicAcid | 1 | 1 |
| 9DI | 9-deazainosine | 1 | 1 |
| 9HX | 9-deazahypoxanthine | 1 | 1 |
| 9MG | 9-methylguanine | 1 | 1 |
| A15 | 3'-3"-dichlorophenol-1,8-3h-benzo[de]isochromen-1-one | 1 | 1 |
| A2P | Adenosine-2'-5'-diphosphate | 1 | 1 |
| A3B | D-vinylglycine | 1 | 1 |
| A3M | 2-amino-3-methyl-1-pyrrolidin-1-yl-butan-1-one | 1 | 1 |
| A3S | Serine-3'-aminoadenosine | 1 | 1 |
| A45 | 3-(5-amino-7-hydroxy-[1,2,3]triazolo[4,5-d]pyrimidin-2-yl)-benzoicAcid | 1 | 1 |
| A4P | 6-(adenosineTetraphosphate-methyl)-7,8-dihydropterin | 1 | 1 |
| A5P | Arabinose-5-phosphate | 1 | 1 |
| A70 | (2s)-2-[(3r)-3-benzyl-4-n-(4-methylpiperazin-1-yl-Carbonyl)2-ketopiperazin-1-yl]-hexanoicAcidAmideOf(2r,4s,5s)-5-amino-6-cyclohexyl-4-hydroxy-2-iso-PropylhexanoylN-(n-amide) | 1 | 1 |
| A76 | N-{1-benzyl-(2r,3r)-2,3-dihydroxy-4-[3-methyl-2-(3-Methyl-3-pyridin-2-ylmethyl-ureido)-butyrylamino]-5-Phenyl-pentyl}-3-methyl-2-(3-methyl-3-pyridin-2-Ylmethyl-ureido)-butyramide | 1 | 1 |
| A78 | N-{1-benzyl-3-hydroxy-4-[3-methyl-2-(3-methyl-3-Pyridin-2-ylmethyl-ureido)-butyrylamino]-5-phenyl-Pentyl}-3-methyl-2-(3-methyl-3-pyridin-2-ylmethyl-Ureido)-butyramide | 1 | 1 |
| A88 | (5r,6r)-2,4-bis-(4-hydroxy-3-methoxybenzyl)-1,5-Dibenzyl-3-oxo-6-hydroxy-1,2,4-triazacycloheptane | 1 | 1 |
| A8B | N-4'-Quinolyl-N'-9"-(1",2",3",4"-Tetrahydro Acridinyl)-1,8-DiaminooctaneDihydrochloride | 1 | 1 |
| A8N | N-9-(1',2',3',4'-Tetrahydroacridinyl)-1,8Diaminooctane | 1 | 1 |
| AA2 | 4-aryl-2-phenylaminoPyrimidine | 1 | 1 |
| AAE | AcetoaceticAcid | 1 | 1 |
| AAG | N-alpha-l-acetyl-arginine | 1 | 1 |
| AAM | Alpha-adenosineMonophosphate | 1 | 1 |
| AAP | Alpha-(2,6-dichlorophenyl)-alpha-(2-acetyl-5-Methylanilino)acetamide | 1 | 1 |
| AAT | S-adenosyl-1,8-diamino-3-thiooctane | 1 | 1 |
| AAY | 8-[2-((2s)-4-hydroxy-1-{[5-(hydroxymethyl)-6-methoxy-2-naphthyl]methyl}-6-oxopiperidin-2-yl)ethyl]-3,7-Dimethyl-1,2,3,7,8,8a-hexahydronaphthalen-1-yl2-Methylbutanoate | 1 | 1 |
| AB8 | (1s,3r,8as)-8-(2-{(4s,6s)-3-(4-hydroxy-3-Methoxybenzyl)-4-[2-(methylamino)-2-oxoethyl]-2-oxo-1,3-oxazinan-6-yl}ethyl)-3,7-dimethyl-1,2,3,7,8,8a-Hexahydronaphthalen-1-yl(2r)-2-methylbutanoate | 1 | 1 |
| ABB | (2s,4r)-1-acetyl-n-[(1s)-4-[(aminoiminomethyl)amino]-1-(2-benzothiazolylcarbonyl)butyl]-4-hydroxy-2-Pyrrolidinecarboxamide | 1 | 1 |
| ABF | Beta-d-arabinofuranose-5'-phosphate | 1 | 1 |
| ABI | 5-amidino-benzimidazole | 1 | 1 |
| ABU | Gamma-amino-butanoicAcid | 1 | 1 |
| ABX | 5-[1-(acetylamino)-3-methylbutyl]-4-(methoxycarbonyl)Proline | 1 | 1 |
| AC2 | 9-hyroxyethoxymethylguanine | 1 | 1 |
| ACC | N-[n-[2-amino-6-oxo-hexanoicAcid-6-yl]cysteinyl]-s-Methylcysteine | 1 | 1 |
| ACG | ModifiedAcarbosePentasaccharide | 1 | 1 |
| ACS | 1-[(1s)-carboxy-2-(methylsulfinyl)ethyl]-(3r)-[(5s)-5-Amino-5-carboxypentanamido]-(4r)-sulfanylazetidin-2-one | 1 | 1 |
| ADI | 2',3'-dideoxyadenosine-5'-diphosphate | 1 | 1 |
| ADQ | Adenosine-5'-diphosphate-glucose | 1 | 1 |
| ADZ | 9-methyl-9h-purin-6-amine | 1 | 1 |
| AEB | Aeruginosin98-b | 1 | 1 |
| AEJ | (1s)-1-{4-[(9ar)-octahydro-2h-pyrido[1,2-a]pyrazin-2-Yl]phenyl}-2-phenyl-1,2,3,4-tetrahydroisoquinolin-6-ol | 1 | 1 |
| AEP | 3-[(1-amino-2-carboxy-ethyl)-hydroxy-phosphinoyl]-2-Methyl-propionicAcid | 1 | 1 |
| AGB | N-(1-adamantyl)-n'-(4-guanidinobenzyl)urea | 1 | 1 |
| AGF | O-(((1r)-((n-(phenyl-methoxy-carbonyl)-alanyl)-amino)Methyl)hydroxyphosphinyl)3-l-phenyllactate | 1 | 1 |
| AH1 | Aha001 | 1 | 1 |
| AHC | 4-aminohydrocinnamicAcid | 1 | 1 |
| AHF | 2-[4-(hydroxy-methoxy-methyl)-benzyl]-7-(4-Hydroxymethyl-benzyl)-1,1-dioxo-3,6-bis-phenoxymethyl-1lambda6-[1,2,7]thiadiazepane-4,5-diol | 1 | 1 |
| AHR | Alpha-l-arabinofuranose | 1 | 1 |
| AHU | 1',5'-anhydro-2',3'-dideoxy-2'-(5-iodouracil-1-yl)-d-Ababino-hexitol | 1 | 1 |
| AHX | Seryl-hydroxamate-adenosineMonophosphate | 1 | 1 |
| AHZ | AdenosineDiphosphate5-(beta-ethyl)-4-methyl-thiazole-2-carboxylicAcid | 1 | 1 |
| AIC | (2s,5r,6r)-6-{[(2r)-2-amino-2-phenylethanoyl]amino}-3,3-dimethyl-7-oxo-4-thia-1-azabicyclo[3.2.0]heptane-2-CarboxylicAcid | 1 | 1 |
| AIJ | (2s,3r)-3-(4-hydroxyphenyl)-2-(4-{[(2s)-2-pyrrolidin-1-ylpropyl]oxy}phenyl)-2,3-dihydro-1,4-benzoxathiin-6-Ol | 1 | 1 |
| AIN | 2-(acetyloxy)benzoicAcid | 1 | 1 |
| AIR | 5-aminoimidazoleRibonucleotide | 1 | 1 |
| AKA | 10-decarboxymethylaclacinomycinA(dcmaa) | 1 | 1 |
| AKT | 10-decarboxymethylaclacinomycinT(dcmat) | 1 | 1 |
| AL1 | 3,,4-dihydro-2-(3-methoxyphenyl)-2h-thieno-[3,2-e]-1,2-thiazine-6-sulfonamide-1,1-dioxide | 1 | 1 |
| AL2 | 3,4-dihydro-4-hydroxy-2-(4-methoxyphenyl)-2h-thieno[3,2-e]-1,2-thiazine-6-sulfonamide-1,1-dioxide | 1 | 1 |
| AL3 | 3,4-dihydro-4-hydroxy-2-(2-thienymethyl)-2h-thieno[3,2-e]-1,2-thiazine-6-sulfonamide-1,1-dioxide | 1 | 1 |
| AL4 | (r)-4-ethylamino-3,4-dihydro-2-(2-methoylethyl)-2h-Thieno[3,2-e]-1,2-thiazine-6-sulfonamide-1,1-dioxide | 1 | 1 |
| AL5 | Thiophene-2,5-disulfonicAcid2-amide-5-(4-methyl-Benzylamide) | 1 | 1 |
| AL6 | 2-(3-methoxyphenyl)-2h-thieno-[3,2-e]-1,2-thiazine-6-Sulfinamide-1,1-dioxide | 1 | 1 |
| AL7 | (s)-3,4-dihydro-2-(3-methoxyphenyl)-4-methylamino-2h-Thieno[3,2-e]-1,2-thiazine-6-sulfonamide-1,1-dioxide | 1 | 1 |
| AL9 | N-[(4-methoxyphenyl)methyl]2,5-thiophenedesulfonamide | 1 | 1 |
| ALE | AdrL-epinephrine | 1 | 1 |
| ALH | 6-phenyl[5h]pyrrolo[2,3-b]pyrazine | 1 | 1 |
| ALJ | Cyclo-(l-arginine-l-proline)Inhibitor | 1 | 1 |
| ALL | D-allopyranose | 1 | 1 |
| ALR | Alrestatin | 1 | 1 |
| ALZ | 2-[n'-(4-amino-butyl)-hydrazinocarbonyl]-pyrrolidine-1-carboxylicAcidBenzylEster | 1 | 1 |
| AMC | Aminomethylcyclohexane | 1 | 1 |
| AME | N-acetylmethionine | 1 | 1 |
| AMH | Trans-4-aminomethylcyclohexane-1-carboxylicAcid | 1 | 1 |
| AML | Amylamine | 1 | 1 |
| AMR | 3,5-diamino-n-(aminoiminomethyl)-6-Chloropyrazinecarboxamide | 1 | 1 |
| AMT | 2-aminothiazole | 1 | 1 |
| AMX | AmidocarboxymethyldethiaCoenzyme*a | 1 | 1 |
| ANC | Anthracen-1-ylamine | 1 | 1 |
| ANN | 4-methoxybenzoicAcid | 1 | 1 |
| AO1 | (2s,3r)-3-amino-2-hydroxy-5-(ethylsulfanyl)pentanoyl-((s)-(-)-(1-naphthyl)ethyl)amide | 1 | 1 |
| AO2 | N'-(2s,3r)-3-amino-4-cyclohexyl-2-hydroxy-butano-n-(4-Methylphenyl)hydrazide | 1 | 1 |
| AO5 | N'-((2s,3r)-3-amino-2-hydroxy-5-(isopropylsulfanyl)Pentanoyl)-n-3-chlorobenzoylHydrazide | 1 | 1 |
| AOA | (aminooxy)aceticAcid | 1 | 1 |
| AP1 | {3-[3-(3,4-dimethoxy-phenyl)-1-(1-{1-[2-(3,4,5-Trimethoxy-phenyl)-butyryl]-piperidin-2yl}-vinyloxy)-Propyl]-phenoxy}-aceticAcid | 1 | 1 |
| API | 2,6-diaminopimelicAcid | 1 | 1 |
| APL | N-(1-benzyl-3,3,3-trifluoro-2,2-dihydroxy-propyl)-Acetamide | 1 | 1 |
| APQ | 2,6-diamino-8-propylsulfanylmethyl-3h-quinazoline-4-one | 1 | 1 |
| APT | 2-amino-6-[(4-carboxy-phenylamino)-methyl]-4-hydroxy-Pteridin-1-ium | 1 | 1 |
| APV | 6-(5-amino-5-carboxy-pentanoylamino)-3-hydroxymethyl-7-oxo-4-thia-1-aza-bicyclo[3.2.0]heptane-2-carboxylicAcid | 1 | 1 |
| APZ | 4-aminophthalhydrazide | 1 | 1 |
| AQO | 2-aminoquinazolin-4(3h)-one | 1 | 1 |
| AR3 | Cytarabine | 1 | 1 |
| ARH | 3-(hydroxymethyl)-1-methyl-5-(2-methylaziridin-1-yl)-2-phenyl-1h-indole-4,7-dione | 1 | 1 |
| ARL | 7-(3,5-ditert-butylphenyl)-3-methylocta-2,4,6-trienoicAcid | 1 | 1 |
| ARQ | Benzoylamino-benzyl-methyl-[2-hydroxy-3-[1-methyl-Ethyl-oxy-n-formamidyl]-4-phenyl-butyl]-ammonium | 1 | 1 |
| ASE | N-acetylSerotonin | 1 | 1 |
| ASN | Asparagine | 1 | 1 |
| ASO | 1,5-anhydrosorbitol | 1 | 1 |
| AT1 | 2-amino-3-(5-tert-butyl-3-(phosphonomethoxy)-4-Isoxazolyl)propionicAcid | 1 | 1 |
| ATC | 9-(n,n-dimethylglycylamido)-6-deoxy-6-demethyl-Tetracycline | 1 | 1 |
| ATF | PhosphodifluoromethylphosphonicAcid-adenylateEster | 1 | 1 |
| ATH | 4-hydroxy-aconitateIon | 1 | 1 |
| ATI | N-(3-amino-2-hydroxy-5-methylhexanoyl)ValylvalylasparticAcid | 1 | 1 |
| ATN | N-[(2s,3r)-3-amino-2-hydroxy-4-phenyl-butanoyl]-l-Prolyl-l-prolyl-l-alaninamide | 1 | 1 |
| ATO | Chloroacetone | 1 | 1 |
| ATQ | 2-aminothiazoline | 1 | 1 |
| ATU | 9-nitro-5,12-dihydro-7h-benzo[2,3]azepino[4,5-b]indol-6-one | 1 | 1 |
| AXP | 4-acetamido-2,4-didexoy-d-glycero-beta-d-galacto-OctopyranosylphosphonicAcid(anAxialPhosphonate) | 1 | 1 |
| AYM | 3-(6-aminopyridin-3-yl)-n-methyl-n-[(1-methyl-1h-indol-2-yl)methyl]acrylamide | 1 | 1 |
| AZ1 | AzelaicAcid | 1 | 1 |
| AZ2 | (2s)-2-ethoxy-3-[4-(2-{4-[(methylsulfonyl)oxy]phenyl}Ethoxy)phenyl]propanoicAcid | 1 | 1 |
| AZC | N-acetyl-n'-beta-d-glucopyranosylUrea | 1 | 1 |
| AZD | 3'-azido-3'-deoxythymidine-5'-diphosphate | 1 | 1 |
| AZL | 1-ethoxycarbonyl-d-phe-pro-2(4-aminobutyl)hydrazine | 1 | 1 |
| B1L | 3-[(4-hydroxybenzoyl)amino]azepan-4-yl4-Hydroxybenzoate | 1 | 1 |
| B1V | 2-[(2-oxo-2-piperidin-1-ylethyl)sulfanyl]-6-(trifluoromethyl)pyrimidin-4-ol | 1 | 1 |
| B3N | 4-dimethylamino-n-(6-hydroxycarbamoyethyl)benzamide-n-Hydroxy-7-(4-dimethylaMinobenzoyl)aminoheptanamide | 1 | 1 |
| B3P | 2-[3-(2-hydroxy-1,1-dihydroxymethyl-ethylamino)-Propylamino]-2-hydroxymethyl-propane-1,3-diol | 1 | 1 |
| B4G | Galactotriose | 1 | 1 |
| B8L | 3-[(3-sec-butyl-4-hydroxybenzoyl)amino]azepan-4-yl4-(2-hydroxy-5-methoxybenzoyl)benzoate | 1 | 1 |
| BA3 | Bis(adenosine)-5'-triphosphate | 1 | 1 |
| BAG | N-[(e)-2-amino-1-(3-{[amino(imino)methyl]amino}propyl)-2-hydroxyvinyl]benzamide | 1 | 1 |
| BAL | Beta-alanine | 1 | 1 |
| BB1 | 2-[(formyl-hydroxy-amino)-methyl]-hexanoicAcid(1-Dimethylcarbamoyl-2,2-dimethyl-propyl)-amide | 1 | 1 |
| BB3 | 3-(3,5-dibromo-4-hydroxy-benzoyl)-2-ethyl-benzofuran-6-sulfonicAcidDimethylamide | 1 | 1 |
| BBA | 2,7-bis-(4-amidinobenzylidene)-cycloheptan-1-one | 1 | 1 |
| BBH | 1-benzyl-3-(4-methoxy-benzenesulfonyl)-6-oxo-hexahydro-pyrimidine-4-carboxylicAcidHydroxyamide | 1 | 1 |
| BBM | 5-bromo-n-(2,3-dihydroxypropoxy)-3,4-difluoro-2-[(2-Fluoro-4-iodophenyl)amino]benzamide | 1 | 1 |
| BBT | 2-hydroxy-5-[4-(2-hydroxy-ethyl)-piperidin-1-yl]-5-Phenyl-1h-pyrimidine-4,6-dione | 1 | 1 |
| BCH | 2-(butyrylsulfanyl)-n,n,n-trimethylethanaminium | 1 | 1 |
| BDF | Beta-d-fructopyranose | 1 | 1 |
| BDH | L-Beta-Aspartylhistidine | 1 | 1 |
| BDI | 2-butyl-5,6-dihydro-1h-imidazo[4,5-d]pyridazine-4,7-Dione | 1 | 1 |
| BDS | 2,3-bis-benzo[1,3]dioxol-5-ylmethyl-succinicAcid | 1 | 1 |
| BE3 | N,n-[2,5-o-di-3-fluoro-benzyl-glucaryl]-di-[1-amino-Indan-2-ol] | 1 | 1 |
| BE4 | (2r,3r,4r,5r)-2,5-bis[(2,4-difluorobenzyl)oxy]-3,4-Dihydroxy-n,n'-bis[(1r,2s)-2-hydroxy-2,3-dihydro-1h-Inden-1-yl]hexanediamide | 1 | 1 |
| BEC | [5-(2-hydroxy-indan-1-ylcarbamoyl)-3,4-dihydroxy-2,5-[dibenzyl-oxy]-pentanoyl]-valinyl-amido-methane | 1 | 1 |
| BED | N,n-[2,5-o-di-2-fluoro-benzyl-glucaryl]-di-[1-amino-Indan-2-ol] | 1 | 1 |
| BEE | N,n-[2,5-o-[di-4-thiophen-3-yl-benzyl]-glucaryl]-di-[valyl-amido-methane] | 1 | 1 |
| BEG | 2,5-dibenzyloxy-3-hydroxy-hexanedioicAcidBis-[(2-Hydroxy-indan-1-yl)-amide] | 1 | 1 |
| BEH | 2,5-dibenzyloxy-3,4-dihydroxy-hexanedioicAcidBenzylamide(2-hydroxy-indan-1-yl)-amide | 1 | 1 |
| BEI | N,n-[2,5-o-[dibenzyl]-glucaryl]-di-[isoleucyl-amido-Methane] | 1 | 1 |
| BEJ | N,n-[2,5-o-[di-4-pyridin-3-yl-benzyl]-glucaryl]-di-[valyl-amido-methane] | 1 | 1 |
| BEL | 2,4-dinitro,5-[bis(2-bromoethyl)amino]-n-(2',3'-Dioxopropyl)benzamide | 1 | 1 |
| BEP | 1-isobutoxy-2-pyrrolidino-3[n-benzylanilino]Propane | 1 | 1 |
| BFI | 2[4-bromo-2-fluorophenyl)methyl]-6-Fluorospiro[isoquinoline-4-(1h),3'-pyrrolidine]-1,2',3,5'(2h)-tetrone | 1 | 1 |
| BFL | 2-(1,1'-biphenyl-4-yl)propanoicAcid | 1 | 1 |
| BFS | N-[1-(4-bromophenyl)ethyl]-5-fluoroSalicylamide | 1 | 1 |
| BGC | Beta-d-glucose | 1 | 1 |
| BGL | B-2-octylglucoside | 1 | 1 |
| BGN | N-butanoyl-2-amino-2-deoxy-glucopyranoside | 1 | 1 |
| BGP | Beta-galactose-6-phosphate | 1 | 1 |
| BH0 | 3-({5-benzyl-6-hydroxy-2,4-bis-(4-hydroxy-benzyl)-3-Oxo-[1,2,4]-triazepane-1-sulfonyl)-benzonitrile | 1 | 1 |
| BH7 | E-aminoBiotinylCaproicAcid | 1 | 1 |
| BHB | 2,6-diamino-8-(2-dimethylaminoethylsulfanylmethyl)-3h-Quinazolin-4-one | 1 | 1 |
| BHC | BenzeneHexacarboxylicAcid | 1 | 1 |
| BHH | N-butyl-n'-hydroxyguanidine | 1 | 1 |
| BHI | 4-bromo-3-hydroxy-3-methylButylDiphosphate | 1 | 1 |
| BHL | 6-Bromohexan-1-Ol | 1 | 1 |
| BI3 | 3-[1-(3-aminopropyl)-1h-indol-3-yl]-4-(1h-indol-3-yl)-1h-pyrrole-2,5-dione | 1 | 1 |
| BIN | 2,3-dicarboxy-4-(2-chloro-phenyl)-1-ethyl-5-Isopropoxycarbonyl-6-methyl-pyridinium | 1 | 1 |
| BIR | N-[3-[(1-aminoethyl)(hydroxy)phosphoryl]-2-(1,1'-Biphenyl-4-ylmethyl)propanoyl]alanine | 1 | 1 |
| BIS | 1,1,5,5-tetrafluorophosphopentylphosphonicAcidAdenylateEster | 1 | 1 |
| BIT | (-)-1-Phenyl-1,2,3,4-Tetrahydro-4-Hydroxypyrrolo[2,3 B]-7-Methylquinolin-4-One | 1 | 1 |
| BLI | 4-oxo-2-phenylmethanesulfonyl-octahydro-pyrrolo[1,2-a]Pyrazine-6-carboxylicAcid[1-(n-hydroxycarbamimidoyl)-piperidin-4-ylmethyl]-amide | 1 | 1 |
| BLL | (2r,3r,4r,5r)-3,4-dihydroxy-n,n'-bis[(1s,2r)-2-hydroxy-2,3-dihydro-1h-inden-1-yl]-2,5-bis(2-phenylethyl)Hexanediamide | 1 | 1 |
| BLV | BiliverdinIxGammaChromophore | 1 | 1 |
| BMA | Beta-d-mannose | 1 | 1 |
| BMD | Butyramide | 1 | 1 |
| BMH | 5h-pyrazolo(1,2-a)(1,2,4)triazol-4-ium,6-((2-carboxy-6-(1-hydroxyethyl)-4-methyl-7-oxo-1-azabicyclo(3.2.0)Hept-2-en-3-yl)thio)-6,7-dihydro-,Hydroxide,InnerSalt,(4r-(4alpha,5beta,6beta(r*)))- | 1 | 1 |
| BML | 4-bromophenol | 1 | 1 |
| BMQ | 1-(5'-phospho-beta-d-ribofuranosyl)barbituricAcid | 1 | 1 |
| BMU | 1-(5-tert-butyl-2-methyl-2h-pyrazol-3-yl)-3-(4-chloro-Phenyl)-urea | 1 | 1 |
| BN1 | (s)-2-amino-3-[3-hydroxy-5-(2-methyl-2h-tetrazol-5-yl)Isoxazol-4-yl]propionicAcid | 1 | 1 |
| BND | Me-a-n-benzoyl-amino-9-deoxy-neu5ac | 1 | 1 |
| BNE | 2-[3-(3-ethyl-1-methyl-2-oxo-azepan-3-yl)-phenoxy]-4-[1-amino-1-(1-methyl-1h-imidizol-5-yl)-ethyl]-Benzonitrile | 1 | 1 |
| BNF | N-benzylformamide | 1 | 1 |
| BOM | Hexadeca-10,12-dien-1-ol | 1 | 1 |
| BOT | Benzothiazole | 1 | 1 |
| BP1 | 6-bromo-7h-purine | 1 | 1 |
| BP3 | 2'-chloro-biphenyl-2,3-diol | 1 | 1 |
| BP6 | 2',6'-dichloro-biphenyl-2,6-diol | 1 | 1 |
| BPG | 9-(4-hydroxybutyl)-n2-phenylguanine | 1 | 1 |
| BPO | 3-[(z)-amino(imino)methyl]-n-[2-(4-benzoyl-1-Piperidinyl)-2-oxo-1-phenylethyl]benzamide | 1 | 1 |
| BPP | (4-benzyl-piperidin-1-yl)-(5-amidinomethyl-3ah-indol-2-yl-methanone | 1 | 1 |
| BR4 | 6-phenyl-4(r)-(7-phenyl-heptanoylamino)-hexanoicAcid | 1 | 1 |
| BRC | 12-bromododecanoicAcid | 1 | 1 |
| BRD | 1-beta-ribofuranosyl-1,3-diazepinone | 1 | 1 |
| BRE | 2-biphenyl-4-yl-6-fluoro-3-methyl-quinoline-4-CarboxylicAcid | 1 | 1 |
| BRG | 8-bromoguanine | 1 | 1 |
| BRL | 2,4-thiazolidiinedione,5-[[4-[2-(methyl-2-Pyridinylamino)ethoxy]phenyl]methyl]-(9cl) | 1 | 1 |
| BRR | (2r)-2-{[formyl(hydroxy)amino]methyl}hexanoicAcid | 1 | 1 |
| BRT | 5-bromonicotinamide | 1 | 1 |
| BRU | 5-bromo-2'-deoxyuridine-5'-monophosphate | 1 | 1 |
| BRY | (2z)-5'-Bromo-2,3'-Biindole-2',3(1h,1'H)-DioneAmmonia | 1 | 1 |
| BRZ | 6-(1,1-dimethylallyl)-2-(1-hydroxy-1-methylethyl)-2,3-Dihydro-7h-furo[3,2-g]chromen-7-one | 1 | 1 |
| BSB | N-benzyl-4-sulfamoyl-benzamide | 1 | 1 |
| BT3 | 3-[4-(2-pyrrolidin-1-yl-ethoxy)-benzyl]-2-4-(2-Pyrrolidin-1-yl-ethoxy)-phenyl]-benzo[b]thiophen-6-ol | 1 | 1 |
| BTD | 5-bromothienyldeoxyuridine | 1 | 1 |
| BTL | BetaineAldehyde | 1 | 1 |
| BU1 | 1,4-butanediol | 1 | 1 |
| BU2 | 1,3-butanediol | 1 | 1 |
| BUQ | 4-hydroxy-2-butanone | 1 | 1 |
| BVD | 5-bromovinyldeoxyuridine | 1 | 1 |
| BYS | 2-benzo[1,3]dioxol-5-ylmethyl-3-benzyl-succinicAcid | 1 | 1 |
| BZC | 2-(3'-methoxyphenyl)Benzimidazole-4-carboxamide | 1 | 1 |
| BZF | Benzofuran | 1 | 1 |
| BZM | BenzoicAcidPhenylmethylester | 1 | 1 |
| BZO | CarbobenzoxyGroup | 1 | 1 |
| BZT | 3-(3-bromo-4-pyrrolidin-1-ylmethyl-benzyl)-2-[4-Pyrrolidin-1-yl-ethoxy)-phenyl]-benzo[b]thiophen-6-ol | 1 | 1 |
| C02 | 4-(5-benzenesulfonylamino-1-methyl-1h-benzoimidazol-2-Ylmethyl)-benzamidine | 1 | 1 |
| C24 | 3-({2-[(4-carbamimidoyl-phenylamino)-methyl]-3-methyl-3h-benzoimidazole-5-carbonyl}-pyridin-2-yl-amino)-PropionicAcidEthylEster | 1 | 1 |
| C26 | (2r)-2-ethyl-1-hexanesulfonicAcid | 1 | 1 |
| C2F | 5-methyl-5,6,7,8-tetrahydrofolicAcid | 1 | 1 |
| C2G | [cytidine-5'-phosphate]GlycerylphosphoricAcidEster | 1 | 1 |
| C3P | Cytidine-3'-monophosphate | 1 | 1 |
| C5G | [cytidine-5'-phosphate]-beta-glucosyl-phosphoricAcidEster | 1 | 1 |
| C60 | [[[3-(2-methyl-propane-2-sulfonyl)-1-benzenyl]-2-Propyl]-carbonyl-histidyl]-amino-[cyclohexylmethyl]-[2-hydroxy-4-isopropyl]-pentan-5-oicAcidButylamide | 1 | 1 |
| CAH | 5-exo-hydroxycamphor | 1 | 1 |
| CAR | CytosineArabinose-5'-phosphate | 1 | 1 |
| CAX | (2s,4s)-4-(2,2-dihydroxyethyl)-2,3,3-Trimethylcyclopentanone | 1 | 1 |
| CBA | N-pyridoxyl-2,3-dihydroxyasparticAcid-5-monophosphate | 1 | 1 |
| CBB | 1-(3-carbamimidoyl-benzyl)-1h-indole-2-carboxylicAcid3-carbamimidoyl-benzylester | 1 | 1 |
| CBF | C-(1-hydrogyl-beta-d-glucopyranosyl)Formamide | 1 | 1 |
| CBH | S-(d-carboxybutyl)-l-homocysteine | 1 | 1 |
| CBL | Chlorambucil | 1 | 1 |
| CBN | Clorobiocin | 1 | 1 |
| CBQ | 2-{4-[4-(4-chloro-phenoxy)-benzenesulfonyl]-tetrahydro-pyran-4-yl}-n-hydroxy-acetamide | 1 | 1 |
| CBT | N,n-bis(4-chlorobenzyl)-1h-1,2,3,4-tetraazol-5-amine | 1 | 1 |
| CC0 | (4-{2-acetylamino-2-[1-(3-carbamoyl-4-Cyclohexylmethoxy-phenyl)-ethylcarbamoyl}-ethyl}-2-Phosphono-phenoxy)-aceticAcid | 1 | 1 |
| CC1 | {4-[2-acetylamino-2-(3-carbamoyl-2-cyclohexylmethoxy-6,7,8,9-tetrahydro-5h-benzocyclohepten-5ylcarbamoyl)-Ethyl]-2-phosphono-phenyl}-phosphonicAcid | 1 | 1 |
| CCB | Cyclobutyl(cyclopentyl)formamide | 1 | 1 |
| CCE | 2-[(aminocarbonyl)oxy]-n,n,n-trimethylethanaminium | 1 | 1 |
| CCR | [n-[n-(4-methoxy-2,3,6-trimethylphenylsulfonyl)-l-Aspartyl]-d-(4-amidino-phenylalanyl)]-piperidine | 1 | 1 |
| CCV | 6-[3-hydroxy-2-(hydroxymethyl)propyl]-5-methyl-2,4(1h,3h)-pyrimidinedione | 1 | 1 |
| CDA | 2-(6-chloro-3-{[2,2-difluoro-2-(2-pyridinyl)ethyl]Amino}-2-oxo-1(2h)-pyrazinyl)-n-[(2-fluoro-6-Pyridinyl)methyl]acetamide | 1 | 1 |
| CDB | 2-(6-chloro-3-{[2,2-difluoro-2-(2-pyridinyl)ethyl]Amino}-2-oxo-1(2h)-pyrazinyl)-n-[(2-fluoro-3-methyl-6-Pyridinyl)methyl]acetamide | 1 | 1 |
| CDC | [2-cytidylate-o'-phosphonyloxyl]-ethyl-trimethyl-Ammonium | 1 | 1 |
| CDD | 2-(6-chloro-3-{[2,2-difluoro-2-(1-oxido-2-pyridinyl)Ethyl]amino}-2-oxo-1(2h)-pyrazinyl)-n-[(2-Fluorophenyl)methyl]acetamide | 1 | 1 |
| CDG | Methyl4,6-o-[(1r)-1-carboxyethylidene]-beta-d-Galactopyranoside | 1 | 1 |
| CDH | D-(l-a-aminoadipoyl)-l-cysteinyl-d-isodehydrovaline | 1 | 1 |
| CDI | 2c-methyl-d-erythritol2,4-cyclodiphosphate | 1 | 1 |
| CDT | 4-methylsulfanyl-2-ureido-butyricAcid | 1 | 1 |
| CDV | 3-methyl-2-ureido-butyricAcid | 1 | 1 |
| CEG | 4,6-o-(1-carboxyethylidene)-beta-d-glucose | 1 | 1 |
| CEI | N-[3-benzyl-5-(4-hydroxyphenyl)pyrazin-2-yl]-2-(4-Hydroxyphenyl)acetamide | 1 | 1 |
| CEL | 4-[5-(4-methylphenyl)-3-(trifluoromethyl)-1h-pyrazol-1-yl]benzenesulfonamide | 1 | 1 |
| CG | 4-amidinoindan-1-one-2'-amidinohydrazone | 1 | 1 |
| CGF | C-(1-azido-alpha-d-glucopyranosyl)Formamide | 1 | 1 |
| CGT | Carba-Glucotropaeolin | 1 | 1 |
| CH1 | 3'-deoxy-cytidine-5'-triphosphate | 1 | 1 |
| CHB | 3-chloro-4-hydroxybenzoicAcid | 1 | 1 |
| CHQ | Cyclo-(l-histidine-l-proline)Inhibitor | 1 | 1 |
| CHR | Neocarzinostatin-chromophore | 1 | 1 |
| CHY | Chymostatin | 1 | 1 |
| CIA | 6-benzo[1,3]dioxol-5-yl-2-methyl-2,3,6,7,12,12a-Hexahydro-pyrazino[1',2':1,6]pyrido[3,4-b]indole-1,4-Dione | 1 | 1 |
| CIG | 2-amino-6-chloropyrazine | 1 | 1 |
| CIP | Inositol-2-methylene-1,2-cyclic-monophosphate | 1 | 1 |
| CIU | N-cyclohexyl-n'-(4-iodophenyl)urea | 1 | 1 |
| CK1 | 4-(2,5-dichlorothien-3-yl)pyrimidin-2-amine | 1 | 1 |
| CK2 | 4-(2,4-dimethyl-1,3-thiazol-5-yl)pyrimidin-2-amine | 1 | 1 |
| CK4 | 4-(2,4-dimethyl-1,3-thiazol-5-yl)-n-[4-(trifluoromethyl)phenyl]pyrimidin-2-amine | 1 | 1 |
| CK6 | 4-[4-(4-methyl-2-methylamino-thiazol-5-yl)-pyrimidin-2-ylamino]-phenol | 1 | 1 |
| CK7 | [4-(2-amino-4-methyl-thiazol-5-yl)-pyrimidin-2-yl]-(3-Nitro-phenyl)-amine | 1 | 1 |
| CK8 | N-[4-(2,4-dimethyl-thiazol-5-yl)-pyrimidin-2-yl]-n',n'-dimethyl-benzene-1,4-diamine | 1 | 1 |
| CKI | N-(2-aminoethyl)-5-chloroisoquinoline-8-sulfonamide | 1 | 1 |
| CL3 | N-methyl-n-[3-(6-phenyl[1,2,4]triazolo[4,3-b]pyridazin-3-yl)phenyl]acetamide | 1 | 1 |
| CLQ | N4-(7-chloro-quinolin-4-yl)-n1,n1-diethyl-pentane-1,4-Diamine | 1 | 1 |
| CLT | 4-phenyl-butanoicAcid | 1 | 1 |
| CLW | Chlorzoxazone | 1 | 1 |
| CLZ | 5-chloryl-2,4,6-quinazolinetriamine | 1 | 1 |
| CMB | 1-(3-carbamimidoyl-benzyl)-4-methyl-1h-indole-2-CarboxylicAcid3,5-dichloro-benzylamide | 1 | 1 |
| CMM | 2-[2-(1-carbamimidoyl-piperidin-3-yl)-acetylamino]-3-{4-[2-(3-oxalyl-1h-indol-7-yl)ethyl]-phenyl}-propionicAcidMethylEster | 1 | 1 |
| CMS | CarbamoylSarcosine | 1 | 1 |
| CMU | 5-chloro-6-(1-(2-iminopyrrolidinyl)Methyl)Uracil | 1 | 1 |
| CMX | CarboxymethyldethiaCoenzyme*a | 1 | 1 |
| CNA | Carba-nicotinamide-adenine-dinucleotide | 1 | 1 |
| CND | 5-beta-d-ribofuranosylnicotinamideAdenineDinucleotide | 1 | 1 |
| CNO | 2-{4-[(3{2-[4-(1-carboxy-1-methyl-ethoxy)-phenyl]-Acetylamino}-phenylcarbamoyl)-methyl]-phenoxy}-2-Methyl-propionicAcid | 1 | 1 |
| CNP | 2-propenyl-n-acetyl-neuramicAcid | 1 | 1 |
| CNS | HexadecylOctanoate | 1 | 1 |
| COD | DephosphoCoenzymeA | 1 | 1 |
| COE | Furo[2,3d]pyrimidineAntifolate | 1 | 1 |
| COG | 2,4-diamino-6-[n-(2',5'-dimethoxybenzyl)-n-Methylamino]quinazoline | 1 | 1 |
| COI | 2-oxo-4-methylpentanoicAcid | 1 | 1 |
| COL | 2-(oxalyl-amino)-4,7-dihydro-5h-thieno[2,3-c]thiopyran-3-carboxylicAcid | 1 | 1 |
| COQ | 2,4-diamino-6-[n-(3',5'-dimethoxybenzyl)-n-Methylamino]pyrido[2,3-d]pyrimidine | 1 | 1 |
| CP | PhosphoricAcidMono(formamide)ester | 1 | 1 |
| CP6 | 5-(4-chloro-phenyl)-6-ethyl-pyrimidine-2,4-diamine | 1 | 1 |
| CPB | 2-(2-chloro-phenyl)-5,7-dihydroxy-8-(3-hydroxy-1-Methyl-piperidin-4-yl)-4h-benzopyran-4-one | 1 | 1 |
| CPM | S-(2-carboxy-3-phenylpropyl)thiodiimine-s-methane | 1 | 1 |
| CPR | 6-chloropurineRiboside,5'-monophosphate | 1 | 1 |
| CPZ | 4-(4-chlorophenyl)imidazole | 1 | 1 |
| CR1 | 1-deoxy-1-methoxycarbamido-beta-d-glucopyranose | 1 | 1 |
| CR3 | 2-{5-[amino(iminio)methyl]-1h-indol-2-yl}-6-(cyclopentyloxy)benzenolate | 1 | 1 |
| CRA | 1-deoxy-1-methoxycarbamido-beta-d-gluco-2-Heptulopyranosonamide | 1 | 1 |
| CRM | 3-[8-((2s,3s,4r)-2,3,4,5-tetrahydroxypentyl)-2,4,7-Trioxo-1,3,8-trihydropteridin-6-yl]propanoicAcid | 1 | 1 |
| CRN | N-[(e)-amino(imino)methyl]-n-methylglycine | 1 | 1 |
| CRZ | 4-(9h-carbazol-9-yl)butanoicAcid | 1 | 1 |
| CT7 | (5-chloropyrazolo[1,5-a]pyrimidin-7-yl)-(4-Methanesulfonylphenyl)amine | 1 | 1 |
| CTD | 3-deazacytidine | 1 | 1 |
| CTO | Triacetylchitotriose | 1 | 1 |
| CTS | Castanospermine | 1 | 1 |
| CTZ | C2-hydroxy-coelenterazine | 1 | 1 |
| CXA | Phenylalanine-n-sulfonamide | 1 | 1 |
| CXL | Cyclohexanol | 1 | 1 |
| CXP | CyclohexanePropionicAcid | 1 | 1 |
| CXT | Carboxyatractyloside | 1 | 1 |
| CYC | Phycocyanobilin | 1 | 1 |
| CYH | Cyclohexanone | 1 | 1 |
| CZB | (2s,8r)-8-benzyl-2-(4-bromobenzyl)-2-hydroperoxy-6-(4-Hydroxyphenyl)-7,8-dihydroimidazo[1,2-a]pyrazin-3(2h)-One | 1 | 1 |
| CZI | (2r)-8-benzyl-2-hydroperoxy-6-(4-hydroxyphenyl)-2-(4-Iodobenzyl)-7,8-dihydroimidazo[1,2-a]pyrazin-3(2h)-one | 1 | 1 |
| CZN | (2s,8r)-8-benzyl-2-hydroperoxy-6-(4-hydroxyphenyl)-2-(2-naphthylmethyl)-7,8-dihydroimidazo[1,2-a]pyrazin-3(2h)-one | 1 | 1 |
| CZP | (8r)-8-(cyclopentylmethyl)-2-hydroperoxy-2-(4-Hydroxybenzyl)-6-(4-hydroxyphenyl)-7,8-Dihydroimidazo[1,2-a]pyrazin-3(2h)-one | 1 | 1 |
| D1L | 2-[4-(2,4-dichlorophenoxy)phenoxy]propanoicAcid | 1 | 1 |
| D4T | 2',3'-dehydro-2',3'-deoxy-thymidine5'-triphosphate | 1 | 1 |
| D6G | 2-deoxy-glucose-6-phosphate | 1 | 1 |
| D6P | (3,4,5,7-tetrahydroxy-hept-1-enyl)-phosphonicAcid | 1 | 1 |
| D76 | (2r)-4-[(6-chloro-2-naphthyl)sulfonyl]-1-[(5-methyl-4,5,6,7-tetrahydro[1,3]thiazolo[5,4-c]pyridin-2-yl)Carbonyl]piperazine-2-carboxamide | 1 | 1 |
| D7P | D-phenylalanyl-n~5~-[(2,2-dihydroxyhydrazino)(imino)Methyl]-d-ornithinamide | 1 | 1 |
| DA2 | Ng,ng-dimethyl-l-arginine | 1 | 1 |
| DA3 | (2s,5',s)-2-amino-3-(3-carboxy-2-isoxazolin-5-yl)PropanoicAcid | 1 | 1 |
| DAH | 3,4-dihydroxyphenylalanine | 1 | 1 |
| DAV | Delta-aminoValericAcid | 1 | 1 |
| DB1 | 2,3-dihydroxybenzamide | 1 | 1 |
| DBD | 7-(1,1-dioxo-1h-benzo[d]isothiazol-3-yloxymethyl)-2-(oxalyl-amino)-4,7-dihydro-5h-thieno[2,3-c]pyran-3-CarboxylicAcid | 1 | 1 |
| DBH | 2,3-dihydroxy-benzoicAcid | 1 | 1 |
| DBM | 9-(6-deoxy-beta-d-allofuranosyl)-6-methylpurine | 1 | 1 |
| DCA | Desulfo-coenzymeA | 1 | 1 |
| DCD | DiethylcarbamodithioicAcid | 1 | 1 |
| DCN | Diclosan | 1 | 1 |
| DCU | 3,8-diamino-5,10'-(trimethylammonium)decyl-6-phenylPhenanthridinium | 1 | 1 |
| DCY | D-cysteine | 1 | 1 |
| DDC | 7-hydroxy-2-phenyl-chroman-4-one | 1 | 1 |
| DDN | 3,4-dihydro-2'-deoxyuridine-5'-monophosphate | 1 | 1 |
| DDQ | Decylamine-n,n-dimethyl-n-oxide | 1 | 1 |
| DDT | N,o-didansyl-l-tyrosine | 1 | 1 |
| DDY | 2',3'-dideoxycytosine-5'-diphospate | 1 | 1 |
| DED | 2-dimethylamino-ethyl-diphosphate | 1 | 1 |
| DEE | 3,5-dimethyl-1h-pyrazole-4-carboxylicAcidEthylEster | 1 | 1 |
| DEL | 4-deoxylactose | 1 | 1 |
| DEO | 2-[2-(1,3-dioxo-1,3-dihydro-2h-isoindol-2-yl)ethyl]-4-(4'-ethoxy-1,1'-biphenyl-4-yl)-4-oxobutanoicAcid | 1 | 1 |
| DER | D-4-phosphoerythronicAcid | 1 | 1 |
| DEZ | 4-phospho-d-erythronate | 1 | 1 |
| DF2 | (2r)-3-{[(4z)-5,6-Diphenyl-6,7-Dihydro-4h Pyrrolo[2,3-D]Pyrimidin-4-Ylidene]Amino}Propane1,2-Diol | 1 | 1 |
| DFL | 2-(4-hydroxy-phenyl)-chroman-4-one | 1 | 1 |
| DFN | 3-[3-(2,3-dihydroxy-propylamino)-phenyl]-4-(5-fluoro-1-methyl-1h-indol-3-yl)-pyrrole-2,5-dione | 1 | 1 |
| DFR | 3-deoxy-3-methyl-d-fructose | 1 | 1 |
| DFW | N-(5,6-Diphenylfuro[2,3-D]Pyrimidin-4-Yl)Glycine | 1 | 1 |
| DFY | (5,6-Diphenyl-Furo[2,3-D]Pyrimidin-4-Ylamino)-Acetic | 1 | 1 |
| DFZ | 3-Amino-3-Benzyl-[4.3.0]Bicyclo-1,6Diazanonan-2-One | 1 | 1 |
| DG6 | 2-deoxy-glucitol-6-phosphate | 1 | 1 |
| DGN | D-glutamine | 1 | 1 |
| DGT | 2'-deoxyguanosine-5'-triphosphate | 1 | 1 |
| DHF | DihydrofolicAcid | 1 | 1 |
| DHG | PhosphonicAcid2-dodecanoylamino-hexylEsterPropylEster | 1 | 1 |
| DHH | (S)-2-Amino-7,7-DihydroxyheptanoicAcid | 1 | 1 |
| DHM | 2,6-dimethyl-7-octen-2-ol | 1 | 1 |
| DHP | 3-decyl-2,5-dioxo-4-hydroxy-3-pyrroline | 1 | 1 |
| DHQ | 3,4-dihydro-5-methyl-isoquinolinone | 1 | 1 |
| DHZ | 3,4-dihydro-1h-pyrimidin-2-oneNucleoside | 1 | 1 |
| DIA | Octane1,8-diamine | 1 | 1 |
| DIG | 2,5-dideoxy-2,5-imino-d-glucitol | 1 | 1 |
| DIR | 3-{[(E)-Amino(Hydroxyimino)Methyl]Amino}Alanine | 1 | 1 |
| DK1 | 5,7-dichloro-4-hydroxyquinoline-2-carboxylicAcid | 1 | 1 |
| DLY | D-lysine | 1 | 1 |
| DMB | 2-((3',5'-dimethyl-4'-hydroxyphenyl)azo)benzoicAcid | 1 | 1 |
| DMG | N,n-dimethylglycine | 1 | 1 |
| DMV | 2,3-dihydroxy-valerianicAcid | 1 | 1 |
| DMZ | 4-methyl-n-methyl-n-(2-phenyl-2h-pyrazol-3-yl)Benzenesulfonamide | 1 | 1 |
| DNF | 2,4-dinitrophenol | 1 | 1 |
| DOC | 2',3'-dideoxycytidine-5'-monophosphate | 1 | 1 |
| DOR | (4s)-2,6-Dioxohexahydropyrimidine-4-CarboxylicAcid | 1 | 1 |
| DP2 | L-lysyl-n~5~-[(z)-(2,2-dihydroxyhydrazino)(imino)Methyl]-l-ornithinamide | 1 | 1 |
| DPB | (s)-1-[2'-deoxy-3',5'-o-(1-phosphono)benzylidene-b-d-Threo-pentofuranosyl]thymine | 1 | 1 |
| DPP | DiamminopropanoicAcid | 1 | 1 |
| DPX | Mono-{4-[(4-amino-2-methyl-pyrimidin-5-ylmethyl)-Amino]-2-hydroxy-3-mercapto-pent-3-enyl-phosphono}Ester | 1 | 1 |
| DPZ | 3,5-diaminophthalhydrazide | 1 | 1 |
| DQO | 1-(2,6-dichlorophenyl)-5-(2,4-difluorophenyl)-7-Piperazin-1-yl-3,4-dihydroquinazolin-2(1h)-one | 1 | 1 |
| DRG | 5,6-dihydro-benzo[h]cinnolin-3-ylamine | 1 | 1 |
| DRM | {[(1r,2s)-2-(2,4-dioxo-3,4-dihydropyrimidin-1(2h)-yl)Cyclopentyl]oxy}methylphosphonicAcid | 1 | 1 |
| DSN | D-serine | 1 | 1 |
| DSP | D-asparticAcid | 1 | 1 |
| DSS | MethylMethylsulfinylmethylSulfide | 1 | 1 |
| DST | DimethylallylS-thiolodiphosphate | 1 | 1 |
| DTB | 6-(5-methyl-2-oxo-imidazolidin-4-yl)-hexanoicAcid | 1 | 1 |
| DTC | Bishydroxy[2h-1-benzopyran-2-one,1,2-benzopyrone] | 1 | 1 |
| DTP | 2'-deoxyadenosine5'-triphosphate | 1 | 1 |
| DTQ | 4-[3-hydroxyanilino]-6,7-dimethoxyquinazoline | 1 | 1 |
| DTR | D-tryptophan | 1 | 1 |
| DTY | D-tyrosine | 1 | 1 |
| DUC | Dihydropyrimidine-2,4(1h,3h)-dione | 1 | 1 |
| DUN | 2'-deoxyuridine5'-alpha,beta-imido-diphosphate | 1 | 1 |
| DUP | 2'-deoxyuridine5'-alpha,beta-imido-triphosphate | 1 | 1 |
| DUX | 2,3-Deoxy-3-Fluoro-5-O-Trityluridine | 1 | 1 |
| DX9 | (+)-2-[4-[((s)-1-acetimidoyl-3-pyrrodinyl)oxy]-3-(7-Amidino-2-napthyl)propionicAcid | 1 | 1 |
| DXG | 4-deoxyglucarate | 1 | 1 |
| DXX | MethylmalonicAcid | 1 | 1 |
| DZF | 5-deazafolicAcid | 1 | 1 |
| DZN | Daidzin | 1 | 1 |
| E09 | 3-hydroxymethyl-5-aziridinyl-1methyl-2-[1h-indole-4,7-Dione]-propanol | 1 | 1 |
| E10 | (s,s)-(-)-n,n'-di-5'-[5',6',7',8'-tetrahydro-2'(1'h)-Quinolynyl]-1,10-diaminodecaneDihydrochloride | 1 | 1 |
| E12 | (s,s)-(-)-n,n'-di-5'-[5',6',7',8'-tetrahydro-2'(1'h)-Quinolynyl]-1,12-diaminododecaneDihydrochloride | 1 | 1 |
| E1P | EthylOxo(piperidin-1-yl)acetate | 1 | 1 |
| E20 | 1-benzyl-4-[(5,6-dimethoxy-1-indanon-2-yl)methyl]Piperidine | 1 | 1 |
| E4D | (2s,3r)-2-(4-(2-(piperidin-1-yl)ethoxy)phenyl)-2,3-Dihydro-3-(4-hydroxyphenyl)benzo[b][1,4]oxathiin-6-ol | 1 | 1 |
| EA1 | 3h-imidazo[2,1-i]purine | 1 | 1 |
| EAA | EthacrynicAcid | 1 | 1 |
| EAL | 1-((2s)-2-{[(1s)-1-carboxy-3-phenylpropyl]amino}Propanoyl)-l-proline | 1 | 1 |
| EBP | Diethyl4-methylbenzylphosphonate | 1 | 1 |
| EDR | EdrophoniumIon | 1 | 1 |
| EDT | {[-(bis-carboxymethyl-amino)-ethyl]-carboxymethyl-Amino}-aceticAcid | 1 | 1 |
| EFS | EthylDihydrogenPhosphate | 1 | 1 |
| EG1 | Aminomethylenecarbonylaminodi(ethyloxy)Ethylaminocarbonylbenzenesulfonamide | 1 | 1 |
| EG2 | Aminodi(ethyloxy)ethylaminocarbonylbenzenesulfonamide | 1 | 1 |
| EGT | 2-(3,4,5-trihydroxy-phenyl)-chroman-3,5,7-triol | 1 | 1 |
| EIP | 4-hydroxy-3-methylButylDiphosphate | 1 | 1 |
| EMU | N-Benzyl-9h-Purin-6-Amine | 1 | 1 |
| ENO | 3-(4-hydroxy-phenyl)pyruvicAcid | 1 | 1 |
| ENP | Etheno-nadp | 1 | 1 |
| EPC | (1s)-1-[((1e)-{3-hydroxy-2-methyl-5-[(phosphonooxy)Methyl]pyridin-4-yl}methylene)amino]ethylphosphonicAcid | 1 | 1 |
| EPS | 5-[(1-carboxyvinyl)oxy]-4-hydroxy-3-(phosphonooxy)Cyclohex-1-ene-1-carboxylicAcid | 1 | 1 |
| ESX | Benzo[b]thiophene-2-carboxamidine | 1 | 1 |
| ETC | (r,r)-5,11-cis-diethyl-5,6,11,12-tetrahydrochrysene-2,8-diol | 1 | 1 |
| ETR | N-ethylRetinamide | 1 | 1 |
| ETS | (4s-trans)-4-(ethylamino)-5,6-dihydro-6-methyl-4h-Thieno(2,3-b)thiopyran-2-sulfonamide-7,7-dioxide | 1 | 1 |
| ETX | 2-ethoxyethanol | 1 | 1 |
| EUG | 2-methoxy-4-vinyl-phenol | 1 | 1 |
| FA3 | 2-anhydro-3-fluoro-quinicAcid | 1 | 1 |
| FA6 | 3-hydroxyiminoQuinicAcid | 1 | 1 |
| FAC | 1,1,1,3,3,3-hexafluoropropanediol | 1 | 1 |
| FAM | Alpha-fluoro-amidocarboxymethyldethiaCoenzymeAComplex | 1 | 1 |
| FAS | Arabino-flavin-adenineDinucleotide | 1 | 1 |
| FBA | 4-fluorobenzylamine | 1 | 1 |
| FBI | 7-[4-(4-fluoro-phenyl)-6-isopropyl-2-(methanesulfonyl-Methyl-amino)-pyrimidin-5-yl]-3,5-dihydroxy-heptanoicAcid | 1 | 1 |
| FBS | 4-flourobenzenesulfonamide | 1 | 1 |
| FBT | 2,6-difluorobenzenesulfonamide | 1 | 1 |
| FBU | 3,5-difluorobenzenesulfonamide | 1 | 1 |
| FCB | Beta-d-fucose | 1 | 1 |
| FCD | 5-(2-chlorophenyl)furan-2-carboxylicAcid | 1 | 1 |
| FCN | Fosfomycin | 1 | 1 |
| FCR | Alpha,alpha,alpha-trifluoro-p-cresol | 1 | 1 |
| FCX | Alpha-fluoro-carboxymethyldethiaCoenzymeAComplex | 1 | 1 |
| FD1 | N-alpha-(2-naphthylsulfonyl)-n-(3-amidino-l-Phenylalaninyl)-d-pipecolinicAcid | 1 | 1 |
| FD2 | N-alpha-(2-naphthylsulfonyl)-n(3-amidino-l-Phenylalaninyl)isopipecolinicAcidMethylEster | 1 | 1 |
| FD3 | N-alpha-(2-naphthylsulfonyl)-3-amidino-l-phenylalaninePiperazide | 1 | 1 |
| FD4 | N-alpha-(2-naphthylsulfonyl)-n(3-amidino-l-Phenylalaninyl)-4-acetyl-piperazine | 1 | 1 |
| FDI | 4-(n-acetylamino)-3-[n-(2-ethylbutanoylamino)]benzoicAcid | 1 | 1 |
| FDP | Fructose-2,6-diphosphate | 1 | 1 |
| FDQ | D-gluco-2,5-anhydro-1-deoxy-1-phosphonohexitol-6-Phosphate | 1 | 1 |
| FEN | N-(4-hydroxyphenyl)all-transRetinamide | 1 | 1 |
| FEP | [(4-{4-[4-(difluoro-phosphono-methyl)-phenyl]-butyl}-Phenyl)-difluoro-methyl]-phosphonicAcid | 1 | 1 |
| FEX | Methyl3-[3-(benzoyl{[4'-(dimethylamino)-1,1'-biphenyl-4-yl]methyl}amino)phenyl]propanoate | 1 | 1 |
| FFO | 5-formyl-6-hydrofolicAcid | 1 | 1 |
| FHB | 3-fluoro-4-hydroxybenzoicAcid | 1 | 1 |
| FID | (2s,4s)-2-aminoformyl-6-fluoro-spiro[chroman-4,4'-Imidazolidine]-2',5'-dione | 1 | 1 |
| FIL | (1e)-1-[3-(cyclopentyloxy)-4-methoxyphenyl]ethanoneO-(aminocarbonyl)oxime | 1 | 1 |
| FIR | (2r,4s)-2-aminoformyl-6-fluoro-spiro[chroman-4,4'-Imidazolidine]-2',5'-dione | 1 | 1 |
| FL8 | 6,4'-dihydroxy-3-methyl-3',5'-dibromoflavone | 1 | 1 |
| FLA | Trifluoroalanine | 1 | 1 |
| FLM | 3-fluoro-2-methyl-aniline | 1 | 1 |
| FLP | Flurbiprofen | 1 | 1 |
| FLU | 2-(6-hydroxy-3-oxo-3h-xanthen-9-yl)-benzoicAcid | 1 | 1 |
| FLV | Flaviolin | 1 | 1 |
| FLX | N-[(furan-2-yl)carbonyl]-(s)-leucyl-(r)-[1-amino-2(1h-Indol-3-yl)ethyl]-phosphonicAcid | 1 | 1 |
| FMD | 4-((3r,4s,5r)-4-amino-3,5-dihydroxy-hex-1-ynyl)-5-Fluoro-3-[1-(3-methoxy-1h-pyrrol-2-yl)-meth-(z)-Ylidene]-1,3-dihydro-indol-2-one | 1 | 1 |
| FME | N-formylmethionine | 1 | 1 |
| FMF | 2-deoxy-2-fluorohexopyranosylFluoride | 1 | 1 |
| FMM | N-{3-chloro-4-[(3-fluorobenzyl)oxy]phenyl}-6-[5-({[2-(methylsulfonyl)ethyl]amino}methyl)-2-furyl]-4-Quinazolinamine | 1 | 1 |
| FMS | TrifluoromethaneSulfonamide | 1 | 1 |
| FMX | Famoxadone | 1 | 1 |
| FNP | {[7-(difluoro-phosphono-methyl)-naphthalen-2-yl]-Difluoro-methyl}-phosphonicAcid | 1 | 1 |
| FOC | Fucitol | 1 | 1 |
| FPC | D-fructose-6-phosphate(openForm) | 1 | 1 |
| FPH | 3-(4-fluorophenyl)-2-pyridin-4-yl-1h-pyrrolo[3,2-b]Pyridin-1-ol | 1 | 1 |
| FPI | N-formylpiperidine | 1 | 1 |
| FPN | 4-Fluorophenol | 1 | 1 |
| FQP | 4-amino-2-trifluoromethyl-5-hydroxymethylpyrimidinePyrophosphate | 1 | 1 |
| FR0 | N''-(4-(5-((1h-benzimidazol-2-ylamino)methyl)-2-Thienyl)-1,3-thiazol-2-yl)guanidine | 1 | 1 |
| FR2 | 1-((1r)-1-(hydroxymethyl)-3-phenylpropyl)-1h-imidazole-4-carboxamide | 1 | 1 |
| FR3 | 1-((1r)-1-(hydroxymethyl)-3-(1-naphthyl)propyl)-1h-Imidazole-4-carboxamide | 1 | 1 |
| FR4 | 1-((1r)-1-(hydroxymethyl)-3-{6-[(3-phenylpropanoyl)Amino]-1h-indol-1-yl}propyl)-1h-imidazole-4-carboxamide | 1 | 1 |
| FR5 | 1-((1r)-1-(hydroxymethyl)-3-(6-((3-(1-methyl-1h-Benzimidazol-2-yl)propanoyl)amino)-1h-indol-1-yl)Propyl)-1h-imidazole-4-carboxamide | 1 | 1 |
| FR6 | 1-{(1r,2s)-2-hydroxy-1-[2-(1-naphthyl)ethyl]propyl}-1h-imidazole-4-carboxamide | 1 | 1 |
| FR7 | 1-{(1r,2s)-1-[2-(2,3,-dichlorophenyl)ethyl]-2-Hydroxypropyl}-1h-imidazole-4-carboxamide | 1 | 1 |
| FR8 | 1-((1r)-1-(hydroxymethyl)-3-{6-[(5-phenylpentanoyl)Amino]-1h-indol-1-yl}propyl)-1h-imidazole-4-carboxamide | 1 | 1 |
| FR9 | 1-[(1r)-3-(6-{[(benzylamino)carbonyl]amino}-1h-indol-1-yl)-1-(hydroxymethyl)propyl]-1h-imidazole-4-Carboxamide | 1 | 1 |
| FRC | 1-{(1r,2s)-2-hydroxy-1-[2-(2-naphthyloxy)ethyl]propyl}-1h-imidazone-4-carboxamide | 1 | 1 |
| FRG | 2-[3-methyl-4-(n-methyl-guanidino)-butyrylamino]-3-(4-Phenylethynyl-phenyl)-propionicAcidMethylEster | 1 | 1 |
| FRU | Fructose | 1 | 1 |
| FSN | (3asr,4rs,8asr,8brs)-4-(2-(4-fluorobenzyl)-1,3-Dioxodeacahydropyrrolo[3,4-a]Pyrrolizin-4-yl)Benzamidine | 1 | 1 |
| FSP | [1-(4-fluorobenzyl)cyclobutyl]methyl(1s)-1-[oxo(1h-Pyrazol-5-ylamino)acetyl]pentylcarbamate | 1 | 1 |
| FTA | 3-(4-phenylamino-phenylamino)-2-(1h-tetrazol-5-yl)-Acrylonitrile | 1 | 1 |
| FTH | 1-[2-(4-cyano-benzylamino)-3-(3-methyl-3h-imidazol-4-Yl)-propionyl]-5-naphthalen-1-yl-1,2,3,6-tetrahydro-Pyridine-4-carbonitrile | 1 | 1 |
| FTP | 3-(4-amino-2-trifluoromethyl-pyrimidin-5-ylmethyl)-4-Methyl-5-(2-phosphonatooxy-ethyl)-thiazol-3-ium | 1 | 1 |
| FUF | 2-deoxy-2-fluoro-beta-l-fucopyranose | 1 | 1 |
| FVF | O-((((n-phenyl-methoxy-carbonyl)-phenylAlanyl-Carbonyl)amino)-isobutyl)hydroxyPhosphinyl)-3-PhenylaceticAcid | 1 | 1 |
| FWD | 2-amino-3-(5-fluoro-2,4-dioxo-3,4-dihydro-2h-pyrimidin-1-yl)-propionicAcid | 1 | 1 |
| FXV | Methyl-3-(4'-n-oxopyridylphenoyl)-3-methyl-2-(m-Amidinobenzyl)-propionate | 1 | 1 |
| FXY | 1-methylheptylformamide | 1 | 1 |
| FYA | Adenosine-5'-[phenylalaninol-phosphate] | 1 | 1 |
| G2F | 2-deoxy-2fluoro-glucose | 1 | 1 |
| G6D | 6-deoxy-alpha-d-glucose | 1 | 1 |
| G6Q | Glucose-6-phosphate | 1 | 1 |
| GA2 | 9-(1,3-dihydroxy-propoxymethane)guanine | 1 | 1 |
| GAC | Dihydro-acarbose | 1 | 1 |
| GAG | 3-hydroxyisoxazole-4-carboxylicAcid | 1 | 1 |
| GAH | N-{[(2s)-1-(N-{[4-({[Amino(Imino)Methyl]Amino}Methyl) Cyclohexyl]Carbonyl}-3-Cyclohexyl-L-Alanyl)Azetidin-2 Yl]Carbonyl}-L-Tyrosyl-N~6~-[Amino(Imino)Methyl]-LLysinamide | 1 | 1 |
| GAN | 2-[3-benzyl-5-(1-alanyl-aminoethyl)-2,3,6,7-tetrahydro-1h-azepin-1-yl]-1-oxopropyl-valinyl-valine-methylester | 1 | 1 |
| GAR | GlycinamideRibonucleotide | 1 | 1 |
| GBD | 4-hydroxy-1,2,5-thiadiazole-3-carboxylicAcid | 1 | 1 |
| GBI | S-(3-iodobenzyl)glutathione | 1 | 1 |
| GCA | 6-(3',5'-dimethylbenzyl)-1-ethoxymethyl-5-Isopropyluracil | 1 | 1 |
| GCG | Bis(gamma-glutamyl-cysteinyl-glycinyl)spermidine | 1 | 1 |
| GCS | D-glucosamine | 1 | 1 |
| GCU | D-glucuronicAcid | 1 | 1 |
| GCW | 4-o-methyl-beta-d-glucuronicAcid | 1 | 1 |
| GDL | 2-acetamido-2-deoxy-d-glucono-1,5-lactone | 1 | 1 |
| GDR | Guanosine-5'-diphosphate-rhamnose | 1 | 1 |
| GDS | OxidizedGlutathioneDisulfide | 1 | 1 |
| GEO | Gemcitabine | 1 | 1 |
| GFP | 2-deoxy-2-fluoro-alpha-d-glucose-1-phosphate | 1 | 1 |
| GGB | L-canavanine | 1 | 1 |
| GHA | 1'-((1,4-dideoxy-1,4-imino-d-arabinitol)-4-n-ammonium)-1'-deoxy-l-erythritol-3'-sulfateInnerSalt | 1 | 1 |
| GIO | Cyclo-(glycine-l-proline)Inhibitor | 1 | 1 |
| GIP | S-(n-hydroxy-n-iodophenylcarbamoyl)glutathione | 1 | 1 |
| GL4 | 8,9,10-trihydroxy-7-hydroxymethyl-2-thioxo-6-oxa-1,3-Diaza-spiro[4.5]decan-4-one | 1 | 1 |
| GL5 | 3,8,9,10-tetrahydroxy-7-hydroxymethyl-6-oxa-1,3-diaza-Spiro[4.5]decane-2,4-dione | 1 | 1 |
| GL6 | (3,4,5-trihydroxy-6-hydroxymethyl-tetrahydro-pyran-2-Yl)-phosphoramidicAcidDimethylEster | 1 | 1 |
| GL7 | 8,9,10-trihydroxy-7-hydroxymethyl-3-methyl-6-oxa-1,3-Diaza-spiro[4.5]decane-2,4-dione | 1 | 1 |
| GL8 | GalanthamineDerivative | 1 | 1 |
| GL9 | N-(8,9,10-trihydroxy-7-hydroxymethyl-2,4-dioxo-6-oxa-1,3-diaza-spiro[4.5]dec-3-yl-acetamide | 1 | 1 |
| GLE | 1-decyl-3-trifluoroEthyl-sn-glycero-2-phosphomethanol | 1 | 1 |
| GLT | 5-deoxy-5-thio-alpha-d-glucose | 1 | 1 |
| GNB | S-p-nitrobenzyloxycarbonylglutathione | 1 | 1 |
| GNR | 2-(3,4-dihydro-3-oxo-2h-benzo[b][1,4]thiazin-2-yl)-n-Hydroxyacetamide | 1 | 1 |
| GOA | GlycolicAcid | 1 | 1 |
| GOM | Glutamol-amp | 1 | 1 |
| GP2 | PhosphomethylphosphonicAcidGuanosylEster | 1 | 1 |
| GP6 | 1-(4-amidinophenyl)-3-(4-chlorophenyl)urea | 1 | 1 |
| GP8 | -(2-amidinophenyl)-3-(phenoxyphenyl)urea | 1 | 1 |
| GPB | N-[4-[2-(2-amino-4-methyl-7h-pyrrolo[2,3-d]pyrimidin-5-yl)-ethyl]-benzoyl]glutamicAcid | 1 | 1 |
| GPG | Guanylyl-2',5'-phosphoguanosine | 1 | 1 |
| GPJ | Glyphosate | 1 | 1 |
| GPM | Glucopyranosyl-1-methyl-phosphonicAcid | 1 | 1 |
| GSB | S-benzyl-glutathione | 1 | 1 |
| GSE | L-alpha-glycerophosphorylserine | 1 | 1 |
| GTB | S-(p-nitrobenzyl)glutathione | 1 | 1 |
| GTD | 1-(s-glutathionyl)-2,5-trinitrocyclohexadiene | 1 | 1 |
| GTL | (2e,3r,4r,5r,6s)-3,4,5-trihydroxy-6-(hydroxymethyl)-2-Piperidinone | 1 | 1 |
| GTZ | (5r,6s,7s,8s)-5-hydroxymethyl-6,7,8-trihydroxy-Tetrazolo[1,5-a]piperidine | 1 | 1 |
| GU7 | 2-amino-7-[2-(2-hydroxy-1-hydroxymethyl-ethylamino)-Ethyl]-1,7-dihydro-purin-6-one | 1 | 1 |
| GUM | 4-methyl-umbelliferyl-n-acetyl-chitobiose | 1 | 1 |
| GW3 | 7-(1-ethyl-propyl)-7h-pyrrolo-[3,2-f]quinazoline-1,3-Diamine | 1 | 1 |
| GZZ | N-{8-[(8-{[(e)-amino(imino)methyl]amino}octyl)amino]Octyl}guanidine | 1 | 1 |
| H1N | N-[2-Oxo-3-((E)-Phenyl{[4-(Piperidin-1 Ylmethyl)Phenyl]Imino}Methyl)-2,6-Dihydro-1h-Indol 5-Yl]Ethanesulfonamide | 1 | 1 |
| H2B | 2-amino-6-(1,2-dihydroxy-propyl)-7,8-dihydro-6h-Pteridin-4-one | 1 | 1 |
| H4M | 5,10-dimethyleneTetrahydromethanopterin | 1 | 1 |
| H4P | 1-deoxy-6-o-phosphono-1-[(phosphonomethyl)amino]-l-Threo-hexitol | 1 | 1 |
| HAB | 2-((4'-hydroxyphenyl)-azo)benzoicAcid | 1 | 1 |
| HAX | N-hydroxyamidocarboxymethyldethiaCoenzyme*a | 1 | 1 |
| HBB | N-{(2r)-2-Hydroxy-2-[(8s,11s)-8-Isopropyl-6,9-Dioxo-2 Oxa-7,10-Diazabicyclo[11.2.2]Heptadeca-1(15),13,16 Trien-11-Yl]Ethyl}-N-Isopentylbenzenesulfonamide | 1 | 1 |
| HBH | 2-[(8s,11s)-11-{(1r)-1-Hydroxy-2 [Isopentyl(Phenylsulfonyl)Amino]Ethyl}-6,9-Dioxo-2 Oxa-7,10-Diazabicyclo[11.2.2]Heptadeca-1(15),13,16 Trien-8-Yl]Acetamide | 1 | 1 |
| HBO | 2,4-dihydroxy-7-(methyloxy)-2h-1,4-benzoxazin-3(4h)-one | 1 | 1 |
| HBU | 4-[hydroxy-[methyl-phosphinoyl]]-3-oxo-butanoicAcid | 1 | 1 |
| HCP | 1-[((1e)-{3-hydroxy-2-methyl-5-[(phosphonooxy)methyl]Pyridin-4-yl}methylene)amino]cyclopentylphosphonicAcid | 1 | 1 |
| HDI | 3-(4-hydroxyphenyl)-4,5-dihydro-5-isoxazole-aceticAcidMethylEster | 1 | 1 |
| HDS | 1-hexadecanosulfonicAcid | 1 | 1 |
| HDT | 4-[(4-imidazo[1,2-a]pyridin-3-ylpyrimidin-2-yl)amino]Benzenesulfonamide | 1 | 1 |
| HDU | N-[4-(2-methylimidazo[1,2-a]pyridin-3-yl)-2-Pyrimidinyl]acetamide | 1 | 1 |
| HDY | 1-(dimethylamino)-3-(4-{{4-(2-methylimidazo[1,2-a]Pyridin-3-yl)pyrimidin-2-yl]amino}phenoxy)propan-2-ol | 1 | 1 |
| HEL | (2s,5r,6r)-6-{[(6r)-6-(glycylamino)-7-oxido-7-Oxoheptanoyl]amino}-3,3-dimethyl-7-oxo-4-thia-1-Azabicyclo[3.2.0]heptane-2-carboxylate | 1 | 1 |
| HEY | (2e)-2-[({3-hydroxy-2-methyl-5-[(phosphonooxy)methyl]Pyridin-4-yl}methyl)amino]-5-phosphonopent-2-enoicAcid | 1 | 1 |
| HF1 | 4-(2-hydroxy-4-fluorophenylthio)-butylphosphonicAcid | 1 | 1 |
| HGU | N-hydroxyguanidine | 1 | 1 |
| HHG | (2r)-2-Hydroxy-3-(Phosphonooxy)PropylHexanoate | 1 | 1 |
| HI5 | 2-(butyryloxy)-1-{[(tetrahydroxyphosphoranyl)oxy]Methyl}ethylButyrate | 1 | 1 |
| HIO | N-hydroxy-n-isopropyloxamicAcid | 1 | 1 |
| HMD | 4-(5-amino-4-oxo-4h-pyrazol-3-yl)-2-bromo-4,5,6,7-Tetrahydro-3ah-pyrrolo[2,3-c]azepin-8-one | 1 | 1 |
| HMN | 2,4,6,7,8,9-hexahydroxy-5-methylcarboxamidoNonanoicAcid | 1 | 1 |
| HNA | 1,8-di-hydroxy-4-nitro-anthraquinone | 1 | 1 |
| HPI | Imidazole-pyrrole-hydroxypyrrolePolyamide | 1 | 1 |
| HPL | Heptylformamide | 1 | 1 |
| HPN | Heptanamide | 1 | 1 |
| HPO | 2-OxoheptylphosphonicAcid | 1 | 1 |
| HPP | HydroxyphenylPropionicAcid | 1 | 1 |
| HPT | 6-hydroxypropylthymine | 1 | 1 |
| HQQ | 5-methyl-5-(4-phenoxy-phenyl)-pyrimidine-2,4,6-trione | 1 | 1 |
| HRG | L-homoarginine | 1 | 1 |
| HSE | L-homoserine | 1 | 1 |
| HSO | Histidinol | 1 | 1 |
| HSP | 4-(2-hydroxyphenylsulfinyl)-butylphosphonicAcid | 1 | 1 |
| HTA | N-[3-(n'-hydroxycarboxamido)-2-(2-methylpropyl)-Propanoyl]-o-tyrosine-n-methylamide | 1 | 1 |
| HTC | 3-{[(4-Carboxy-2-Hydroxyaniline]Sulfonyl}Thiophene-2CarboxylicAcid | 1 | 1 |
| HTL | 2-acetyl-thiamineDiphosphate | 1 | 1 |
| HTP | 4,5,6-trihydroxy-7-hydroxymethyl-4,5,6,7-tetrahydro-1h-[1,2,3]triazolo[1,5-a]pyridin-8-ylium | 1 | 1 |
| HUX | 3-chloro-9-ethyl-6,7,8,9,10,11-hexahydro-7,11-Methanocycloocta[b]quinolin-12-amine | 1 | 1 |
| HWD | 2-amino-3-(2,4-dioxo-3,4-dihydro-2h-pyrimidin-1-yl)-PropionicAcid | 1 | 1 |
| HXP | 3,6-dihydroxy-xanthene-9-propionicAcid | 1 | 1 |
| HYA | 2,3,4,n-tetrahydroxy-butyrimidicAcid | 1 | 1 |
| I04 | N-{(3r,4r)-4-[4-(2-Fluoro-6-Hydroxy-3-Methoxy Benzoyl)-Benzoylamino]-Azepan-3-Yl}Isonicotinamide | 1 | 1 |
| I08 | (3r,4s)-N-(4-{Trans-2-[4-(2-Fluoro-6-Hydroxy-3 Methoxy-Benzoyl)-Phenyl]-Vinyl}-Azepan-3-Yl)Isonicotinamide | 1 | 1 |
| I1P | 2-[4-(n-(3-dimethylaminopropyl)sulphamoyl)anilino]- | 1 | 1 |
| I48 | N-{3-methyl-5-[2-(pyridin-4-ylamino)-ethoxy]-phenyl}-Benzenesulfonamide | 1 | 1 |
| I59 | Hydroxy(1-naphthyl)methylphosphonicAcid | 1 | 1 |
| I5P | Inositol-(1,3,4,5,6)-pentakisphosphate | 1 | 1 |
| IAB | Iso-acarbose | 1 | 1 |
| IAD | N-[1h-indol-3-yl-acetyl]asparticAcid | 1 | 1 |
| IAV | N-[1h-indol-3-yl-acetyl]valineAcid | 1 | 1 |
| IBA | 4-[(2r)-2-(aminomethyl)-2-(hydroxymethyl)-5-Oxopyrrolidin-1-yl]-3-[(1-ethylpropyl)amino]benzoicAcid | 1 | 1 |
| IBC | 3-(3-Hydroxy-7,8-Dihydro-6h-Cyclohepta[D]Isoxazol-4Yl)-L-Alanine | 1 | 1 |
| IBS | L-alpha-glycerophospho-d-myo-inositol-4,5-bis-phosphate | 1 | 1 |
| ICF | 1-Chloro-2,2,2-TrifluoroethylDifluoromethylEther | 1 | 1 |
| ID2 | 5-iododeoxyuridine | 1 | 1 |
| IDA | (2-carbamoylmethyl-5-propyl-octahydro-indol-7-yl)AceticAcid | 1 | 1 |
| IDM | Indoline | 1 | 1 |
| IDN | (e)-n-methyl-n-(1-methyl-1h-indol-3-ylmethyl)-3-(7-oxo-5,6,7,8-tetrahydro-[1,8]naphthyridin-3-yl)-acrylamide | 1 | 1 |
| IFM | 5-hydroxymethyl-3,4-dihydroxypiperidine | 1 | 1 |
| IFP | 2-trifluoromethyl-5-methylene-5h-pyrimidin-4-Ylideneamine | 1 | 1 |
| IH1 | 2-[2-(4-bromo-benzenesulfonyl)-ethyl]-1-3-dioxo-2,3,5,8-tetrahydro-1h-[1,2,4]triazolo[1,2-a]pyridazine-5-CarboxylicAcid(4-carbamimidoyl-cyclohexylmethyl)-amide | 1 | 1 |
| IH2 | 2-(2,2-diphenyl-ethyl)-7-methyl-1,3-dioxo-2,3,5,8-Tetrahydro-1h-[1,2,4]triazolo[1,2-a]pyridazine-5-CarboxylicAcid(4-carbamimidoyl-cyclohexylmethyl)-amide | 1 | 1 |
| IH4 | N-(R-Carboxy-Ethyl)-Alpha-(S)-(2-Phenylethyl) | 1 | 1 |
| IH5 | [4-(4-hydroxy-3-isopropylphenoxy)-3,5-dimethylphenyl]AceticAcid | 1 | 1 |
| IHB | 3-iodo-4-hydroxybenzoicAcid | 1 | 1 |
| IHG | N-isopropyl-n'-hydroxyguanidine | 1 | 1 |
| IKT | 3-(1-aminoethyl)nonanedioicAcid | 1 | 1 |
| ILO | N5-iminoethyl-l-ornithine | 1 | 1 |
| ILP | N-[o-phosphono-pyridoxyl]-isoleucine | 1 | 1 |
| IMB | [(isoquinolin-1-ylamino)-phosphono-methyl]-phosphonicAcid | 1 | 1 |
| IMG | 1,4-dideoxy-1,4-imino-1-(s)-(9-deazaguanin-9-yl)-d-Ribitol | 1 | 1 |
| IMK | 2-(Beta-D-Glucopyranosyl)-5-Methyl-1-Benzimidazole | 1 | 1 |
| IMZ | Cis-[4,5-bis-(4-bromophenyl)-2-(2-ethoxy-4-Methoxyphenyl)-4,5-dihydroimidazol-1-yl]-[4-(2-Hydroxyethyl)piperazin-1-yl]methanone | 1 | 1 |
| IN0 | N-{2-[trans-7-chloro-1-(2,2-dimethyl-propyl)-5-Naphthalen-1-yl-2-oxo-1,2,3,5-tetrahydro-benzo[e][1,4]oxazepin-3-yl]-acetyl}-asparticAcid | 1 | 1 |
| IN2 | N,n-dimethylcarbamoyl-alpha-azalysine | 1 | 1 |
| IN4 | +/-methyl4-(aminoiminomethyl)-beta-[3-Inh(aminoimino)phenyl]benzenePentanoate | 1 | 1 |
| IN7 | [4-(4-phenyl-piperidin-1-yl)-benzenesulfonylamino]-AceticAcid | 1 | 1 |
| IN8 | [2-(5-mercapto-[1,3,4]thiadiazol-2-ylcarbamoyl)-1-Phenyl-ethyl]-carbamicAcidBenzylEster | 1 | 1 |
| IN9 | 2-[3-(5-mercapto-[1,3,4]thiadiazol-2yl)-ureido]-n-Methyl-3-pentafluorophenyl-propionamide | 1 | 1 |
| INF | D-[(n-hydroxyamino)carbonyl]phenylalanine | 1 | 1 |
| INH | N-(r-carboxy-ethyl)-alpha-(s)-(2-phenylethyl)glycyl-l-Arginine-n-phenylamide | 1 | 1 |
| INJ | 5-(6-d-ribitylamino-2,4-dihydroxypyrimidin-5-yl)-1-Pentyl-phosphonicAcid | 1 | 1 |
| INL | 6-[n-(3-methoxy-phenyl)-3-(morpholin-4-ylmethyl)-2h-Thieno[3,2-e]-1,2-thiazine-1,1,-dioxide]-sulfonamide | 1 | 1 |
| INM | 4-amino-6-[n-(3-methoxylpropyl)-2h-thieno[3,2-e][1,2]Thiazine1,1-dioxide]-sulfonamide | 1 | 1 |
| INN | 3,n(d,l-[2-(hydroxyamino-carbonyl)methyl]-4-methylPentanoyl)l-3-(tert-butyl)glycyl-l-alanine | 1 | 1 |
| INQ | 6-[n-(3-hydroxy-phenyl)-3-(morpholin-4-ylmethyl)-2h-Thieno[3,2-e]-1,2-thiazine-1,1,-dioxide]-sulfonamide | 1 | 1 |
| INS | 1,2,3,4,5,6-hexahydroxy-cyclohexane | 1 | 1 |
| INU | N-(3-cyclopropyl(5,6,7,8,9,10-hexahydro-2-oxo-2h-Cycloocta[b]pyran-3-yl)methyl)phenylbenzensulfonamide | 1 | 1 |
| INV | 4-(aminosulfonyl)-n-[(4-fluorophenyl)methyl]-benzamide | 1 | 1 |
| INW | 4-(aminosulfonyl)-n-[(2,4-difluorophenyl)methyl]-Benzamide | 1 | 1 |
| INX | 2-(carboxymethoxy)-5-[(2s)-2-({(2s)-2-[(3-Carboxypropanoyl)amino]-3-phenylpropanoyl}amino)-3-Oxo-3-(pentylamino)propyl]benzoicAcid | 1 | 1 |
| INZ | 2-{4-[(2s)-2-[({[(1s)-1-carboxy-2-phenylethyl]amino}Carbonyl)amino]-3-oxo-3-(pentylamino)propyl]phenoxy}MalonicAcid | 1 | 1 |
| IOA | 4-(aminosulfonyl)-n-[(2,5-difluorophenyl)methyl]-Benzamide | 1 | 1 |
| IOC | 4-(aminosulfonyl)-n-[(2,3,4-trifluorophenyl)methyl]-Benzamide | 1 | 1 |
| IOE | 4-(aminosulfonyl)-n-[(2,4,6-trifluorophenyl)methyl]-Benzamide | 1 | 1 |
| IOF | 4-(aminosulfonyl)-n-[(3,4,5-trifluorophenyl)methyl]-Benzamide | 1 | 1 |
| IOL | 4-iodophenol | 1 | 1 |
| IOT | Arginine-n-methylcarbonylPhosphoricAcid5'-adenosineEster | 1 | 1 |
| IPC | 3-[Isopropyl(4-Methylbenzoyl)Amino]-5-Phenylthiophene2-Carboxylic Acid | 1 | 1 |
| IPO | Para-iodo-d-phenylalanineHydroxamicAcid | 1 | 1 |
| IPR | IsopentylPyrophosphate | 1 | 1 |
| IPU | S-isopropyl-isothiourea | 1 | 1 |
| IPZ | 2-isopropyl-3-methoxypyrazine | 1 | 1 |
| IQA | (5-oxo-5,6-dihydro-indolo[1,2-a]quinazolin-7-yl)-AceticAcid | 1 | 1 |
| IQB | N-[2-(4-bromocinnamylamino)ethyl]-5-isoquinolineSulfonamide | 1 | 1 |
| IQP | 1-(5-isoquinolinesulfonyl)-2-methylpiperazine | 1 | 1 |
| IQS | N-[2-(methylamino)ethyl]-5-isoquinolinesulfonamide | 1 | 1 |
| IQZ | (7as,12ar,12bs)-1,2,3,4,7a,12,12a,12b-Octahydroindolo[2,3-a]quinolizin-7(6h)-one | 1 | 1 |
| IRP | (1s)-1(9-deazahypoxanthin-9yl)1,4-dideoxy-1,4-imino-d-Ribitol-5-phosphate | 1 | 1 |
| IS2 | [(4-ethylphenyl)amino]carbonylphosphonicAcid | 1 | 1 |
| ISB | IsobutyricAcid | 1 | 1 |
| ISC | Isochorismate | 1 | 1 |
| ISZ | 4-(diazenylcarbonyl)pyridine | 1 | 1 |
| ITP | PhosphoricAcidMono-(2,3,4,6-tetrahydroxy-5-Phosphonooxy-cyclohexyl)Ester | 1 | 1 |
| IX1 | 5-(3-{3-[3-hydroxy-2-(methoxycarbonyl)phenoxy]Propenyl}phenyl)-4-(hydroxymethyl)isoxazole-3-CarboxylicAcid | 1 | 1 |
| IYG | N-alpha-acetyl-3,5-diiodotyrosylglycine | 1 | 1 |
| IYR | 3-iodo-tyrosine | 1 | 1 |
| IYT | N-alpha-acetyl-3,5-diiodotyrosyl-d-threonine | 1 | 1 |
| JAN | 6-[(s)-amino(4-chlorophenyl)(1-methyl-1h-imidazol-5-Yl)methyl]-4-(3-chlorophenyl)-1-methylquinolin-2(1h)-One | 1 | 1 |
| JE2 | (r)-n-(2-methylbenzyl)-3-[(2s,3s)-2-hydroxy-3-(3-Hydroxy-2-methylbenzoyl)amino-4-phenylbutanoyl]-5,5-Dimethyl-1,3-thiazolidine-4-carboxamide | 1 | 1 |
| JPC | 3-[(2,4-Dichlorobenzoyl)(Isopropyl)Amino]-5Phenylthiophene-2-CarboxylicAcid | 1 | 1 |
| KAM | N-[7-keto-8-aminopelargonicAcid]-[3-hydroxy-2-methyl-5-phosphonooxymethyl-pyridin-4-yl-methane] | 1 | 1 |
| KAR | 3"-(beta-chloroethyl)-2",4"-dioxo-3,5"-spiro-Oxazolidino-4-deacetoxy-vinblastine | 1 | 1 |
| KDG | 2-keto-3-deoxygluconate | 1 | 1 |
| KH1 | 5-(2-{1-[1-(4-ethyl-4-hydroxy-hexyloxy)-ethyl]-7a-Methyl-octahydro-inden-4-ylidene}-ethylidene)-4-Methylene-cyclohexane-1,3-diol | 1 | 1 |
| KHO | 4-(3,4-dihydroxy-5-hydroxymethyl-tetrahydro-furan-2-Yloxy)-tetrahydro-pyran-2,3,5-triol | 1 | 1 |
| KHP | 2-hydroxymethyl-5-(4-nitro-phenoxy)-tetrahydro-furan-3,4-diol | 1 | 1 |
| KMB | 2-amino-6-aminomethyl-8-phenylsulfanylmethyl-3h-Quinazolin-4-one | 1 | 1 |
| KMP | 3,5,7-trihydroxy-2-(4-hydroxyphenyl)-4h-chromen-4-one | 1 | 1 |
| KNI | (n-tert-butyl-thioproline)-(5-isoquinolyloxyacetyl-Methylthioalanine)-allophenylnorstatine | 1 | 1 |
| KOJ | 5-hydroxy-2-(hydroxymethyl)-4h-pyran-4-one | 1 | 1 |
| KPC | (2-[2-ketopropylthio]ethanesulfonate | 1 | 1 |
| KPL | Ketopantoate | 1 | 1 |
| KRI | (S)-2-Amino-4-[(2s,3r)-2,3,5-Trihydroxy-4-OxoPentyl]Mercapto-Butyric Acid | 1 | 1 |
| KSA | K-252a | 1 | 1 |
| L02 | 4-[3-(4-chlorophenyl)-1h-pyrazol-5-yl]piperidine | 1 | 1 |
| L03 | 3-(4-chlorophenyl)-5-(methylthio)-4h-1,2,4-triazole | 1 | 1 |
| L04 | 6-(4'-fluoro-biphenyl-4-yl)-4-(3-methyl-1-Phenylcarbamoyl-butylcarbamoyl)-2-[4-(1-oxo-1,3-Dihydro-isoindol-2-yl)-butyl]-hexanoicAcid | 1 | 1 |
| L08 | 1-acetyl-4-(4-{4-[(2-ethoxyphenyl)thio]-3-nitrophenyl}Pyridin-2-yl)piperazine | 1 | 1 |
| L10 | N-[(3z)-5-tert-butyl-2-phenyl-1,2-dihydro-3h-pyrazol-3-ylidene]-n'-(4-chlorophenyl)urea | 1 | 1 |
| L11 | N-[4-chloro-3-(pyridin-3-yloxymethyl)-phenyl]-3-fluoro- | 1 | 1 |
| L12 | 3-(2-pyridin-4-ylethyl)-1h-indole | 1 | 1 |
| L17 | L17 | 1 | 1 |
| L24 | [[[2-amino-5,6,7,8-tetrahydro-4-hydroxy-pyrido[2,3-d]Pyrimidin-6-yl]-ethyl]-phenyl]-carbonyl-glutamicAcid | 1 | 1 |
| L27 | {1-benzyl-3-[2-benzyl-3-oxo-4-(1-oxo-1,2,3,4-Tetrahydro-Isoquinolin-4-yl)-2,3-dihydro-1h-pyrrol-2-Yl]-2-Hydroxy-propyl}-carbamicAcidTetrahydro-furan-3-ylEster | 1 | 1 |
| L37 | [[n'-(2,5-diamino-6-hydroxy-pyrimidin-4-yl)-ureayl]-Phen-4-yl]-carbonyl-glutamicAcid | 1 | 1 |
| L47 | 3-[(5s)-1-Acetyl-3-(2-Chlorophenyl)-4,5-Dihydro-1hPyrazol-5-Yl]Phenol | 1 | 1 |
| L75 | N-[2(r)-hydroxy-1(s)-indanyl]-2(r)-phenylmethyl-4(s)-Hydroxy-5-[4-[2-benzofuranylmethyl]-2(s)-[tert-Butylaminocarbonyl]-piperazinyl]-pentaneamide | 1 | 1 |
| L86 | (11s)-11-benzyl-6-chloro-1,2,10,11,12,13,14,15,16,17,18,19-dodecahydro-5,9-methano-2,5,8,10,13,17-Benzohexaazacyclohenicosine-3,24-dione | 1 | 1 |
| LAD | Adenosine-5'-[lysyl-phosphate] | 1 | 1 |
| LAE | 3-oxo-octanoicAcid(2-oxo-tetrahydro-furan-3-yl)-amide | 1 | 1 |
| LAG | Maltosyl-alpha(1,4)-(z,3s,4s,5r,6r)-3,4,5-trihydroxy-6-hydroxymethyl-piperidin-2-oneOxime | 1 | 1 |
| LAK | Beta-d-galactopyranosyl-1-6-beta-d-glucopyranose | 1 | 1 |
| LDP | L-dopamine | 1 | 1 |
| LDT | Lidorestat | 1 | 1 |
| LGC | (3s,4r,5r,6s)-3,4,5-trihydroxy-6-(hydroxymethyl)Tetrahydro-2h-pyran-2-one | 1 | 1 |
| LGZ | CarbamicAcid1-{5-benzyl-5-[2-hydroxy-4-phenyl-3-(tetrahydro-furan-3-yloxycarbonylamino)-butyl]-4-oxo-4,5-dihydro-1h-pyrrol-3-yl}-Indan-2-ylEster | 1 | 1 |
| LHY | L-[(n-hydroxyamino)carbonyl]phenylalanine | 1 | 1 |
| LI6 | 3,4-Dihydroxy-1-Methylquinolin-2(1h)-One | 1 | 1 |
| LI7 | (3e)-3-[(4-Hydroxyphenyl)Imino]-1h-Indol-2(3h)-One | 1 | 1 |
| LI9 | 4-(4-Fluorophenyl)-1-Methyl-5-(2-{[(1s)-1 Phenylethyl]Amino}Pyrimidin-4-Yl)-2-Piperidin-4-Yl-1 2-Dihydro-3h-Pyrazol-3-One | 1 | 1 |
| LIG | 3-pyridin-4-yl-2,4-dihydro-indeno[1,2-.c.]pyrazole | 1 | 1 |
| LIH | 6-([5-quinolylamino]methyl)-2,4-diamino-5-Methylpyrido[2,3-d]pyrimidine | 1 | 1 |
| LII | (z)-6-(2-[2,5-dimethoxyphenyl]ethen-1-yl)-2,4-diamino-5-methylpyrido[2,3-d]pyrimidine | 1 | 1 |
| LIP | L-myo-inositol-1-phosphate | 1 | 1 |
| LKA | 5-thio-a/b-d-mannopyranosylamine | 1 | 1 |
| LKS | N-(1-iminio-2-phenylethyl)-5-thiohexopyranosylamineBromide | 1 | 1 |
| LM2 | 4'-o-methyl-maltosyl-alpha(1,4)-(z,3s,4s,5r,6r)-3,4,5-trihydroxy-6-hydroxymethyl-piperidin-2-one | 1 | 1 |
| LMT | Dodecyl-beta-d-maltoside | 1 | 1 |
| LMZ | 5-nitroso-6-ribityl-amino-2,4(1h,3h)-pyrimidinedione | 1 | 1 |
| LNO | L-leucyl-hydroxylamine | 1 | 1 |
| LO1 | [[4-(aminomethyl)phenyl]amino]oxo-aceticAcid, | 1 | 1 |
| LPC | [1-myristoyl-glycerol-3-yl]phosphonylcholine | 1 | 1 |
| LPE | 1-o-octadecyl-sn-glycero-3-phosphocholine | 1 | 1 |
| LS1 | N-methyl-4-{[(2-oxo-1,2-dihydro-3h-indol-3-ylidene)Methyl]amino}benzenesulfonamide | 1 | 1 |
| LS2 | N-methyl-{4-[2-(7-oxo-6,7-dihydro-8h-[1,3]thiazolo[5,4-e]indol-8-ylidene)hydrazino]phenyl}methanesulfonamide | 1 | 1 |
| LS3 | 3-{[(2,2-dioxido-1,3-dihydro-2-benzothien-5-yl)amino]Methylene}-5-(1,3-oxazol-5-yl)-1,3-dihydro-2h-indol-2-One | 1 | 1 |
| LS4 | 4-{[(2-oxo-1,2-dihydro-3h-indol-3-ylidene)methyl]Amino}-n-(1,3-thiazol-2-yl)benzenesulfonamide | 1 | 1 |
| LS5 | 3-{[4-([amino(imino)methyl]aminosulfonyl)anilino]Methylene}-2-oxo-2,3-dihydro-1h-indole | 1 | 1 |
| LTL | 6-hydroxy-6-methyl-heptan-3-one | 1 | 1 |
| LTN | L-tryptophanamide | 1 | 1 |
| LXP | L-xylitol5-phosphate | 1 | 1 |
| LY3 | 2-{4-[2-(2-amino-4-oxo-4,7-dihydro-3h-pyrrolo[2,3-d]Pyrimidin-5-yl)-ethyl]-benzoylamino}-4-(2h-tetrazol-5-Yl)-butyricAcid | 1 | 1 |
| LY4 | (9r)-9-[(dimethylamino)methyl]-6,7,10,11-tetrahydro-9h,18h-5,21:12,17-dimethenodibenzo[e,k]pyrrolo[3,4-h][1,4,13]oxadiazacyclohexadecine-18,20-dione | 1 | 1 |
| LYL | 2-allyl-6-methyl-phenol | 1 | 1 |
| M1C | (3s)-3-amino-1-(cyclopropylamino)heptane-2,2-diol | 1 | 1 |
| M1P | D-mannose1-phosphate | 1 | 1 |
| M2C | (2s)-2-amino-4-(methylsulfanyl)-1-pyridin-2-ylbutane-1,1-diol | 1 | 1 |
| M2P | D-Mannitol-1,6-Diphosphate | 1 | 1 |
| M3C | (2s)-2-amino-4-(methylsulfanyl)-1-(1,3-thiazol-2-yl)Butane-1,1-diol | 1 | 1 |
| M77 | 5-(1,4-diazepan-1-sulfonyl)isoquinoline | 1 | 1 |
| M7P | D-glycero-d-mannopyranose-7-phosphate | 1 | 1 |
| MAB | Mannobiose | 1 | 1 |
| MAQ | 2-amino-8-methylquinazolin-4(3h)-one | 1 | 1 |
| MAU | N-methylKirromycin | 1 | 1 |
| MBP | 1-[(2-amino-6,9-dihydro-1h-purin-6-yl)oxy]-3-methyl-2-Butanol | 1 | 1 |
| MBS | R-2-{[4'-methoxy-(1,1'-biphenyl)-4-yl]-sulfonyl}-amino-6-methoxy-hex-4-ynoicAcid | 1 | 1 |
| MBV | MesobiliverdinIvAlpha | 1 | 1 |
| MCI | (2-mercaptomethyl-4-phenyl-butyrylimino)-(5-tetrazol-1-ylmethyl-thiophen-2-yl)-aceticAcid | 1 | 1 |
| MCR | SulfanylaceticAcid | 1 | 1 |
| MCT | 4-methylcatechol | 1 | 1 |
| MD2 | N,n'-bis(2,3-butadienyl)-1,4-butane-diamine | 1 | 1 |
| ME2 | 1-ethoxy-2-(2-methoxyethoxy)ethane | 1 | 1 |
| MEV | (r)-mevalonate | 1 | 1 |
| MGA | Beta-methyl-d-galactoside | 1 | 1 |
| MGB | MethylglyoxalBis-(guanylhydrazone) | 1 | 1 |
| MHB | 2-((3'-methyl-4'-hydroxyphenyl)azo)benzoicAcid | 1 | 1 |
| MHF | 5,10-methenyl-6,7,8-trihydrofolicAcid | 1 | 1 |
| MHN | 6-methylheptan-1-ol | 1 | 1 |
| MIC | Alpha-methylisocitricAcid | 1 | 1 |
| MIL | Milrinone | 1 | 1 |
| MIM | [cyclohexylethyl]-[[[[4-[2-methyl-1-imidazolyl-butyl]Phenyl]acetyl]-seryl]-lysinyl]-amine | 1 | 1 |
| MIN | Methyl-phe-pro-amino-cyclohexylglycine | 1 | 1 |
| MLC | Malonyl-coenzymeA | 1 | 1 |
| MLM | 3-amino-3-oxopropanoicAcid | 1 | 1 |
| MMN | 5-deoxy-5-{[(1s)-1-hydroxyethyl]amino}-d-glucitol | 1 | 1 |
| MMQ | MercaptomethylPhosphonate | 1 | 1 |
| MNA | 2-o-methyl-5-n-acetyl-alpha-d-NeuraminicAcid | 1 | 1 |
| MNS | 5-(dimethylamino)-1-naphthalenesulfonamide | 1 | 1 |
| MNX | 1,8-di-hydroxy-4-nitro-xanthen-9-one | 1 | 1 |
| MNY | 5,8-di-amino-1,4-dihydroxy-anthraquinone | 1 | 1 |
| MOB | 2-((3',5'-dimethoxy-4'-hydroxyphenyl)azo)benzoicAcid | 1 | 1 |
| MOD | PhosphoricAcid2-amino-4-methylsulfanyl-butylEster5-(6-amino-purin-9-yl)-3,4-dihydroxy-tetrahydro-furan-2-ylmethylEster | 1 | 1 |
| MP5 | (4-amino-2-methylpyrimidin-5-yl)methylDihydrogenPhosphate | 1 | 1 |
| MPB | 4-hydroxy-benzoicAcidMethylEster | 1 | 1 |
| MPI | Imidazo[1,2-a]pyridine | 1 | 1 |
| MPM | (1r)-1-[((1e)-{3-hydroxy-2-methyl-5-[(phosphonooxy)Methyl]pyridin-4-yl}methylene)amino]-1-MethylpropylphosphonicAcid | 1 | 1 |
| MPP | 3-(3,4-dimethoxyphenyl)propionicAcid | 1 | 1 |
| MQ0 | (3-aminomethyl-cinnolin-4-yl)-(3,3-diphenyl-Allylidene)-amine | 1 | 1 |
| MQ1 | 5-(4-methoxyphenoxy)-2,4-quinazolinediamine | 1 | 1 |
| MQU | 7-[2-methoxy-1-(methoxymethyl)ethyl]-7h-pyrrolo[3,2-f]Quinazoline-1,3-diamine | 1 | 1 |
| MRY | Meso-erythritol | 1 | 1 |
| MS2 | 2,2-dichloro-1-methanesulfinyl-3-methyl-CyclopropanecarboxylicAcid[1-(4-bromo-phenyl)-ethyl]-amide | 1 | 1 |
| MS3 | N,n-[2,5-o-dibenzyl-glucaryl]-di-[valinyl-Aminomethanyl-pyridine] | 1 | 1 |
| MSC | 2,5-dibenzyloxy-3,4-dihydroxy-hexanedioicAcid2-Chloro-6-fluoro-benzylamide(2-hydroxy-indan-1-Yl)-Amide | 1 | 1 |
| MSP | 5'-o-[(l-methionyl)-sulphamoyl]adenosine | 1 | 1 |
| MST | 2-t-butylamino-4-ethylamino-6-methylthio-s-triazine | 1 | 1 |
| MTB | 2-((3'-tertbutyl-4'-hydroxyphenyl)azo)benzoicAcid | 1 | 1 |
| MTG | [methylthio]acetate | 1 | 1 |
| MTI | 3,4-dihydroxy-2-[(methylsulfanyl)methyl]-5-(4-oxo-4,5-Dihydro-3h-pyrrolo[3,2-d]pyrimidin-7-yl)pyrrolidinium | 1 | 1 |
| MTL | D-mannitol | 1 | 1 |
| MTP | 2-hydroxymethyl-5-(6-methylsulfanyl-purin-9-yl)-Tetrahydro-furan-3,4-diol | 1 | 1 |
| MTS | (4s-trans)-4-(methylamino)-5,6-dihydro-6-methyl-4h-Thieno(2,3-b)thiopyran-2-sulfonamide-7,7-dioxide | 1 | 1 |
| MUA | 9-methylUricAcid | 1 | 1 |
| MUR | MuramicAcid | 1 | 1 |
| MUS | 4-methyl-2-oxo-2h-chromen-7-yl5-(acetylamino)-3,5-Dideoxy-l-erythro-non-2-ulopyranosidonicAcid | 1 | 1 |
| MXA | 6-(2,5-dimethoxy-benzyl)-5-methyl-pyrido[2,3-d]Pyrimidine-2,4-diamine | 1 | 1 |
| MYG | Glucosaminyl-(alpha-6)-d-myo-inositol | 1 | 1 |
| MYT | Metyrapone | 1 | 1 |
| MZM | 5-acetamido-4-methyl-1,3,4-thiadiazole-2-sulfonamide | 1 | 1 |
| MZP | 4-carbamoyl-1-beta-d-ribofuranosyl-imidazolium-5-olate-5'-phosphate | 1 | 1 |
| N1T | 1'-deazo-thiaminDiphosphate | 1 | 1 |
| N25 | 1,3-bis-(4-methoxy-benzenesulfonyl)-5,5-dimethyl-Hexahydro-pyrimidine-2-carboxylicAcidHydroxyamide | 1 | 1 |
| N2M | 5-Amino-Naphtalene-2-Monosulfonate | 1 | 1 |
| N5B | N-(5-cyclopropyl-1h-pyrazol-3-yl)benzamide | 1 | 1 |
| NAB | 2-((4'-hydroxynaphthyl)-azo)benzoicAcid | 1 | 1 |
| NAE | NicotinamideAdenineDinucleotideAcetoneAdduct | 1 | 1 |
| NAQ | NicotinamideAdenineDinucleotide3-pentanoneAdduct | 1 | 1 |
| NAT | Ethyl4-(3-hydroxyphenyl)-6-methyl-2-thioxo-1,2,3,4-Tetrahydropyrimidine-5-carboxylate | 1 | 1 |
| NAX | Beta-6-hydroxy-1,4,5,6-tetrhydronicotinamideAdenineDinucleotide | 1 | 1 |
| NAY | 6,7,8,9-tetrahydroxy-5-methylcarboxamido-2-oxononanoicAcid | 1 | 1 |
| NBB | N-butyl-benzenesulfonamide | 1 | 1 |
| NBP | Nicotinamide8-bromo-adenineDinucleotidePhosphate | 1 | 1 |
| NBZ | Nitrobenzene | 1 | 1 |
| NCM | Norcamphor | 1 | 1 |
| NCN | NicotinateMononucleotide | 1 | 1 |
| NDC | NicotinamideAdenineDinucleotideCyclohexanone | 1 | 1 |
| NDG | 2-(acetylamino)-2-deoxy-a-d-glucopyranose | 1 | 1 |
| NDH | (1r,2s)-cis1,2Dihydroxy-1,2-dihydronaphthalene | 1 | 1 |
| NDO | Nicotinamide-(6-deamino-6-hydroxy-adenine)-DinucleotidePhosphate | 1 | 1 |
| NDS | EthylDimethylAmmonioPropaneSulfonate | 1 | 1 |
| NEA | 5'-deoxy-5'-[2-(amino)ethylthio]adenosine | 1 | 1 |
| NEO | Neopterin | 1 | 1 |
| NES | 2-(2-hydroxy-1,1-dihydroxymethyl-ethylamino)-EthanesulfonicAcid | 1 | 1 |
| NEV | Nevirapine | 1 | 1 |
| NFL | 2-{[3-(trifluoromethyl)phenyl]amino}nicotinicAcid | 1 | 1 |
| NFZ | Nitrofurazone | 1 | 1 |
| NGL | Acetylgalactosamine-4-sulfate | 1 | 1 |
| NGV | Methyl5,7-dihydroxy-2-methyl-4,6,11-trioxo-3,4,6,11-Tetrahydrotetracene-1-carboxylate | 1 | 1 |
| NH1 | 3-(4-amino-2-tert-butyl-5-methyl-phenylsulfanyl)-6-Cyclopentyl-4-hydroxy-6-[2-(4-hydroxy-phenyl)-ethyl]-5,6-dihydro-pyran-2-one | 1 | 1 |
| NHB | N-hydroxy-4-(methyl{[5-(2-pyridinyl)-2-thienyl]Sulfonyl}amino)benzamide | 1 | 1 |
| NHD | NicotinamidePurin-6-ol-dinucleotide | 1 | 1 |
| NHP | 4-(2-aminophenylthio)-butylphosphonicAcid | 1 | 1 |
| NHR | 2-{4-[2-(2-amino-4-hydroxy-quinazolin-6-yl)-1-carboxy-Ethyl]-benzoylamino}-pentanedioicAcid | 1 | 1 |
| NHS | 10-formyl-5,8,10-trideazafolicAcid | 1 | 1 |
| NIC | NitroisocitricAcid | 1 | 1 |
| NID | 4-nitro-inden-1-one | 1 | 1 |
| NIR | 3-(aminocarbonyl)-1-[(3r,4s,5r)-3,4-dihydroxy-5-Methyltetrahydro-2-furanyl]pyridinium | 1 | 1 |
| NIU | 6,7,8,9-tetrahydro-4-hydroxy-3-(1-phenylpropyl)Cyclohepta[b]pyran-2-one | 1 | 1 |
| NLA | Naphthalen-1-yl-aceticAcid | 1 | 1 |
| NLE | Norleucine | 1 | 1 |
| NLP | (1-amino-pentyl)-phosphonicAcid | 1 | 1 |
| NLQ | N~2~-acetyl-l-glutamine | 1 | 1 |
| NM1 | 3-(7-benzyl-4,5-dihydroxy-1,1-dioxo-3,6-bis-Phenoxymethyl-1l6-[1,2,7]thiadiazepan-2-ylmethyl)-n-Methyl-benzamide | 1 | 1 |
| NMB | 2,7-dibenzyl-1,1-dioxo-3,6-bis-phenoxymethyl-[1,2,7]Thiadiazepane-4,5-diol | 1 | 1 |
| NOM | 7-hydroxy-5-methyl-3,3a,5,11b-tetrahydro-1,4-dioxa-Cyclopenta[a]anthracene-2,6,11-trione | 1 | 1 |
| NON | MethylNonanoate(ester) | 1 | 1 |
| NPG | N-succinylPhenylglycine | 1 | 1 |
| NPO | P-nitrophenol | 1 | 1 |
| NST | 3-{[(3-Nitroaniline]Sulfonyl}Thiophene-2-CarboxylicAcid | 1 | 1 |
| NTC | 2-hydroxy-2-nitromethylSuccinicAcid | 1 | 1 |
| NTD | 2-{hydroxy[2-nitro-4-(trifluoromethyl)phenyl]Methylene}cyclohexane-1,3-dione | 1 | 1 |
| NTM | QuinolinicAcid | 1 | 1 |
| NVI | 1-vinylimidazole | 1 | 1 |
| NW1 | 6-cyclohexylmethyloxy-5-nitroso-pyrimidine-2,4-diamine | 1 | 1 |
| NXA | N-carboxyalanine | 1 | 1 |
| NYL | N-allyl-aniline | 1 | 1 |
| NYM | 3'-deoxy-3'-aminothymidineMonophosphate | 1 | 1 |
| NZQ | 5,6-dihydroxy-nadp | 1 | 1 |
| OAD | 2'-o-acetylAdenosine-5-diphosphoribose | 1 | 1 |
| OAI | 6-(oxalyl-amino)-1h-indole-5-carboxylicAcid | 1 | 1 |
| OAL | (1,8-dihydroxy-9,10-dioxo-9,10-dihydro-anthracen-2-yl)-aceticAcid | 1 | 1 |
| OAP | 4-(s)-[(1-oxo-7-phenylheptyl)amino]-5-[4-(phenylmethyl)phenylthio]pentanoicAcid | 1 | 1 |
| OCH | Quinolin-2(1h)-One | 1 | 1 |
| OCV | N6-[(1r)-2-{[(1r)-1-carboxy-2-methylpropyl]oxy}-1-(mercaptomethyl)-2-oxoethyl]-6-oxo-d-lysine | 1 | 1 |
| ODP | 4-oxo-nicotinamide-adenineDinucleotidePhosphate | 1 | 1 |
| OIN | (1r,5s)-8-methyl-8-azabicyclo[3.2.1]oct-3-yl(2r)-3-Hydroxy-2-phenylpropanoate | 1 | 1 |
| OIR | N-(3-phenyl-2-sulfanylpropanoyl)phenylalanylalanine | 1 | 1 |
| OLN | (s)-2-acetamido-5-ureidopentanoicAcid | 1 | 1 |
| OMP | Orotidine-5'-monophosphate | 1 | 1 |
| OPB | 4-butyl-1-(4-hydroxyphenyl)-2-phenylpyrazolidine-3,5-Dione | 1 | 1 |
| OPE | PhosphoricAcidMono-(2-amino-ethyl)Ester | 1 | 1 |
| OSC | (2r,3as,6r,7as)-n-(2-{1-[amino(imino)methyl]-2,5-Dihydro-1h-pyrrol-3-yl}ethyl)-6-hydroxy-1-{n-[(2s)-2-Hydroxy-3-phenylpropanoyl]phenylalanyl}octahydro-1h-Indole-2-carboxamide | 1 | 1 |
| OSU | N-octanoylsucrose | 1 | 1 |
| OTA | 2-(oxalyl-amino)-4,5,6,7-tetrahydro-thieno[2,3-c]Pyridine-3-carboxylicAcid | 1 | 1 |
| OTG | Ortho-toluoylglucosamine | 1 | 1 |
| OX2 | 2-(Beta-D-Glucopyranosyl)-5-Methyl-1,3,4-Oxadiazole | 1 | 1 |
| OXG | 8-oxoguanine | 1 | 1 |
| OXQ | 4-hydroxy-1,2,5-oxadiazole-3-carboxylicAcid | 1 | 1 |
| OXZ | Tetrahydrooxazine | 1 | 1 |
| P10 | [1-(3-hydroxy-2-oxo-1-phenethyl-propylcarbamoyl)2-Phenyl-ethyl]-carbamicAcidPyridin-4-ylmethylEster | 1 | 1 |
| P1C | Deacetoxycephalosporin-c | 1 | 1 |
| P27 | {[2-(1h-1,2,3-benzotriazol-1-yl)-2-(3,4-Difluorophenyl)propane-1,3-diyl]bis[4,1-Phenylene(difluoromethylene)]}bis(phosphonicAcid) | 1 | 1 |
| P28 | 3',5'-dinitro-n-acetyl-l-thyronine | 1 | 1 |
| P2S | (2s)-2-amino-4-[[(2r)-2-carboxybutyl](phosphono)Sulfonimidoyl]butanoicAcid | 1 | 1 |
| P3M | 1-3SugarRingOfPentamannosyl6-phosphate | 1 | 1 |
| P90 | {4-[(2s,4e)-2-(1,3-benzothiazol-2-yl)-2-(1h-1,2,3-Benzotriazol-1-yl)-5-phenylpent-4-enyl]phenyl}(difluoro)methylphosphonicAcid | 1 | 1 |
| PAN | 5-phospho-d-arabinohydroxamicAcid | 1 | 1 |
| PAO | N-(phosphonoacetyl)-l-ornithine | 1 | 1 |
| PAU | PantothenoicAcid | 1 | 1 |
| PAV | (2r,4s)-2-methyl-2,3,3,4-tetrahydroxytetrahydrofuran | 1 | 1 |
| PBE | 1,1-dimethyl-prolinium | 1 | 1 |
| PBZ | P-aminoBenzamidine | 1 | 1 |
| PCO | 2,4-dihydroxy-n-[2-(2-mercapto-vinylcarbamoyl)-ethyl]-3,3-dimethyl-butyramide | 1 | 1 |
| PCR | P-cresol | 1 | 1 |
| PCT | Phosphonoacetamide | 1 | 1 |
| PCV | 5-amino-3-hydroxy-2-(2-oxo-azetidin-1-yl)-pentanoicAcid | 1 | 1 |
| PCX | DeoxyguanidinoproclavaminicAcid | 1 | 1 |
| PD1 | 1-tert-butyl-3-[6-(3,5-dimethoxy-phenyl)-2-(4-Diethylamino-butylamino)-pyrido[2,3-d]pyrimidin-7-yl]-Urea | 1 | 1 |
| PD8 | PhosphorylatedDihydropteroate | 1 | 1 |
| PDD | N-(5'-phosphopyridoxyl)-d-alanine | 1 | 1 |
| PE2 | 9-(4-hydroxy-3-(hydroxymethyl)but-1-yl)guanine | 1 | 1 |
| PEI | PentanoicAcid | 1 | 1 |
| PF3 | (2r)-3-({[4-[(pyridin-4-yl)phenyl]-thien-2-yl}Carboxamido)(phenyl)propanoicAcid | 1 | 1 |
| PFA | [4-(4-hydroxy-3-isopropyl-phenoxy)-3,5-dimethyl-Phenyl]-6-azauracil | 1 | 1 |
| PFB | 2,3,4,5,6-pentafluorobenzylAlcohol | 1 | 1 |
| PFD | 5-(2-Ethoxyethyl)-5-[4-(4-Fluorophenoxy)Phenoxy]Pyrimidine-2,4,6(1h,3h,5h)-Trione | 1 | 1 |
| PFL | 2,6-bis(1-methylethyl)phenol | 1 | 1 |
| PFM | 1-amino-7-(2-methyl-3-oxido-5-((phosphonoxy)methyl)-4-Pyridoxal-5-oxo-6-heptenate | 1 | 1 |
| PFP | 2-[5,6-Bis-(4-Methoxy-Phenyl)-Furo[2,3-DPyrimidin-4-Ylamino]-Ethanol | 1 | 1 |
| PFQ | 2-[(5,6-Diphenylfuro[2,3-D]Pyrimidin-4-Yl)Amino]Ethanol | 1 | 1 |
| PG2 | ProstaglandinD2 | 1 | 1 |
| PG3 | Guanidine-3-propanol | 1 | 1 |
| PGJ | 2-(Ethoxymethyl)-4-(4-Fluorophenyl)-3-[2-(2 Hydroxyphenoxy)Pyrimidin-4-Yl]Isoxazol-5(2h)-One | 1 | 1 |
| PGM | 1-myristoyl-2-hydroxy-sn-glycero-3-[phospho-rac-(1-Glycerol)] | 1 | 1 |
| PH3 | N-(chlorophenyl)-n'-hydroxyguanidine | 1 | 1 |
| PHH | 4,5,6,7-tetrachloro-phthalide | 1 | 1 |
| PHI | Iodo-phenylalanine | 1 | 1 |
| PHN | 1,10-phenanthroline | 1 | 1 |
| PHT | PhthalicAcid | 1 | 1 |
| PI1 | 1-[2-(8-carbamoylmethyl-6,9-dioxo-2-oxa-7,10-diaza-Bicyclo[11.2.2]Heptadeca-1(16),13(17),14-trien-11-yl)-2-hydroxy-ethyl]-pyrrolidine-2-carboxylicAcid[1-(1-carbamoyl-2-methyl-propylcarbamoyl)-2-methyl-Butyl]-amide | 1 | 1 |
| PI2 | 2-{11-[1-hydroxy-2-(3-methyl-butylamino)-ethyl]-6,9-Dioxo-2-oxa-7,10-diaza-bicyclo[11.2.2]heptadeca-1(16),13(17),14-trien-8-yl}-acetamide | 1 | 1 |
| PI3 | 11-[1-hydroxy-2-(3-methyl-butylamino)-ethyl]-8-Isopropyl-2-oxa-7,10-Diaza-bicyclo[11.2.2]heptadeca-1(16),13(17),14-triene-6,9-dione | 1 | 1 |
| PI4 | 1-[2-(8-carbamoylmethyl-6,9-dioxo-2-oxa-7,10-diaza-Bicyclo[11.2.2]heptadeca-1(16),13(17),14-trien-11-yl)-2-hydroxy-ethyl]-piperidine-2-carboxylicAcidTert-Butylamide | 1 | 1 |
| PI5 | N-[3-(8-sec-butyl-7,10-dioxo-2-oxa-6,9-diaza-Bicyclo[11.2.2]heptadeca-1(16),13(17),14-Trien-11-Ylamino)-2-hydroxy-1-(4-hydroxy-benzyl)-propyl]-3-Methyl-2-(2-oxo-pyrrolidin-1-yl)-butyramide | 1 | 1 |
| PI6 | [1-benzyl-3-(8-sec-butyl-7,10-dioxo-2-oxa-6,9-diaza-Bicyclo[11.2.2]Heptadeca-1(16),13(17),14-trien-11-Ylamino)-2-hydroxy-propyl]-carbamicAcidTert-butylEster | 1 | 1 |
| PI7 | N-[3-(8-sec-butyl-7,10-dioxo-2-oxa-6,9-diaza-Bicyclo[11.2.2]Heptadeca-1(16),13(17),14-trien-11-Yamino)-2-hydroxy-1-(4-hydroxy-benzyl)-propyl]-3-Methyl-2-propionylamino-butyramide | 1 | 1 |
| PI8 | N-13-[(10s,13s)-9,12-dioxo-10-(2-butyl)-2-oxa-8,11-Diazabicyclo[13.2.2]Nonadeca-15,17,18-triene](2r)-Benzyl-(4s)-hydroxy-5-aminopentanoic(1r)-hydroxy-(2s)-indaneamide | 1 | 1 |
| PI9 | (10s,13s,1'r)-13-[1'-hydroxy-2'-(n-p-Aminobenzenesulfonyl-1''-amino-3''-methylbutyl)ethyl]-8,11-dioxo-10-isopropyl-2-oxa-9,12-diazabicyclo[13.2.2]nonadeca-15,17,18-triene | 1 | 1 |
| PIC | 6-(difluoro-phosphono-methyl)-naphthalene-2-carboxylicAcid | 1 | 1 |
| PID | Peridinin | 1 | 1 |
| PIR | 2-(4-amino-phenyl)-5-hydroxymethyl-pyrrolidine-3,4-diol | 1 | 1 |
| PKF | Carbobenzoxy-pro-lys-phe-y(po2)-ala-pro-ome | 1 | 1 |
| PLR | (5-hydroxy-4,6-dimethylpyridin-3-yl)methylDihydrogenPhosphate | 1 | 1 |
| PLT | [3-hydroxy-2-methyl-5-phosphonooxymethyl-pyridin-4-Ylmethyl]-l-tryptophane | 1 | 1 |
| PM1 | [2-amino-6-(2,6-difluoro-benzoyl)-imidazo[1,2-a]Pyridin-3-yl]-phenyl-methanone | 1 | 1 |
| PM2 | 1-[3-(1-{[5-(2-Phenylethyl)Pyridin-3-Yl Carbonyl}Piperidin-4-Yl)Phenyl]Methanamine | 1 | 1 |
| PMD | [n-(2,4-diaminopteridin-6-yl)-methyl]-dibenz[b,f]Azepine | 1 | 1 |
| PMH | (5-hydroxy-4-{[(3-hydroxyisoxazol-4-yl)amino]methyl}-6-methylpyridin-3-yl)methylDihydrogenPhosphate | 1 | 1 |
| PML | PimelicAcid | 1 | 1 |
| PMM | Pterin-6-yl-methyl-monophosphate | 1 | 1 |
| PMO | N1-(5'-phospho-alpha-ribosyl)-5-methoxybenzimidazole | 1 | 1 |
| PMS | BenzylsulfinicAcid | 1 | 1 |
| PMT | PhosphoricAcidMono-[3-(3-{[5-(4-amino-2-oxo-2h-Pyrimidin-1-yl)-3,4-Dihydroxy-tetrahydro-furan-2-Ylmethoxy]-hydroxy-phosphoryloxy}-3-oxo-Propylcarbamoyl)-3-hydroxy-2,2-Dimethyl-propyl]Ester | 1 | 1 |
| POA | Phosphonoacetaldehyde | 1 | 1 |
| POB | 2-[(1-amino-ethyl)-phosphate-phosphinoyloxy]-butyricAcid | 1 | 1 |
| PP1 | 1-ter-butyl-3-p-tolyl-1h-pyrazolo[3,4-d]pyrimidin-4-Ylamine | 1 | 1 |
| PP2 | 1-tert-butyl-3-(4-chloro-phenyl)-1h-pyrazolo[3,4-d]Pyrimidin-4-ylamine | 1 | 1 |
| PP4 | Methyl(2s)-[1-((n-formyl)-l-valyl)amino-2-(2-Naphthyl)ethyl)hydroxyphosphinyloxy]-3-phenylPropanoate | 1 | 1 |
| PP5 | Methyl(2s)-[1-((n-(naphthaleneacetyl))-l-valyl)Aminomethyl)hydroxyphosphinyloxy]-3-phenylPropanoate | 1 | 1 |
| PP7 | MethylCyclo[(2s)-2-[[(1r)-1-(n-(l-n-(3-Methylbutanoyl)valyl-l-aspartyl)amino)-3-methylbutyl]Hydroxyphosphinyloxy]-3-(3-aminomethyl)phenylpropanoate | 1 | 1 |
| PP8 | 2-[(1r)-1-(n-(3-methylbutanoyl)-l-valyl-l-asparaginyl)-amino)-3-methylbutyl]hydroxyphosphinyloxy]-3-PhenylpropanoicAcidMethylester | 1 | 1 |
| PPR | Phosphonopyruvate | 1 | 1 |
| PPS | 3'-phosphate-adenosine-5'-phosphateSulfate | 1 | 1 |
| PPT | 3-(p-tolyl)propionicAcid | 1 | 1 |
| PPX | [phenylalaninyl-prolinyl]-[2-(pyridin-4-ylamino)-Ethyl]-amine | 1 | 1 |
| PR1 | 4-hydroxy-3-[2-oxo-3-(thieno[3,2-b]pyridine-2-Sulfonylamino)-pyrrolidin-1-ylmethyl]-benzamidine | 1 | 1 |
| PR2 | Thieno[3,2-b]pyridine-2-sulfonicAcid[2-oxo-1-(1h-Pyrrolo[2,3-c]pyridin-2-ylmethyl)-pyrrolidin-3-yl]-Amide | 1 | 1 |
| PRB | 13-acetylphorbol | 1 | 1 |
| PRC | N-[4-methyl-3-[[4-(3-pyridinyl)-2-pyrimidinyl]amino]Phenyl]-3-pyridinecarboxamide | 1 | 1 |
| PRD | N6-(2,5-dimethoxy-benzyl)-n6-methyl-pyrido[2,3-d]Pyrimidine-2,4,6-triamine | 1 | 1 |
| PRL | Proflavin | 1 | 1 |
| PRM | 3,8-diamino-5[3-(diethylmethylammonio)propyl]-6-Phenylphenanthridinium | 1 | 1 |
| PRY | 2-propyl-aniline | 1 | 1 |
| PSQ | Ndelta-(n'-sulphodiaminophosphinyl)-l-ornithine | 1 | 1 |
| PTB | (5-tert-butyl-1,3,4-oxadiazol-2-yl)[(2r)-pyrrolidin-2-Yl]methanone | 1 | 1 |
| PTI | 2-phenyl-1-[4-(2-piperidin-1-yl-ethoxy)-phenyl]-1,2,3,4-tetrahydro-isoquinolin-6-ol | 1 | 1 |
| PTO | Pseudotropine | 1 | 1 |
| PTR | O-phosphotyrosine | 1 | 1 |
| PTS | (4s-trans)-4-(amino)-5,6-dihydro-6-methyl-4h-thieno(2,3-b)thiopyran-2-sulfonamide-7,7-dioxide | 1 | 1 |
| PU0 | 9-butyl-8-(2,5-dimethoxy-benzyl)-2-fluoro-9h-purin-6-Ylamine | 1 | 1 |
| PU1 | 8-(2-chloro-3,4,5-trimethoxy-benzyl)-2-fluoro-9-pent-4-ylnyl-9h-purin-6-ylamine | 1 | 1 |
| PU2 | 8-(2,5-dimethoxy-benzyl)-2-fluoro-9h-purin-6-ylamine | 1 | 1 |
| PU4 | 9-butyl-8-(4-methoxybenzyl)-9h-purin-6-amine | 1 | 1 |
| PU5 | 9-butyl-8-(3-methoxybenzyl)-9h-purin-6-amine | 1 | 1 |
| PU6 | 8-benzo[1,3]dioxol-,5-ylmethyl-9-butyl-9h- | 1 | 1 |
| PU7 | 9-butyl-8-(2,5-dimethoxy-benzyl)-9h-purin-6-ylamine | 1 | 1 |
| PU8 | 9-butyl-8-(2-chloro-3,4,5-trimethoxy-benzyl)-9h-purin-6-ylamine | 1 | 1 |
| PU9 | 8-(2-chloro-3,4,5-trimethoxy-benzyl)-9-pent-4-ylnyl-9h-purin-6-ylamine | 1 | 1 |
| PUX | 8-benzo[1,3]dioxol-,5-ylmethyl-9-butyl-2-fluoro-9h-Purin-6-ylamine | 1 | 1 |
| PUZ | 8-(2,5-dimethoxy-benzyl)-2-fluoro-9-pent-9h-purin-6-Ylamine | 1 | 1 |
| PVB | Purvalanol | 1 | 1 |
| PXG | 3-[o-phosphonopyridoxyl]--amino-benzoicAcid | 1 | 1 |
| PXL | 3-hydroxy-5-(hydroxymethyl)-2-methylisonicotinaldehyde | 1 | 1 |
| PXP | Pyridoxine-5'-phosphate | 1 | 1 |
| PY1 | 4-(3-pyridin-2-yl-1h-pyrazol-4-yl)quinoline | 1 | 1 |
| PY3 | 5-[Amino(Imino)Methyl]-2-[({[6-[3-Amino-5-({[(1r)-1 Methylpropyl]Amino}Carbonyl)Phenyl]-3 (Isopropylamino)-2-Oxopyrazin-1(2h)-Yl]Acetyl}Amino) Methyl]-N-Pyridin-4-Ylbenzamide | 1 | 1 |
| PY5 | 2-[o-phosphonopyridoxyl]-amino-pentanoicAcid | 1 | 1 |
| PY6 | 2-[o-phosphonopyridoxyl]-amino-hexanoicAcid | 1 | 1 |
| PYF | 3-pyridinylcarbinol | 1 | 1 |
| PYS | 2-pyridinethiol | 1 | 1 |
| Q82 | {[4-r(-4-alpha,5-alpha,6-beta,7-beta)]-hexahydro-5,6-Bis(hydroxy)-1,3-bis(4-hydroxymethyl)methyl]-4,7-Bis(phenylmethyl)-2h-1,3-diazepin-2-ylidene]cyanamide} | 1 | 1 |
| QSI | 5'-o-[n-(l-glutaminyl)-sulfamoyl]adenosine | 1 | 1 |
| R11 | 4-{[1-methyl-5-(2-methyl-benzoimidazol-1-ylmethyl)-1h-Benzoimidazol-2-ylmethyl]-amino}-benzamidine | 1 | 1 |
| R13 | 3-methyl-7-(5,5,8,8-tetramethyl-5,6,7,8-tetrahydro-Naphthalen-2-yl)-octa-2,4,6-trienoicAcid | 1 | 1 |
| R1P | Ribose-1-phosphate | 1 | 1 |
| R36 | 4-amino-n-{4-[2-(2,6-dimethyl-phenoxy)-acetylamino]-3-Hydroxy-1-isobutyl-5-phenyl-pentyl}-benzamide | 1 | 1 |
| R37 | 3-amino-n-{4-[2-(2,6-dimethyl-phenoxy)-acetylamino]-3-Hydroxy-1-isobutyl-5-phenyl-pentyl}-benzamide | 1 | 1 |
| R56 | 5-oxo-6-phenylmethanesulfonylamino-hexahydro-Thiazolo[3,2-a]pyridine-3-carboxylicAcid(3-guanidino-propyl)-amide | 1 | 1 |
| RAB | 2-(6-amino-purin-9-yl)-5-hydroxymethyl-tetrahydro-Furan-3,4-diol | 1 | 1 |
| RAL | Raloxifene | 1 | 1 |
| RAZ | (1r)-N,4-Dimethyl-N-(4-Methylpent-3-Enyl)Cyclohex-3Enaminium | 1 | 1 |
| RCA | 6-{[4-(hydroxymethyl)-5-methyl-2,6-Dioxohexahydropyrimidin-5-yl]methyl}-5-Methylpyrimidine-2,4(1h,3h)-dione | 1 | 1 |
| RDC | Radicicol | 1 | 1 |
| RDF | N-alpha-l-rhamnopyranosyloxy(hydroxyphosphinyl)-l-Leucyl-l-tryptophan | 1 | 1 |
| RDI | Radicicol | 1 | 1 |
| RDL | 6,7-dioxo-5h-8-ribitylaminolumazine | 1 | 1 |
| RDR | 4-[(6-chloro-1-benzothien-2-yl)sulfonyl]-1-{[1-(2-Hydroxyethyl)-1h-pyrrolo[3,2-c]pyridin-2-yl]methyl}Piperazin-2-one | 1 | 1 |
| RED | DihydrolipoicAcid | 1 | 1 |
| RES | 4-phospho-d-erythronohydroxamicAcid | 1 | 1 |
| REX | Glycyl-l-alpha-amino-epsilon-pimelyl-d-alanyl-d-alanine | 1 | 1 |
| REY | Glycyl-l-alpha-amino-epsilon-pimelyl-d-alanine | 1 | 1 |
| RFL | 8-demethyl-8-dimethylamino-flavin-adenine-dinucleotide | 1 | 1 |
| RH1 | 2,5-diaziridin-1-yl-3-(hydroxymethyl)-6-Methylcyclohexa-2,5-diene-1,4-dione | 1 | 1 |
| RHC | 5-(3-amino-4,4-dihyroxy-butylsulfanylmethyl)-Tetrahydro-furan-2,3,4-triol | 1 | 1 |
| RIS | 1-hydroxy-2-(3-pyridinyl)ethylideneBis-phosphonicAcid | 1 | 1 |
| RLP | 3-(7-hydroxy-8-ribityllumazine-6-yl)PropionicAcid | 1 | 1 |
| RMB | N1-(5'-phospho-alpha-ribosyl)-5-methylbenzimidazole | 1 | 1 |
| RMN | (r)-mandelicAcid | 1 | 1 |
| RNP | (1e,2r)-1-(isopropylimino)-3-(1-naphthyloxy)propan-2-ol | 1 | 1 |
| RNT | L-rhamnitol | 1 | 1 |
| RO4 | [[1-[n-hydroxy-acetamidyl]-3-methyl-butyl]-carbonyl-Leucinyl]-alanineEthylEster | 1 | 1 |
| ROB | 1-deoxy-ribofuranose-5'-phosphate | 1 | 1 |
| ROC | Cis-n-tert-butyl-decahydro-2-[2(r)-hydroxy-4-phenyl-3(s)-[[n-2-quinolylcarbonyl-l-asparaginyl]amino]butyl]-(4as)-isoquinoline-3(s)-carboxamide | 1 | 1 |
| ROI | 7,8-dihydro-7,7-dimethyl-6-hydroxypterin | 1 | 1 |
| RPD | (c8-r)-hydantocidin5'-phosphate | 1 | 1 |
| RPL | (c8-s)-hydantocidin5'-phosphate | 1 | 1 |
| RPN | (r)-1-para-nitro-phenyl-2-azido-ethanol | 1 | 1 |
| RPP | 2-ribofuranosyl-3-iodo-2,3-dihydro-1h-pyrazolo[3,4-d]Pyrimidin-4-ylamine | 1 | 1 |
| RQ3 | 2-{4-[(3,5-dimethylanilino)-carbonyl-methyl]-phenoxy}-2-methylpropionicAcid | 1 | 1 |
| RRC | R-roscovitine | 1 | 1 |
| RRP | 3-({4-[(6-chloro-1-benzothien-2-yl)sulfonyl]-2-Oxopiperazin-1-yl}methyl)benzenecarboximidamide | 1 | 1 |
| RRR | 4-{[(e)-2-(5-chlorothien-2-yl)vinyl]sulfonyl}-1-(1h-Pyrrolo[3,2-c]pyridin-2-ylmethyl)piperazin-2-one | 1 | 1 |
| RRS | N4-hydroxy-2-isobutyl-n1-(9-oxo-1,8-diaza-Tricyclo[10.6.1.013,18]nonadeca-12(19),13,15,17-Tetraen-10-yl)-succinamide | 1 | 1 |
| RS1 | 4-[4-(4-chloro-phenoxy)-benzenesulfonylmethyl]-Tetrahydro-pyran-4-carboxylicAcidHydroxyamide | 1 | 1 |
| RS2 | N-hydroxy-2-[4-(4-phenoxy-benzenesulfonyl)-tetrahydro-Pyran-4-yl]-acetamide | 1 | 1 |
| RS7 | 3-(2-octylphenyl)propanoicAcid | 1 | 1 |
| RSO | R-styreneOxide | 1 | 1 |
| RSS | 5-methyl-3-(9-oxo-1,8-diaza-tricyclo[10.6.1.013,18]Nonadeca-12(19),13,15,17-tetraen-10-ylcarbamoyl)-HexanoicAcid | 1 | 1 |
| RTR | 4-({4-[(6-chloro-1-benzothien-2-yl)sulfonyl]-2-Oxopiperazin-1-yl}methyl)benzenecarboximidamide | 1 | 1 |
| RUP | (r)-2-(3-adamantan-1-yl-ureido)-3-(3-carbamimidoyl-Phenyl)-n-phenethyl-propionamide | 1 | 1 |
| RUT | Rutin | 1 | 1 |
| S11 | 5-(3,5-dichlorophenyl)thio-4-isopropyl-1-(pyridin-4-yl-methyl)-1h-imidazol-2-yl-methylCarbamate | 1 | 1 |
| S1A | SoraphenA | 1 | 1 |
| S27 | N-[[2-methyl-4-hydroxycarbamoyl]but-4-yl-n]-benzyl-p-[phenyl]-p-[methyl]phosphinamid | 1 | 1 |
| S6P | D-Sorbitol-6-Phosphate | 1 | 1 |
| S80 | N1-hydroxy-2-(3-hydroxy-propyl)-3-isobutyl-n4-[1-(2-Methoxy-ethyl)-2-oxo-azepan-3-yl]-succinamide | 1 | 1 |
| SA8 | S-5'-Azamethionine-5'-Deoxyadenosine | 1 | 1 |
| SAB | 4-sulfonamide-[1-(4-aminobutane)]benzamide | 1 | 1 |
| SAF | 3-[(1s)-1-(dimethylamino)ethyl]phenol | 1 | 1 |
| SAS | 2-hydroxy-(5-([4-(2-pyridinylamino)sulfonyl]phenyl)Azo)benzoicAcid | 1 | 1 |
| SB1 | (21s)-1aza-4,4-dimethyl-6,19-dioxa-2,3,7,20-Tetraoxobicyclo[19.4.0]Pentacosane | 1 | 1 |
| SB3 | 1,3-diphenyl-1-propyl-1-(3,3-dimethyl-1,2-dioxypentyl)-2-piperidineCarboxylate | 1 | 1 |
| SB5 | 4-(fluorophenyl)-1-cyclopropylmethyl-5-(2-amino-4-Pyrimidinyl)imidazole | 1 | 1 |
| SB6 | 4-(4-fluorophenyl)-1-cycloropropylmethyl-5-(4-pyridyl)-imidazole | 1 | 1 |
| SBB | N-[2-(1h-indol-5-yl)-butyl]-4-sulfamoyl-benzamide | 1 | 1 |
| SBI | Sorbinil | 1 | 1 |
| SBR | (r)-n-(3-indol-1-yl-2-methyl-propyl)-4-sulfamoyl-Benzamide | 1 | 1 |
| SBS | (s)-n-(3-indol-1-yl-2-methyl-propyl)-4-sulfamoyl-Benzamide | 1 | 1 |
| SBX | 1-cyclohexyl-3-phenyl-1-propyl-1-(3,3-dimethyl-1,2-Dioxypentyl)-2-piperidineCarboxylate | 1 | 1 |
| SCT | (south)-methanocarba-thymidine | 1 | 1 |
| SEH | S-BenzylPhenylacetothiohydroximate-O-Sulfate | 1 | 1 |
| SEP | Phosphoserine | 1 | 1 |
| SFG | Adenosyl-ornithine | 1 | 1 |
| SFM | (13e,15e)-(3s,6s,9r,10r,11s,12s,18s,21s)-10,12-Dihydroxy-3-(3-hydroxyben-zyl)-18-((e)-3-hydroxy-1-Methylpropenyl)-6-isopropyl-11-methyl-9-(3-oxo-butyl)-19-oxa-1,4,7,25-tetraaza-bicyclo[19.3.1]pentacosa-13,15-diene-2,5,8,20-tetraone | 1 | 1 |
| SGL | 1-hydroxy-1-thio-glycerol | 1 | 1 |
| SHH | OctanedioicAcidHydroxyamidePhenylamide | 1 | 1 |
| SHM | Homobiotin | 1 | 1 |
| SHR | N-(5-amino-5-carboxypentyl)glutamicAcid | 1 | 1 |
| SHT | O-phosphono-n-{(2e)-7-[(2-sulfoethyl)dithio]hept-2-Enoyl}-l-threonine | 1 | 1 |
| SIM | Simvastatin | 1 | 1 |
| SKM | (3r,4s,5r)-3,4,5-trihydroxycyclohex-1-ene-1-carboxylicAcid | 1 | 1 |
| SKP | 5-(1-carboxy-1-phosphonooxy-ethoxyl)-4-hydroxy-3-Phosphonooxy-cyclohex-1-enecarboxylicAcid | 1 | 1 |
| SMB | 2-methylbutanoicAcid | 1 | 1 |
| SMD | Methyl-2-s-(alpha-d-mannopyranosyl)-2-thio-alpha-d-Mannopyranoside | 1 | 1 |
| SMG | N-succinylMethionine | 1 | 1 |
| SMS | SulfamicAcid2,3-o-(1-methylethylidene)-4,5-o-Sulfonyl-beta-fructopyranoseEster | 1 | 1 |
| SN2 | 5-[bis-2(chloro-ethyl)-amino]-2,4-dintro-benzamide | 1 | 1 |
| SNP | 1-(isopropylamino)-3-(1-naphthyloxy)-2-propanol | 1 | 1 |
| SO1 | [1r-(1.alpha.,3a.beta.,4.beta.,4a.beta.,7.beta.,7a.alpha.,8a.beta.)]8a-[(6-deoxy-4-o-methyl-beta-d-Altropyranosyloxy)methyl]-4-formyl-4,4a,5,6,7,7a,8,8a-Octahydro-7-methyl-3-(1-methylethyl)-1,4-methano-s-Indacene-3a(1h)-carboxylicAcid | 1 | 1 |
| SON | AdenosinePhosphonoaceticAcid | 1 | 1 |
| SPC | N-hydroxy1n(4-methoxyphenyl)sulfonyl-4-(z,e-n-Methoxyimino)pyrrolidine-2r-carboxamide | 1 | 1 |
| SPI | N-hydroxy-1-(4-methoxyphenyl)sulfonyl-4-Benzyloxycarbonyl-piperazine-2-carboxamide | 1 | 1 |
| SPQ | (3r,4s,5r)-5-{[(1r)-1-carboxy-2-fluoro-1-(phosphonooxy)ethyl]oxy}-4-hydroxy-3-(phosphonooxy)Cyclohex-1-ene-1-carboxylicAcid | 1 | 1 |
| SPV | Sulfopyruvate | 1 | 1 |
| SRL | [2-(3,5-di-tert-butyl-4-hydroxy-phenyl)-1-(diethoxy-Phosphoryl)-vinyl]-phosphonicAcidDiethlylEster | 1 | 1 |
| SRS | 4-methyl-3-(9-oxo-1,8-diaza-tricyclo[10.6.1.0(13,18)]Nonadeca-12(19),13(18),15,17-tetraene-10-carbamoyl)Penta-methylsulfonediimine | 1 | 1 |
| ST2 | 4-(acetylamino)-5-amino-3-hydroxybenzoicAcid | 1 | 1 |
| ST3 | 4-(acetylamino)-3-aminoBenzoicAcid | 1 | 1 |
| ST4 | 4-(acetylamino)-3-guanidinobenzoicAcid | 1 | 1 |
| ST5 | 4-(acetylamino)-3-[(hydroxyacetyl)amino]benzoicAcid | 1 | 1 |
| STC | 3-[(4-chloroanilino)sulfonyl]thiophene-2-carboxylicAcid | 1 | 1 |
| SU1 | 3-[(3-(2-carboxyethyl)-4-methylpyrrol-2-yl)methylene]-2-indolinone | 1 | 1 |
| SU2 | 3-[4-(1-formylpiperazin-4-yl)-benzylidenyl]-2-Indolinone | 1 | 1 |
| SUB | 3-phenyl-2-{[4-(toluene-4-sulfonyl)-thiomorpholine-3-Carbonyl]-amino}-propionicAcidEthylEster | 1 | 1 |
| SUG | N~2~-(3-carboxypropanoyl)-l-arginine | 1 | 1 |
| SUM | 2-[3,4-dihydroxy-2-hydroxymethyl-5-(2-hydroxy-nonyl)-Tetrahydro-furan-2-yloxy]-6-hydroxymethyl-tetraHydro-Pyran-3,4,5-triol | 1 | 1 |
| SUO | N~2~-(3-Carboxypropanoyl)-L-Ornithine | 1 | 1 |
| SWA | 1s-8ab-octahydro-indolizidine-1a,2a,8b-triol | 1 | 1 |
| SXX | Sinapinate | 1 | 1 |
| SYM | 2s,4r-4-methylglutamate | 1 | 1 |
| SYR | Syringate | 1 | 1 |
| T | Thymidine-5'-monophosphate | 1 | 1 |
| T10 | [1-(1-benzyl-3-hydroxy-2-oxo-propylcarbamoyl)-2-phenyl-ethyl]-carbamicAcidBenzylEster | 1 | 1 |
| T1D | 5-(4-Methoxybiphenyl-3-Yl)-1,2,5Thiadiazolidin-3-One1,1-Dioxide | 1 | 1 |
| T1P | 3-{2,6,8-Trioxo-9-[(2r,3s,4r)-2,3,4,5 Tetrahydroxypentyl]-1,2,3,6,8,9-Hexahydro-7h-Purin7-Yl}Propyl DihydrogenPhosphate | 1 | 1 |
| T2D | 5-Phenyl-1,2,5-Thiadiazolidin-3-One1,1Dioxide | 1 | 1 |
| T3 | 3,5,3'triiodothyronine | 1 | 1 |
| T33 | 3,3'-deiodo-thyroxine | 1 | 1 |
| T3P | Thymidine-3'-phosphate | 1 | 1 |
| T4A | 3,3',5,5'-tetraiodothyroaceticAcid | 1 | 1 |
| T6P | Trehalose-6-phosphate | 1 | 1 |
| T76 | (6r,21as)-17-chloro-6-cyclohexyl-2,3,6,7,10,11,19,20-Octahydro-1h,5h-pyrrolo[1,2-k][1,4,8,11,14]Benzoxatetraaza-cycloheptadecine-5,8,12,21(9h,13h,21ah)-tetrone | 1 | 1 |
| T80 | Methylpenta(oxyethyl)Heptadecanoate | 1 | 1 |
| TAB | AceticAcidN-[2-chloro-5-[6-ethyl-2,4-diamino-pyrimid-5-yl]-phenyl]-[benzyl-triazen-3-yl]ethylEster | 1 | 1 |
| TAC | Tetracycline | 1 | 1 |
| TAD | Beta-methylene-thiazole-4-carboxyamide-adenineDinucleotide | 1 | 1 |
| TAL | 9-(6-deoxy-alpha-l-talofuranosyl)-6-methylpurine | 1 | 1 |
| TAM | Tris(hydroxyethyl)aminomethane | 1 | 1 |
| TBH | 5-(2-{2-[(tert-butoxy-hydroxy-methyl)-amino]-1-hydroxy-3-phenyl-propylamino}-3-hydroxy-3-pentylamino-propyl)-2-carboxymethoxy-benzoicAcid | 1 | 1 |
| TBN | '2-(4-amino-pyrrolo[2,3-d]pyrimidin-7-yl)-5-Hydroxymethyl-tetrahydro-furan-3,4-diol | 1 | 1 |
| TCB | Thiocellobiose | 1 | 1 |
| TCC | 2-(2,4-dichloro-phenylamino)-phenol | 1 | 1 |
| TCO | Tert-butyl(1s)-1-cyclohexyl-2-oxoethylcarbamate | 1 | 1 |
| TCT | 6-(4-chloro-2-hydroxy-phenoxy)-naphthalen-2-ol | 1 | 1 |
| TDE | (e)-(2r,3r,4s,5r)-3,4,5-trihydroxy-2-methoxy-8,8-Dimethyl-non-6-enoicAcid((3s,6r)-6-hydroxy-2-oxo-Azepan-3-yl)-amide | 1 | 1 |
| TDG | Thiodigalactoside | 1 | 1 |
| TDI | (3r,4s)-1-[(4-amino-5h-pyrrolo[3,2-d]pyrimidin-7-yl)Methyl]-4-[(methylsulfanyl)methyl]pyrrolidin-3-ol | 1 | 1 |
| TDR | Thymine | 1 | 1 |
| TFM | S-ethyl-n-[4-(trifluoromethyl)phenyl]isothiourea | 1 | 1 |
| TG1 | OctanoicAcid[3s-[3alpha,3abeta,4alpha,6beta,6abeta,7beta,8alpha(z),9balpha]]-6-(acetyloxy)-2,3,-3a,4,5,6,6a,7,8,9b-decahydro-3,3a-dihydroxy-3,6,9-Trimethyl-8-[(2-methyl-1-oxo-2-butenyl)oxy]-2-oxo-4-(1-oxobutoxy)-azuleno[4,5-b]furan-7-ylEster | 1 | 1 |
| TH1 | 2-(Beta-D-Glucopyranosyl)-5-Methyl-Benzothiazole | 1 | 1 |
| THD | 2-[3-[(4-amino-2-methyl-5-pyrimidinyl)methyl]-2-(1,2-Dihydroxyethyl)-4-methyl-1,3-thiazol-3-ium-5-yl]ethylTrihydrogenDiphosphate | 1 | 1 |
| THE | ThreonateIon | 1 | 1 |
| THF | 5-hydroxymethylene-6-hydrofolicAcid | 1 | 1 |
| THG | (6s)-5,6,7,8-tetrahydrofolate | 1 | 1 |
| THK | 4-(4-chloro-phenyl)-1-{3-[2-(4-fluoro-phenyl)-[1,3]Dithiolan-2-yl]-propyl}-piperidin-4-ol | 1 | 1 |
| THL | N-[4-({[(6s)-2-amino-4-oxo-1,4,5,6,7,8-Hexahydropteridin-6-yl]methyl}amino)benzoyl]-l-GlutamicAcid | 1 | 1 |
| THN | 2-[carboxy-(2-thiophen-2-yl-acetylamino)-methyl]-5-Methylene-5,6-dihydro-2h-[1,3]thiazine-4-carboxylicAcid | 1 | 1 |
| THQ | PhosphoricAcidMono-[2-(4-methyl-thiazol-5-yl)-ethyl]Ester | 1 | 1 |
| TI1 | [2(r,s)-2-sulfanylheptanoyl]-phe-ala | 1 | 1 |
| TI2 | (2-sulfanyl-3-phenylpropanoyl)-phe-tyr | 1 | 1 |
| TI3 | [(2s)-2-sulfanyl-3-phenylpropanoyl]-gly-(5-Phenylproline) | 1 | 1 |
| TIO | (2-mercaptomethyl-3-phenyl-propionyl)-glycine | 1 | 1 |
| TLM | Thiolactomycin | 1 | 1 |
| TLP | 3-hydroxy-2-[(3-hydroxy-2-methyl-5-phosphonooxymethyl-Pyridin-4-ylmethyl)-amino]-butyricAcid | 1 | 1 |
| TMF | 5,10-methylene-6-hydrofolicAcid | 1 | 1 |
| TMT | 2,3,4-trimethyl-1,3-thiazole | 1 | 1 |
| TMU | N-(4-methoxybenzyl)-n'-(5-nitro-1,3-thiazol-2-yl)urea | 1 | 1 |
| TMZ | 3,4,5-trimethyl-1,3-thiazole | 1 | 1 |
| TNC | 4-dimethylamino-1,10,11,12-tetrahydroxy-3-oxo-3,4,4a,5-tetrahydro-naphthacene-2-carboxylicAcidAmide | 1 | 1 |
| TNK | 6-benzyl-1-benzyloxymethyl-5-isopropylUracil | 1 | 1 |
| TNL | 2,4,6-trinitrotoluene | 1 | 1 |
| TOL | Tolrestat | 1 | 1 |
| TOY | Tobramycin | 1 | 1 |
| TP3 | 4-[[glutamicAcid]-carbonyl]-benzene-sulfonyl-d-proline | 1 | 1 |
| TP4 | N-[4-[[glutamicAcid]-carbonyl]-benzene-sulfonyl-d-Prolinyl]-3-amino-propanoicAcid | 1 | 1 |
| TPA | Trans-2-phenylcyclopropylamine | 1 | 1 |
| TPF | 2-(2,4-difluorophenyl)-1,3-di(1h-1,2,4-triazol-1-yl)Propan-2-ol | 1 | 1 |
| TPI | 4-carbamoyl-4-{[6-(difluoro-phosphono-methyl)-Naphthalene-2-carbonyl]-amino}-butyricAcid | 1 | 1 |
| TPO | Phosphothreonine | 1 | 1 |
| TPR | Tosyl-d-proline | 1 | 1 |
| TQ3 | 5-phenylsulfanyl-2,4-quinazolinediamine | 1 | 1 |
| TQ4 | 5-[(4-methylphenyl)sulfanyl]-2,4-quinazolinediamine | 1 | 1 |
| TQ5 | 5-[4-tert-butylphenylsulfanyl]-2,4-quinazolinediamine | 1 | 1 |
| TQ6 | 5-(4-morpholin-4-yl-phenylsulfanyl)-2,4-Quinazolinediamine | 1 | 1 |
| TR1 | 2-(2-{2-[(biphenyl-4-ylmethyl)-amino]-3-mercapto-Pentanoylamino}-acetylamino)-3-methyl-butyricAcidMethylEster | 1 | 1 |
| TRT | FragmentOfTritonX-100 | 1 | 1 |
| TSB | 5'-o-(n-(l-threonyl)-sulfamoyl)adenosine | 1 | 1 |
| TST | 4-methyl-2-{[4-(toluene-4-sulfonyl)-thiomorpholine-3-Carbonyl]-amino}-pentanoicAcid | 1 | 1 |
| TSU | Para-tolueneSulfonate | 1 | 1 |
| TSX | N-[4-(1-allyl-3-butyl-2,6-dioxo-2,3,6,7-tetrahydro-1h-Purin-8-ylmethyl)-phenyl]-acetamide | 1 | 1 |
| TTB | 4-[(1e)-2-(5,5,8,8-tetramethyl-5,6,7,8-Tetrahydronaphthalen-2-yl)prop-1-enyl]benzoicAcid | 1 | 1 |
| TTN | Tartronate | 1 | 1 |
| TX5 | (1r,3r)-5-((Z)-2-((1r,7as)-Hexahydro-1-((S)-6-Hydroxy 6-Methylhept-4-Yn-2-Yl)-7a-Methyl-1h-Inden-4(7ah) Ylidene)Ethylidene)Cyclohexane-1,3-Diol | 1 | 1 |
| TYP | Cyclo-(l-tyrosine-l-proline)Inhibitor | 1 | 1 |
| TZ4 | 3,8-diamino-6-phenyl-5-[6-[1-[2-[(1,2,3,4-tetrahydro-9-acridinyl)amino]ethyl]-1h-1,2,3-triazol-4-yl]hexyl]-Phenanthridinium | 1 | 1 |
| TZC | 1,2,4-triazole-carboxamidine | 1 | 1 |
| TZD | 2-{3-[(4-amino-2-methylpyrimidin-5-yl)methyl]-4-methyl-2-oxo-2,3-dihydro-1,3-thiazol-5-yl}ethylTrihydrogenDiphosphate | 1 | 1 |
| U03 | 4-hydroxy-7-methoxy-3-(1-phenyl-propyl)-chromen-2-one | 1 | 1 |
| U04 | ({3-[1-(4-hydroxy-2-oxo-2h-chromen-3-yl)-propyl]-Phenylcarbamoyl}-methyl)-carbamicAcidTert-butylEster | 1 | 1 |
| U05 | 6,11-dihydro-11-ethyl-6-methyl-9-nitro-5h-pyrido[2,3-B][1,5]benzodiazepin-5-one | 1 | 1 |
| U0E | N-[[1-[n-acetamidyl]-[1-cyclohexylmethyl-2-hydroxy-4-Isopropyl]-but-4-yl]-carbonyl]-glutaminyl-arginyl-amide | 1 | 1 |
| U49 | (20s)-19,20,21,22-tetrahydro-19-oxo-5h-18,20-ethano-12,14-etheno-6,10-metheno-18h-benz[d]imidazo[4,3-k][1,6,9,12]oxatriaza-cyclooctadecosine-9-carbonitrile | 1 | 1 |
| U55 | 4-[(6-amino-4-pyrimidinyl)amino]benzenesulfonamide | 1 | 1 |
| U66 | (20s)-19,20,22,23-tetrahydro-19-oxo-5h,21h-18,20-Ethano-12,14-etheno-6,10-methenobenz[d]imidazo[4,3-l][1,6,9,13]oxatriazacyclonoadecosine-9-carbonitrile | 1 | 1 |
| U89 | N-[4-[[3-(2,4-diamino-1,6-dihydro-6-oxo-4-pyrimidinyl)-propyl]-[2-((2-oxo-2-((4-phosphoriboxy)-butyl)-amino)-ethyl)-thio-acetyl]-amino]benzoyl]-1-glutamicAcid | 1 | 1 |
| UA3 | UracilArabinose-3'-phosphate | 1 | 1 |
| UC1 | 2-methyl-furan-3-carbothioicAcid[4-chloro-3-(3-Methyl-but-2-enyloxy)-phenyl]-amide | 1 | 1 |
| UCN | 7-hydroxystaurosporine | 1 | 1 |
| UDX | Uridine-5'-diphosphate-xylopyranose | 1 | 1 |
| UI1 | 6-[(z)-amino(imino)methyl]-n-[4-(aminomethyl)phenyl]-4-(pyrimidin-2-ylamino)-2-naphthamide | 1 | 1 |
| UI2 | 8-(pyrimidin-2-ylamino)naphthalene-2-carboximidamide | 1 | 1 |
| UI3 | 7-methoxy-8-[1-(methylsulfonyl)-1h-pyrazol-4-yl]Naphthalene-2-carboximidamide | 1 | 1 |
| UIN | 5,6,7,8,9,10-hexahydro-4-hydroxy-3-(1-phenylpropyl)Cycloocta[b]pyran-2-one | 1 | 1 |
| UMF | 2'-fluoro-2'-deoxyuridine3'-monophosphate | 1 | 1 |
| UMG | Methyl-umbellifertl-n-acetyl-chitotriose | 1 | 1 |
| UN4 | 5-{[(2-amino-9h-purin-6-yl)oxy]methyl}-2-pyrrolidinone | 1 | 1 |
| UNC | 5-Amino-6-Nitropyrimidine-2,4(1h,3h)-Dione | 1 | 1 |
| UNI | 4-cyano-n-(3-cyclopropyl(5,6,7,8,9,10-hexahydro-4-Hydroxy-2-oxo-cycloocta[b]pyran-3-yl)methyl)phenylBenzensulfonamide | 1 | 1 |
| UNN | (6,7-difluoro-quinazolin-4-yl)-(1-methyl-2,2-diphenyl-Ethyl)-amine | 1 | 1 |
| UPP | Phenyl-uridine-5'-diphosphate | 1 | 1 |
| UR2 | 4-[3-carboxymethyl-3-(4-phosphonooxy-benzyl)-ureido]-4-[(3-cyclohexyl-propyl)-methyl-carbamoyl]butyricAcid | 1 | 1 |
| VIO | N5-(1-imino-3-butenyl)-l-ornithine | 1 | 1 |
| VNL | 4-hydroxy-3-methoxybenzoate | 1 | 1 |
| VPR | 2-propylpentanamide | 1 | 1 |
| VSO | VinylsulphonicAcid | 1 | 1 |
| W11 | 3-{3,5-dimethyl-4-[3-(3-methyl-isoxazol-5-yl)-propoxy]-phenyl}-5-trifluoromethyl-[1,2,4]oxadiazole | 1 | 1 |
| WAC | N-{3-[(7ar,12as,12bs)-7-oxo-1,3,4,6,7,7a,12a,12b-Octahydroindolo[2,3-a]quinolizin-12(2h)-yl]propyl}Propane-2-sulfonamide | 1 | 1 |
| WBU | 5-amino-1h-pyrimidine-2,4-dione | 1 | 1 |
| WRB | 1-[3-(4-bromo-phenoxy)-propoxy]-6,6-dimethyl-1.6-Dihydro-[1,3,5]triazine-2,4-diamine | 1 | 1 |
| X04 | (2-amino-4-formyl-5-hydroxy-6-methylpyridin-3-yl)MethylDihydrogenPhosphate | 1 | 1 |
| XBP | Xylulose-1,5-bisphosphate | 1 | 1 |
| XDP | D-xylulose-2,2-diol-1,5-bisphosphate | 1 | 1 |
| XIL | 3-hydroxy-4-(3,4,5-trihydroxy-tetrahydro-pyran-2-Yloxy)-piperidin-2-one | 1 | 1 |
| XK2 | [4r-(4alpha,5alpha,6beta,7beta)]-hexahydro-5,6-Dihydroxy-1,3-bis[2-naphthyl-methyl]-4,7-Bis(phenylmethyl)-2h-1,3-diazepin-2-one | 1 | 1 |
| XLC | 3-chloro-n-[4-chloro-2-[[(4-chlorophenyl)amino]Carbonyl]phenyl]-4-[(4-methyl-1-piperazinyl)methyl]-2-Thiophenecarboxamide | 1 | 1 |
| XLD | 3-chloro-n-[4-chloro-2-[[(5-chloro-2-pyridinyl)amino]Carbonyl]-6-methoxyphenyl]-4-[[(4,5-dihydro-2-Oxazolyl)methylamino]methyl]-2-thiophenecarboxamide | 1 | 1 |
| XN1 | N-[2-hydroxy-1-indanyl]-5-[(2-Tertiarybutylaminocarbonyl)-4(3-pyridylmethyl)Piperazino]-4-hydroxy-2-(1-phenylethyl)-pentanamide | 1 | 1 |
| XN2 | N-[2-hydroxy-1-indanyl]-5-[(2-Tertiarybutylaminocarbonyl)-4(benzo[1,3]dioxol-5-Ylmethyl)-piperazino]-4-hydroxy-2-(1-phenylethyl)-Pentanamide | 1 | 1 |
| XN3 | N-[2(r)-hydroxy-1(s)-indanyl]-5-[(2(s)-tertiaryButylaminocarbonyl)-4(benzo[1,3]dioxol-5-ylmethyl)-Piperazino]-4(s)-hydroxy-2(r)-phenylmethylpentanamide | 1 | 1 |
| XS2 | Methyl4,4ii,4iii,4iv-Tetrathio-Beta-D-Xylopentoside | 1 | 1 |
| XUL | D-xylulose | 1 | 1 |
| XYA | 2-(6-amino-octahydro-purin-9-yl)-5-hydroxymethyl-Tetrahydro-furan-3,4-diol | 1 | 1 |
| XYD | 2,5-dimethylaniline | 1 | 1 |
| XYH | Xylarohydroxamate | 1 | 1 |
| Y27 | (r)-trans-4-(1-aminoethyl)-n-(4-pyridyl)Cyclohexanecarboxamide | 1 | 1 |
| YPA | (s)-3-(4-(2-carbazol-9-yl-ethoxy)-phenyl)-2-ethoxy-PropionicAcid | 1 | 1 |
| YZ9 | 7-hydroxy-2-oxo-chromene-3-carboxylicAcidEthylEster | 1 | 1 |
| Z34 | N-[2-[5-[amino(imino)methyl]-2-hydroxyphenoxy]-3,5-Difluoro-6-[3-(4,5-dihydro-1-methyl-1h-imidazol-2-yl)Phenoxy]pyridin-4-yl]-n-methylglycine | 1 | 1 |
| ZAF | O-(((1r)-((n-phenylmethoxycarbonyl-l-alanyl)amino)Ethyl)hydroxyphosphono)-l-benzylaceticAcid | 1 | 1 |
| ZAH | N-[(Benzyloxy)Carbonyl]-L-Alanyl-L-Proline | 1 | 1 |
| ZAM | 3-[(acetyl-methyl-amino)-methyl]-4-amino-n-methyl-n-(1-methyl-1h-indol-2-ylmethyl)-benzamide | 1 | 1 |
| ZAR | 6-(4-difluoromethoxy-3-methoxy-phenyl)-2h-pyridazin-3-One | 1 | 1 |
| ZEA | (2z)-2-Methyl-4-(9h-Purin-6-Ylamino)-2-Buten1-Ol | 1 | 1 |
| ZEB | 4-hydroxy-3,4-dihydro-zebularine | 1 | 1 |
| ZES | [3-(4-bromo-2-fluoro-benzyl)-7-chloro-2,4-dioxo-3,4-Dihydro-2h-quinazolin-1-yl]-aceticAcid | 1 | 1 |
| ZIP | N-(3-Methylbut-2-En-1-Yl)-9h-Purin-6-Amine | 1 | 1 |
| ZST | 3,4-dihydro-4-oxo-3-((5-trifluoromethyl-2-Benzothiazolyl)methyl)-1-phthalazineAceticAcid | 1 | 1 |
| ZTW | RaloxifeneCore | 1 | 1 |
| 108 | 2-(5-carbamimidoyl-2-hydroxy-benzylamino)-propionicAcid | 2 | 1 |
| 109 | 2-(4-carbamimidoyl-2-hydroxy-benzylamino)-propionicAcid | 2 | 1 |
| 113 | 7,8-dihydroxy-1-methoxy-3-methyl-10-oxo-4,10-dihydro-1h,3h-pyrano[4,3-b]chromene-9-carboxylicAcid | 2 | 1 |
| 133 | 6-fluoro-2-(2-hydroxy-3-isobutoxy-phenyl)-1h-Benzoimidazole-5-carboxamidine | 2 | 1 |
| 148 | 2-[bis-(2-hydroxy-ethyl)-amino]-2-hydroxymethyl-Propane-1,3-diol | 2 | 1 |
| 334 | 2-{5-[amino(iminio)methyl]-1h-benzimidazol-2-yl}-6-Bromo-4-methylbenzenolate | 2 | 1 |
| 3AT | 3'-deoxyadenosine-5'-triphosphate | 2 | 1 |
| 444 | N-(2,2,2-trifluoroethyl)-n-{4-[2,2,2-trifluoro-1-Hydroxy-1-(trifluoromethyl)ethyl]phenyl}Benzenesulfonamide | 2 | 1 |
| 4AA | 4-Chloro-3-HydroxyanthranilicAcid | 2 | 1 |
| 4AM | 4-amino-2-deoxy-2,3-dehydro-n-neuraminicAcid | 2 | 1 |
| 4BC | 4-(1,3-benzodioxol-5-yl)-5-(5-ethyl-2,4-Dihydroxyphenyl)-2h-pyrazole-3-carboxylicAcid | 2 | 1 |
| 4HY | [4-(4-hydroxy-3-iodo-phenoxy)-3,5-diiodo-phenyl]-AceticAcid | 2 | 1 |
| 4MZ | 4-methylimidazole | 2 | 1 |
| 4PP | (2s)-(3'-amidino-3-biphenyl)-5-(4-pyridylamino)PentanoicAcid | 2 | 1 |
| 5AD | 5'-deoxyadenosine | 2 | 1 |
| 653 | 5-(2-aminoethyl)-3-{5-[amino(iminio)methyl]-1h-Benzimidazol-2-yl}-1,1'-biphenyl-2-olate | 2 | 1 |
| 655 | 2-{5-[amino(iminio)methyl]-1h-benzimidazol-2-yl}-6-(cyclopentyloxy)benzenolate | 2 | 1 |
| 656 | 2-{5-[amino(iminio)methyl]-1h-benzimidazol-2-yl}-6-Isobutoxybenzenolate | 2 | 1 |
| 6FA | 6-hydroxy-flavin-adenineDinucleotide | 2 | 1 |
| 6MP | 6-methylpurine | 2 | 1 |
| 869 | (1-tert-butyl-5-hydroxy-1h-pyrazol-4-yl){6-[dihydroxy(methyl)-lambda~4~-sulfanyl]-4'-methoxy-2-Methyl-1,1'-biphenyl-3-yl}methanone | 2 | 1 |
| 907 | 2-{5-[amino(iminio)methyl]-1h-indol-2-yl}-6-bromo-4-Methylbenzenolate | 2 | 1 |
| 9DA | 9-deazaadenine | 2 | 1 |
| 9PP | 2,6-diamino-(s)-9-[2-(phosphonomethoxy)propyl]purine | 2 | 1 |
| A77 | N-{1-benzyl-(2r,3s)-2,3-dihydroxy-4-[3-methyl-2-(3-Methyl-3-pyridin-2-ylmethyl-ureido)-butyrylamino]-5-Phenyl-pentyl}-3-methyl-2-(3-methyl-3-pyridin-2-Ylmethyl-ureido)-butyramide | 2 | 1 |
| A79 | N-{1-benzyl-(2s,3s)-2,3-dihydroxy-4-[3-methyl-2-(3-Methyl-3-pyridin-2-ylmethyl-ureido)-butyrylamino]-5-Phenyl-pentyl}-3-methyl-2-(3-methyl-3-pyridin-2-Ylmethyl-ureido)-butyramide | 2 | 1 |
| ABY | N-(4-aminobutanoyl)-s-(4-methoxybenzyl)-l-Cysteinylglycine | 2 | 1 |
| ACV | L-d-(a-aminoadipoyl)-l-cysteinyl-d-valine | 2 | 1 |
| ADV | Alpha-betaMethyleneAdp-ribose | 2 | 1 |
| AFB | 1,6,7,8,9,11a,12,13,14,14a-decahydro-1,13-dihydroxy-6-Methyl-4h-cyclopent[f]oxacyclotridecin-4-one | 2 | 1 |
| AG2 | Agmatine | 2 | 1 |
| AIH | (2s,3r)-2-(4-{2-[(3r,4r)-3,4-dimethylpyrrolidin-1-yl]Ethoxy}phenyl)-3-(4-hydroxyphenyl)-2,3-dihydro-1,4-Benzoxathiin-6-ol | 2 | 1 |
| AIQ | 2,6-diamino-8-(1h-imidazol-2-ylsulfanylmethyl)-3h-Quinazoline-4-one | 2 | 1 |
| AIT | (2s,3r)-3-(4-hydroxyphenyl)-2-(4-{[(2r)-2-pyrrolidin-1-ylpropyl]oxy}phenyl)-2,3-dihydro-1,4-benzoxathiin-6-Ol | 2 | 1 |
| AM1 | (s)-2-amino-3-(3-carboxy-5-methylisoxazol-4-yl)PropionicAcid | 2 | 1 |
| AMO | Aspartyl-adenosine-5'-monophosphate | 2 | 1 |
| ANG | 8-aminoguanine | 2 | 1 |
| ANH | MethylN-[(4-methylphenyl)sulfonyl]glycyl-3-[amino(imino)methyl]-d-phenylalaninate | 2 | 1 |
| ARE | AcarboseDerivedPentasaccharide | 2 | 1 |
| AZA | 8-azaxanthine | 2 | 1 |
| BCA | 4-hydroxybenzoylCoenzymeA | 2 | 1 |
| BDD | Bromo-dodecanol | 2 | 1 |
| BDP | Beta-d-galactopyranuronicAcid | 2 | 1 |
| BEB | N,n-[2,5-o-dibenzyl-glucaryl]-di-[1-amino-indan-2-ol] | 2 | 1 |
| BI2 | 3-(1h-indol-3-yl)-4-(1-{2-[(2s)-1-methylpyrrolidinyl]Ethyl}-1h-indol-3-yl)-1h-pyrrole-2,5-dione | 2 | 1 |
| BM6 | 4-[2-(5,5,8,8-tetramethyl-5,6,7,8-tetrahydro-Naphthalen-2-yl)-[1,3]dioxolan-2-yl]-benzoicAcid | 2 | 1 |
| BMP | 6-hydroxyuridine-5'-phosphate | 2 | 1 |
| BMZ | 2-(2-hydroxy-phenyl)-1h-benzoimidazole-5-carboxamidine | 2 | 1 |
| BRH | (s)-2-amino-3-(4-bromo-3-hydroxy-isoxazol-5-yl)PropionicAcid | 2 | 1 |
| BSI | 2-(biphenyl-4-sulfonyl)-1,2,3,4-tetrahydro-Isoquinoline-3-carboxylicAcid | 2 | 1 |
| BZD | N-benzoyl-n'-beta-d-glucopyranosylUrea | 2 | 1 |
| BZI | Benzimidazole | 2 | 1 |
| BZQ | Diphenylmethanone | 2 | 1 |
| C20 | Acetyl-nh-val-cyclohexyl-ch2[nch2choh]ch2-benzyl-val-Nh-acetyl | 2 | 1 |
| C2P | Cytidine-2'-monophosphate | 2 | 1 |
| CAO | OxidizedCoenzymeA | 2 | 1 |
| CBP | 2-{4-[4-(4-chloro-phenoxy)-benzenesulfonyl]-tetrahydro-pyran-4-yl}-n-hydroxy-acetamide | 2 | 1 |
| CE2 | 3-(5-tert-butyl-3-oxidoisoxazol-4-yl)-l-alaninate | 2 | 1 |
| CNH | 2-hydroxy-2-methylpropanenitrile | 2 | 1 |
| CO4 | 2,4-diamino-5-methyl-6-[(3,4,5-trimethoxy-n-Methylanilino)methyl]pyrido[2,3-d]pyrimidine | 2 | 1 |
| COP | N-(4-carboxy-4-{4-[(2,4-diamino-pteridin-6-ylmethyl)-Amino]-benzoylamino}-butyl)-phthalamicAcid | 2 | 1 |
| CPW | (S)-2-Amino-3-(1,3,5,7-Pentahydro-2,4-Dioxo Cyclopenta[E]Pyrimidin-1-Yl)ProionicAcid | 2 | 1 |
| CR6 | 1-deoxy-1-acetylamino-beta-d-gluco-2-Heptulopyranosonamide | 2 | 1 |
| CRP | ((1rs,3sr)-2,2-dichloro-n-[(r)-1-(4-chlorophenyl)Ethyl]-1-ethyl-3-methylcyclopropanecarboxamide | 2 | 1 |
| CTC | 7-chlorotetracycline | 2 | 1 |
| CXF | Cyclohexylformamide | 2 | 1 |
| CYS | Cysteine | 2 | 1 |
| DAD | 2',3'-dideoxyadenosine-5'-triphosphate | 2 | 1 |
| DAR | D-arginine | 2 | 1 |
| DBQ | Debromohymenialdisine | 2 | 1 |
| DCB | 2,4-dinitrophenyl-2-deoxy-2-fluoro-beta-d-cellobioside | 2 | 1 |
| DDU | 2'-5'dideoxyuridine | 2 | 1 |
| DFB | 2,3-difluorobenzylAlcohol | 2 | 1 |
| DFV | 7-hydroxy-2-(4-hydroxy-phenyl)-chroman-4-one | 2 | 1 |
| DII | MethylphosphonicAcidDiisopropylEster | 2 | 1 |
| DMI | 2,3-dimethylimidazoliumIon | 2 | 1 |
| DMQ | [4-r-(-4-alpha,5-alpha,6-beta,7-beta)]-hexahydro-5,6-Bis(hydroxy)-1,3-bis([(3-amino)phenyl]methyl)-4,7-Bis(phenylmethyl)-2h-1,3-diazepinone | 2 | 1 |
| DNN | 7,8-diamino-nonanoicAcid | 2 | 1 |
| DNQ | 6,7-dinitroquinoxaline-2,3-dione | 2 | 1 |
| DOM | 2'-deoxymaltose | 2 | 1 |
| DP3 | N-{(4s)-4-amino-5-[(2-aminoethyl)amino]pentyl}-n'-Nitroguanidine | 2 | 1 |
| DP9 | L-n(omega)-nitroarginine-(4r)-amino-l-prolineAmide | 2 | 1 |
| DPC | 5-acetylamino-4-amino-6-(phenethyl-propyl-carbamoyl)-5,6-dihydro-4h-pyran-2-carboxylicAcid | 2 | 1 |
| DPS | 3-(1h-indol-3-yl)-2-[4-(4-phenyl-piperidin-1-yl)-Benzenesulfonylamino]-propionicAcid | 2 | 1 |
| DQN | Duroquinone | 2 | 1 |
| DQU | 2,6-diamino-3h-quinazolin-4-one | 2 | 1 |
| DTM | 2,4-diamino-6-[n-(3',4',5'-trimethoxybenzyl)-n-Methylamino]pyrido[2,3-d]pyrimidine | 2 | 1 |
| DUR | 2'-deoxyuridine | 2 | 1 |
| EBS | 3-ethyl-2-[(2z)-2-(3-ethyl-6-sulfo-1,3-benzothiazol-2(3h)-ylidene)hydrazino]-6-sulfo-3h-1,3-benzothiazol-1-ium | 2 | 1 |
| EFZ | (-)-6-chloro-4-cyclopropylethynyl-4-trifluoromethyl-1,4-dihydro-2h-3,1-benzoxazin-2-one | 2 | 1 |
| EP1 | 3-[4-(2-hydroxyethyl)piperazin-1-yl]propane-1-sulfonicAcid | 2 | 1 |
| F2B | N-(2,3-difluoro-benzyl)-4-sulfamoyl-benzamide | 2 | 1 |
| F6B | N-(2,6-diflouro-benzyl)-4-sulfamoyl-benzamide | 2 | 1 |
| FA1 | 2,3-anhydro-quinicAcid | 2 | 1 |
| FDM | 3'-fluoro-3'-deoxythymidineMonophosphate | 2 | 1 |
| FFB | N-(2,3,4,5,6-pentaflouro-benzyl)-4-sulfamoyl-benzamide | 2 | 1 |
| FHC | 2-fluoro-3-(4-hydroxyphenyl)-2e-propeneoate | 2 | 1 |
| FIP | 5-fluoroindolePropanolPhosphate | 2 | 1 |
| FMP | Formycin-5'-monophosphate | 2 | 1 |
| FOM | 3-[formyl(hydroxy)amino]propylphosphonicAcid | 2 | 1 |
| FSB | N-(2-flouro-benzyl)-4-sulfamoyl-benzamide | 2 | 1 |
| G16 | Alpha-d-glucose1,6-bisphosphate | 2 | 1 |
| G20 | 4-acetyl-4-guanidino-6-methyl(propyl)carboxamide-4,5-Dihydro-2h-pyran-2-carboxylicAcid | 2 | 1 |
| G28 | 5-n-acetyl-4-amino-6-diethylcarboxamide-4,5-dihydro-2h-pyran-2-carboxylicAcid | 2 | 1 |
| G39 | 5-n-acetyl-3-(1-ethylpropyl)-1-cyclohexene-1-CarboxylicAcid | 2 | 1 |
| G3D | Guanosine-3'-monophosphate-5'-diphosphate | 2 | 1 |
| GCO | GluconicAcid | 2 | 1 |
| GCV | 4-o-methyl-alpha-d-glucuronicAcid | 2 | 1 |
| GDX | Guanosine5'-(trihydrogenDiphosphate),P'-d-MannopyranosylEster | 2 | 1 |
| GLO | D-glucoseInLinearForm | 2 | 1 |
| GLR | 2,3-dihydroxy-5-oxo-hexanedioate | 2 | 1 |
| GLS | Beta-d-glucopyranoseSpirohydantoin | 2 | 1 |
| GLV | GlyoxylicAcid | 2 | 1 |
| GNA | 2,4-deoxy-4-guanidino-5-n-acetyl-neuraminicAcid | 2 | 1 |
| GNH | AminophosphonicAcid-guanylateEster | 2 | 1 |
| GTR | GalacturonicAcid | 2 | 1 |
| HAP | (n-(2-hydroxamatemethylene-4-methyl-pentoyl)Phenylalanyl)methylAmine | 2 | 1 |
| HCG | Delta-(L-Alpha-Aminoadipoyl)-L-CysteinylGlycine | 2 | 1 |
| HE1 | 4-(2-hydroxyphenylthio)-1-butenylphosphonicAcid | 2 | 1 |
| HE3 | 2-hydroxyethylDihydrothiachromeDiphosphate | 2 | 1 |
| HPR | 6-hydroxy-7,8-dihydroPurineNucleoside | 2 | 1 |
| I84 | [2,6-dimethyl-4-(2-o-tolyl-acetylamino)-Benzenesulfonyl]-glycine | 2 | 1 |
| IAG | N-[1h-indol-3-yl-acetyl]glycineAcid | 2 | 1 |
| ICP | 2-methyl-5-methylene-5h-pyrimidin-4-ylideneamine | 2 | 1 |
| ID5 | [5-fluoro-2-({[(4,5,7-trifluoro-1,3-benzothiazol-2-yl)Methyl]amino}carbonyl)phenoxy]aceticAcid | 2 | 1 |
| IFG | (2r,3r,4s,5r)-2-acetamido-3,4-dihydroxy-5-Hydroxymethyl-piperidinium | 2 | 1 |
| IFL | (3s,4r,5r)-3,4-dihydroxy-5-(hydroxymethyl)piperidin-2-One | 2 | 1 |
| IGN | {[(1r)-2-((2s)-2-{[(3-{[amino(imino)methyl]amino}Propyl)amino]carbonyl}piperidinyl)-1-(cyclohexylmethyl)-2-oxoethyl]amino}aceticAcid | 2 | 1 |
| IHN | 3-hydroxy-4-isobutyl-4-[aminocarbonylethyl(aminoCarbonyl-2-hydroxy-5-methylhexyl)tri(aminocarbonylIsobutyl)]butanoicAcid | 2 | 1 |
| IHP | InositolHexakisphosphate | 2 | 1 |
| IMA | [4-({[5-benzyloxy-1-(3-carbamimidoyl-benzyl)-1h-indole-2-carbonyl]-amino}-methyl)-phenyl]-trimethyl-ammonium | 2 | 1 |
| IMU | PhosphoricAcidMono-[5-(2-amino-4-oxo-4,5-dihydro-3h-Pyrrolo[3,2-d]pyrimidin-7-yl)-3,4-dihydroxy-pyrrolidin-2-ylmethyl]Ester | 2 | 1 |
| IN5 | {1-[(3-hydroxy-methyl-5-phosphonooxy-methyl-pyridin-4-Ylmethyl)-amino]-ethyl}-phosphonicAcid | 2 | 1 |
| INI | 5-nitro-6-ribityl-amino-2,4(1h,3h)-pyrimidinedione | 2 | 1 |
| INO | 2-hydroxyisonicotinicAcidN-oxide | 2 | 1 |
| IOP | IndolylpropionicAcid | 2 | 1 |
| IPM | 3-isopropylmalicAcid | 2 | 1 |
| IPT | Isopropyl-1-beta-d-thiogalactoside | 2 | 1 |
| ISA | 3-(4-iodo-phenyl)-2-mercapto-propionicAcid | 2 | 1 |
| ITU | Ethylisothiourea | 2 | 1 |
| IUR | 5-iodouracil | 2 | 1 |
| IWD | 2-amino-3-(5-iodo-2,4-dioxo-3,4-dihydro-2h-pyrimidin-1-yl)-propionicAcid | 2 | 1 |
| K57 | 3-[2-hydroxy-3-(3-hydroxy-2-methyl-benzoylamino)-4-Phenyl-butyryl]-5,5-dimethyl-thiazolidine-4-carboxylicAcidTert-butylamide | 2 | 1 |
| K64 | 3-[2-hydroxy-3-(3-hydroxy-2-methyl-benzoylamino)-4-Phenyl-butyryl]-5,5-Dimethyl-thiazolidine-4-CarboxylicAcid2-methyl-benzylamide | 2 | 1 |
| KTN | Cis-1-acetyl-4-(4-((2-(2,4-dichlorophenyl)-2-(1h-Imidazol-1-ylmethyl)-1,3-dioxolan-4-yl)methoxy)phenyl)Piperazine | 2 | 1 |
| LG6 | L-guluronicAcid6-phosphate | 2 | 1 |
| LIO | [1-pentadecanoyl-2-decanoyl-glycerol-3-yl]phosphonylCholine | 2 | 1 |
| LP1 | 4-[2-(2-acetylamino-3-naphtalen-1-yl-propionylamino)-4-methyl-pentanoylamino]-3-hydroxy-6-methyl-heptanoicAcid[1-(1-carbamoyl-2-naphthalen-1-yl-ethylcarbamoyl)-propyl]-amide | 2 | 1 |
| LPR | [n2-[(s)-1-carboxy-3-phenylpropyl]-l-lysyl-l-proline | 2 | 1 |
| MAH | 3-hydroxy-3-methyl-glutaricAcid | 2 | 1 |
| MDR | 9-(2-deoxy-beta-d-ribofuranosyl)-6-methylpurine | 2 | 1 |
| MEL | [((1r)-2-{(2s)-2-[({4-[amino(imino)methyl]benzyl}Amino)carbonyl]azetidinyl}-1-cyclohexyl-2-oxoethyl)Amino]aceticAcid | 2 | 1 |
| MGP | 7-methyl-guanosine-5'-triphosphate | 2 | 1 |
| MTH | 2-(4-amino-pyrrolo[2,3-d]pyrimidin-7-yl)-5-Methylsulfanylmethyl-tetrahydro-furan-3,4-diol | 2 | 1 |
| NEC | N-ethyl-5'-carboxamidoAdenosine | 2 | 1 |
| NGT | 3ar,5r,6s,7r,7ar-5-hydroxymethyl-2-methyl-5,6,7,7a-Tetrahydro-3ah-pyrano[3,2-d]thiazole-6,7-diol | 2 | 1 |
| NNO | 6-hydroxyisonicotinicAcidN-oxide | 2 | 1 |
| NPI | 2-aminopimelicAcid | 2 | 1 |
| NPL | N-methyl-4-deoxy-4-amino-pyridoxal-5-phosphate | 2 | 1 |
| NTA | NitrilotriaceticAcid | 2 | 1 |
| OCA | OctanoicAcid(caprylicAcid) | 2 | 1 |
| OGA | N-oxalyolglycine | 2 | 1 |
| OHH | 2-[3-({methyl[1-(2-naphthoyl)piperidin-4-yl]amino}Carbonyl)-2-naphthyl]-1-(1-naphthyl)-2-OxoethylphosphonicAcid | 2 | 1 |
| OKA | OkadaicAcid | 2 | 1 |
| OPA | 2-(oxalyl-amino)-4,7-dihydro-5h-thieno[2,3-c]pyran-3-CarboxylicAcid | 2 | 1 |
| OSB | 2-succinylbenzoate | 2 | 1 |
| OTE | 2-{2-[2-(2-octyloxy-ethoxy)-ethoxyl]-ethoxy}ethanol | 2 | 1 |
| OTS | 4-(2s-amino-1-hydroxyethyl)phenol | 2 | 1 |
| PAC | 2-phenylaceticAcid | 2 | 1 |
| PAI | {[(2,2-dihydroxy-ethyl)-(2,3,4,5-tetrahydroxy-6-Phosphonooxy-hexyl)-amino]-methyl}-phosphonicAcid | 2 | 1 |
| PAJ | PantoylAdenylate | 2 | 1 |
| PBN | 4-phenylbutylamine | 2 | 1 |
| PDC | Pyridine-2,6-dicarboxylicAcid | 2 | 1 |
| PLA | 2-[(3-hydroxy-2-methyl-5-phosphonooxymethyl-pyridin-4-Ylmethyl)-amino]-2-methyl-succinicAcid | 2 | 1 |
| PLG | N-glycine-[3-hydroxy-2-methyl-5-phosphonooxymethyl-Pyridin-4-yl-methane] | 2 | 1 |
| PLU | LeucinePhosphonicAcid | 2 | 1 |
| PP6 | Methyl[cyclo-7[(2r)-((n-valyl)amino)-2-(hydroxyl-(1s)-1-methyloxycarbonyl-2-phenylethoxy)phosphinyloxy-Ethyl]-1-naphthaleneacetamide] | 2 | 1 |
| PPE | 4-[(1,3-dicarboxy-propylamino)-methyl]-3-hydroxy-2-Methyl-5-phosphonooxymethyl-pyridinium | 2 | 1 |
| PRA | 3-phenylpropylamine | 2 | 1 |
| PRX | Adenosine-5'-monophosphate-propylEster | 2 | 1 |
| PSI | 2-(2-{5-[2-(2-amino-propionylamino)-propionylamino]-4-Hydroxy-6-phenyl-hexanoylamino}-3-methyl-butyrylamino)-3-methyl-butyricAcidMethylEster | 2 | 1 |
| PTU | 2-ethyl-1-phenyl-isothiourea | 2 | 1 |
| PU3 | 9-butyl-8-(3,4,5-trimethoxybenzyl)-9h-purin-6-amine | 2 | 1 |
| PUR | PurineRiboside | 2 | 1 |
| PYC | Pyrrole-2-carboxylate | 2 | 1 |
| PYQ | Pyroquilon | 2 | 1 |
| Q50 | {(1s)-1-benzyl-4-[3-carbamoyl-1-(1-carbamoyl-2-phenyl-Ethylcarbamoyl)-(s)-propylcarbamoyl]-2-oxo-5-phenyl-Pentyl}-carbamicAcidTert-butylEster | 2 | 1 |
| RCO | Resorcinol | 2 | 1 |
| RIC | PhosphoricAcidMono-[5-(5,6-dimethyl-benzoimidazol-1-Yl)-3,4-dihydroxy-tetrahydro-furan-2ylmethyl]Ester | 2 | 1 |
| RIO | Ribostamycin | 2 | 1 |
| RP1 | 6-(6-amino-purin-9-yl)-2-thioxo-tetrahydro-2-furo[3,2-D][1,3,2]dioxaphosphinine-2,7-diol | 2 | 1 |
| RPR | 3-[(3'-aminomethyl-biphenyl-4-carbonyl)-amino]-2-(3-Carbamimidoyl-benzyl)-butyricAcidMethylEster | 2 | 1 |
| RUB | Ribulose-1,5-diphosphate | 2 | 1 |
| RVP | RibavirinMonophosphate | 2 | 1 |
| SB2 | 4-[5-(4-fluoro-phenyl)-2-(4-methanesulfinyl-phenyl)-3h-imidazol-4-yl]-pyridine | 2 | 1 |
| SB4 | 4-(4-fluorophenyl)-1-(4-piperidinyl)-5-(2-amino-4-Pyrimidinyl)-imidazole | 2 | 1 |
| SHA | SalicylhydroxamicAcid | 2 | 1 |
| SHI | (s)-2-amino-3-(3-hydroxy-isoxazol-4-yl)propionicAcid | 2 | 1 |
| SKD | 2-acetylamino-7-(1,2-dihydroxy-ethyl)-3-hydroxy-6,8-Dioxa-bicyclo[3.2.1]octane-5-carboxylicAcid | 2 | 1 |
| SNR | Norbiotin | 2 | 1 |
| SOX | N-[(2s,4s,6r)-2-(dihydroxymethyl)-4-hydroxy-3,3-Dimethyl-7-oxo-4lambda~4~-thia-1-azabicyclo[3.2.0]hept-6-yl]-2-phenylacetamide | 2 | 1 |
| SSB | 3-butylthiolane1-oxide | 2 | 1 |
| SSD | 1,4-dideoxy-1,4-[[2r,3r)-2,4-dihydroxy-3-(sulfoxy)Butyl]episulfoniumylidene]-d-arabinitolInnerSalt | 2 | 1 |
| ST1 | 4-(acetylamino)-3-hydroxy-5-nitrobenzoicAcid | 2 | 1 |
| STB | 4-(acetylamino)-3-[(aminoacetyl)amino]benzoicAcid | 2 | 1 |
| T44 | 3,5,3',5'-tetraiodo-l-thyronine | 2 | 1 |
| TIH | Beta(2-thienyl)alanine | 2 | 1 |
| TMC | 1-[4-hydroxy-5-(hydroxymethyl)bicyclo[3.1.0]hex-2-yl]-5-methylpyrimidine-2,4(1h,3h)-dione | 2 | 1 |
| TNE | 8-methyl-8-azabicyclo[3,2,1]octan-3-one | 2 | 1 |
| TOP | Trimethoprim | 2 | 1 |
| TP2 | N-[tosyl-d-prolinyl]amino-ethanethiol | 2 | 1 |
| TPV | N-(3-{(1r)-1-[(6r)-4-hydroxy-2-oxo-6-phenethyl-6-Propyl-5,6-dihydro-2h-pyran-3-yl]propyl}phenyl)-5-(trifluoromethyl)-2-pyridinesulfonamide | 2 | 1 |
| TQD | 6-{[methyl(3,4,5-trimethoxycyclohexyl)amino]methyl}Decahydroquinazoline-2,4-diamine | 2 | 1 |
| TQT | 6-(octahydro-1h-indol-1-ylmethyl)decahydroquinazoline-2,4-diamine | 2 | 1 |
| TRC | TricarballylicAcid | 2 | 1 |
| TSN | TrichostatinA | 2 | 1 |
| TYL | N-(4-hydroxyphenyl)acetamide(tylenol) | 2 | 1 |
| TZE | 2-(4-methyl-thiazol-5-yl)-ethanol | 2 | 1 |
| UFG | Uridine-5'-diphosphate-4-deoxy-4-fluoro-alpha-d-Galactose | 2 | 1 |
| UNA | Undecanal | 2 | 1 |
| URI | Uridine | 2 | 1 |
| URO | (2e)-3-(1h-imidazol-4-yl)acrylicAcid | 2 | 1 |
| W05 | Delta-(L-Alpha-Aminoadipoyl)-L-Cysteinyl-DAlanine | 2 | 1 |
| WRA | 6,6-dimethyl-1-[3-(2,4,5-trichlorophenoxy)propoxy]-1,6-dihydro-1,3,5-triazine-2,4-diamine | 2 | 1 |
| PQ0 | 2-amino-4-oxo-4,7-dihydro-3h-pyrrolo[2,3-d]pyrimidine-5-carbonitrile | 2 | 1 |
| QUE | 3,5,7,3',4'-pentahydroxyflavone | 2 | 1 |
| 137 | 1-(o-carboxy-phenylamino)-1-deoxy-d-ribulose-5-Phosphate | 3 | 1 |
| 13P | 1,3-dihydroxyacetonephosphate | 3 | 1 |
| 3IN | N-[2(s)-cyclopentyl-1(r)-hydroxy-3(r)methyl]-5-[(2(s)-Tertiary-butylamino-carbonyl)-4-(n1-(2)-(n-Methylpiperazinyl)-3-chloro-pyrazinyl-5-carbonyl)-Piperazino]-4(s)-hydroxy-2(r)-phenylmethyl-pentanamide | 3 | 1 |
| 762 | 3-{5-[amino(iminio)methyl]-6-chloro-1h-benzimidazol-2-Yl}-1,1'-biphenyl-2-olate | 3 | 1 |
| 785 | 2-{5-[amino(iminio)methyl]-1h-benzimidazol-2-yl}-4-(trifluoromethoxy)benzenolate | 3 | 1 |
| ACA | 6-aminohexanoicAcid | 3 | 1 |
| AD3 | 3-deaza-adenosine | 3 | 1 |
| ASV | Delta-(l-alpha-aminoadipoyl)-l-cysteinyl-d-vinylglycine | 3 | 1 |
| BAH | Bis(5-amidino-2-benzimidazolyl)methaneKetoneHydrate | 3 | 1 |
| BAI | (5-amidino-2-benzimidazolyl)(2-benzimidazolyl)methane | 3 | 1 |
| BCZ | 3-(1-acetylamino-2-ethyl-butyl)-4-guanidino-2-hydroxy-CyclopentanecarboxylicAcid | 3 | 1 |
| BWD | 2-amino-3-(5-bromo-2,4-dioxo-3,4-dihydro-2h-pyrimidin-1-yl)-propionicAcid | 3 | 1 |
| BX3 | (+)-2-[4-[(-1-acetimidoyl-4-piperidinyl)oxy]-3-(7-Amidino-2-naphthyl)propionicAcid | 3 | 1 |
| CLM | Chloramphenicol | 3 | 1 |
| CMC | CarboxymethylCoenzyme*a | 3 | 1 |
| DOB | 2,4-dihydroxybenzoicAcid | 3 | 1 |
| DP1 | L-n(omega)-nitroarginine-2,4-l-diaminobutyricAmide | 3 | 1 |
| DSD | 7-(carboxyamino)-8-amino-nonanoicAcid | 3 | 1 |
| DUD | Deoxyuridine-5'-diphosphate | 3 | 1 |
| ESP | Thieno[2,3-b]pyridine-2-carboxamidine | 3 | 1 |
| FMB | FormycinB | 3 | 1 |
| FPY | (4s)-5-fluoro-4-hydroxy-3,4-dihydropyrimidin-2(1h)-one | 3 | 1 |
| GRO | R-2-phenyl-proprionicAcid | 3 | 1 |
| HUB | HuperzineB | 3 | 1 |
| HY1 | Phenylacetaldehyde | 3 | 1 |
| IK2 | 4'-deoxy-4'-acetylyamino-pyridoxal-5'-phosphate | 3 | 1 |
| INE | 3-bromo-7-nitroindazole | 3 | 1 |
| LAR | LatrunculinA | 3 | 1 |
| M7G | 7n-methyl-8-hydroguanosine-5'-diphosphate | 3 | 1 |
| MJI | 1-hexadecyl-3-trifluoroethyl-sn-glycero-2-phosphateMethane | 3 | 1 |
| MRC | Mupirocin | 3 | 1 |
| NRG | N-omega-nitro-l-arginine | 3 | 1 |
| PAH | PhosphonoacetohydroxamicAcid | 3 | 1 |
| PEA | 2-phenylethylamine | 3 | 1 |
| PFZ | 2-(phosphonooxy)butanoicAcid | 3 | 1 |
| PHY | 1(s)-aminoethyl-(2-carboxypropyl)phosphoryl-phosphinicAcid | 3 | 1 |
| PLS | [3-hydroxy-2-methyl-5-phosphonooxymethyl-pyridin-4-Ylmethyl]-serine | 3 | 1 |
| PPD | 2-[(3-hydroxy-2-methyl-5-phosphonooxymethyl-pyridin-4-Ylmethylene)-amino]-succinicAcid | 3 | 1 |
| PPG | 4-(2-amino-ethoxy)-2-[(3-hydroxy-2-methyl-5-Phosphonooxymethyl-pyridin-4-ylmethyl)-amino]-but-3-EnoicAcid | 3 | 1 |
| PRH | 6-hydroxy-1,6-dihydroPurineNucleoside | 3 | 1 |
| PRZ | 2-isobutyl-3-methoxypyrazine | 3 | 1 |
| SAA | (s)-atrolacticAcid | 3 | 1 |
| SCV | N6-[(1s)-2-{[(1r)-1-carboxy-2-methylpropyl]oxy}-1-(mercaptocarbonyl)-2-oxoethyl]-6-oxo-l-lysine | 3 | 1 |
| SGP | Guanosine-2',3'-cyclophosphorothioate | 3 | 1 |
| T87 | [(1-{2[(4-carbamimidoyl-phenylamino)-methyl]-1-methyl-1h-benzoimidazol-5-yl}-cyclopropyl)-pyridin-2-yl-Methyleneaminooxy]-aceticAcidEthylEster | 3 | 1 |
| TAU | 2-aminoethanesulfonicAcid | 3 | 1 |
| TEO | MalateLikeIntermediate | 3 | 1 |
| TNF | PicricAcid | 3 | 1 |
| TYB | Tyrosinal | 3 | 1 |
| TZL | 2-(sec-butyl)thiazole | 3 | 1 |
| Y3 | 4-acetylamino-5-hydroxynaphthalene-2,7-disulfonicAcid | 3 | 1 |
| 121 | 2-(3-hydroxy-pyridin-2-yl)-1h-benzoimidazole-5-Carboxamidine | 4 | 1 |
| 132 | 6-chloro-2-(2-hydroxy-biphenyl-3-yl)-1h-indole-5-Carboxamidine | 4 | 1 |
| 152 | Carnitine | 4 | 1 |
| 2DT | 3'-deoxythymidine-5'-monophosphate | 4 | 1 |
| 700 | [5-chloro-1h-indol-2-carbonyl-phenylalaninyl]-Azetidine-3-carboxylicAcid | 4 | 1 |
| BAB | Bis(5-amidino-benzimidazolyl)methane | 4 | 1 |
| BB2 | Actinonin | 4 | 1 |
| BLG | 4-o-(4-o-sulfonyl-n-acetylglucosamininyl)-5-Methylhydroxy-l-proline-taurine | 4 | 1 |
| BNI | 5-(2-oxo-hexahydro-thieno[3,4-d]imidazol-6-yl)-PentanoicAcid(4-nitro-phenyl)-amide | 4 | 1 |
| DMP | [4-r-(-4-alpha,5-alpha,6-beta,7-beta)]-hexahydro-5,6-Bis(hydroxy)-[1,3-bis([4-hydroxymethyl-phenyl]methyl)-4,7-bis(phenylmethyl)]-2h-1,3-diazepinone | 4 | 1 |
| EQP | (4-acetamido-2,4-dideoxy-d-glycero-alpha-d-galacto-1-Octopyranosyl)phosphonicAcid | 4 | 1 |
| F89 | S)-2-(5(((1,2-dihydro-3-methyl-1-oxobenzo(f)quinazolin-9-yl)methyl)amino)1-oxo-2-isoindolinyl)glutaricAcid | 4 | 1 |
| GDB | 1-(s-glutathionyl)-2,4-dinitrobenzene | 4 | 1 |
| GEL | 1-o-octyl-2-heptylphosphonyl-sn-glycero-3-Phosphoethanolamine | 4 | 1 |
| HAR | N-omega-hydroxy-l-arginine | 4 | 1 |
| HDA | Hadacidin | 4 | 1 |
| IGP | Indole-3-glycerolPhosphate | 4 | 1 |
| IM1 | (2r,4s,5s,1's)-2-phenylmethyl-4-hydroxy-5-(tert-Butoxycarbonyl)amino-6-phenylHexanoyl-n-(1'-imidazo-2-yl)-2'-methylpropanamide | 4 | 1 |
| IMO | 6-o-phosphorylInosineMonophosphate | 4 | 1 |
| INT | [[phenylmethyloxy-carbonyl]-alaninyl]-valinyl-[phenyl-1-hydroxyprop-2-yl]-amine | 4 | 1 |
| IPD | D-myo-inositol-1-phosphate | 4 | 1 |
| NLG | N-acetyl-l-glutamate | 4 | 1 |
| PEL | 2-phenyl-ethanol | 4 | 1 |
| PNS | 4'-phosphopantetheine | 4 | 1 |
| PRF | 7-deaza-7-aminomethyl-guanine | 4 | 1 |
| SOR | D-sorbitol | 4 | 1 |
| THU | Tetrahydrodeoxyuridine | 4 | 1 |
| UVW | Acetylphosphate | 4 | 1 |
| ZEN | [4-(6-chloro-naphthalene-2-sulfonyl)-piperazin-1-yl]-(3,4,5,6-tetrahydro-2h-[1,4']bipyridinyl-4-yl)-Methanone | 4 | 1 |
| 696 | 3-{5-[amino(iminio)methyl]-1h-indol-2-yl}-1,1'-Biphenyl-2-olate | 5 | 1 |
| 847 | 2-(3-{5-[amino(iminio)methyl]-1h-benzimidazol-2-yl}-5-Bromo-4-oxidophenyl)succinate | 5 | 1 |
| ABN | Benzylamine | 5 | 1 |
| BHO | BenzhydroxamicAcid | 5 | 1 |
| CR9 | 2-{5-[amino(iminio)methyl]-6-fluoro-1h-benzimidazol-2-Yl}-6-[(2-methylcyclohexyl)oxy]benzenolate | 5 | 1 |
| CZH | C2-hydroperoxy-coelenterazine | 5 | 1 |
| DCM | 2'-deoxycytidine-5'-monophosphate | 5 | 1 |
| DDF | 5,10-dideazatetrahydrofolicAcid | 5 | 1 |
| TX4 | L-threonohydroxamate4-phosphate | 5 | 1 |
| AMQ | (s)-alpha-amino-3-hydroxy-5-methyl-4-IsoxazolepropionicAcid | 6 | 1 |
| BAK | Bis(5-amidino-2-benzimidazolyl)methaneKetone | 6 | 1 |
| BPY | Biphenyl-2,3-diol | 6 | 1 |
| ESI | 4-iodobenzo[b]thiophene-2-carboxamidine | 6 | 1 |
| MK1 | N-[2(r)-hydroxy-1(s)-indanyl]-5-[(2(s)-tertiaryButylaminocarbonyl)-4(3-pyridylmethyl)piperazino]-4(s)-hydroxy-2(r)-phenylmethylpentanamide | 6 | 1 |
| AHG | 2,5-anhydroglucitol-1,6-biphosphate | 7 | 1 |
| AZM | 5-acetamido-1,3,4-thiadiazole-2-sulfonamide | 7 | 1 |
| TCL | Triclosan | 7 | 1 |
| GTS | GlutathioneSulfonicAcid | 8 | 1 |
| 3GP | Guanosine-3'-monophosphate | 9 | 1 |
| IMI | 2-iminobiotin | 9 | 1 |
| XLS | D-xylose(linearForm) | 11 | 1 |
| CB3 | 10-propargyl-5,8-dideazafolicAcid | 14 | 1 |
| 16G | N-acetyl-d-glucosamine-6-phosphate | 2 | 2 |
| 2AN | 1-anilino-8-naphthaleneSulfonate | 2 | 2 |
| 2FA | 2-(6-amino-2-fluoro-purin-9-yl)-5-hydroxymethyl-Tetrahydro-furan-3,4-diol | 2 | 2 |
| 2FD | 5-(6-amino-2-fluoro-purin-9-yl)-2-hydroxymethyl-Tetrahydro-furan-3-ol | 2 | 2 |
| 2FP | 1,6-fructoseDiphosphate(linearForm) | 2 | 2 |
| 2KT | 2-ketobutyricAcid | 2 | 2 |
| 2MD | Guanylate-o'-phosphoricAcidMono-(2-amino-5,6-Dimercapto-4-oxo-3,5,6,7,8a,9,10,10a-octahydro-4h-8-Oxa-1,3,9,10-tetraaza-anthracen-7-ylmethyl)Ester | 2 | 2 |
| 2PI | 2-amino-pentanoicAcid | 2 | 2 |
| 478 | {3-[(4-amino-benzenesulfonyl)-isobutyl-amino]-1-benzyl-2-hydroxy-propyl}-carbamicAcidTetrahydro-furan-3-ylEster | 2 | 2 |
| 4NC | 4-nitrocatechol | 2 | 2 |
| 5PA | N-[3-hydroxy-2-methyl-5-phosphonooxymethyl-pyridin-4-y-lmethyl]-1-amino-cyclopropanecarboxylicAcid | 2 | 2 |
| 8BR | 8-bromo-adenosine-5'-monophosphate | 2 | 2 |
| AGP | 2-deoxy-2-aminoGlucitol-6-phosphate | 2 | 2 |
| ALA | Alanine | 2 | 2 |
| ARA | Alpha-l-arabinose | 2 | 2 |
| ATR | 2'-monophosphoadenosine-5'-diphosphate | 2 | 2 |
| BLA | BiliverdineIxAlpha | 2 | 2 |
| CAQ | Catechol | 2 | 2 |
| CB1 | 5-(aziridin-1-yl)-2,4-dinitrobenzamide | 2 | 2 |
| CDM | 4-diphosphocytidyl-2-c-methyl-d-erythritol | 2 | 2 |
| CIO | Cilomilast | 2 | 2 |
| CMG | 6-o-cyclohexylmethylGuanine | 2 | 2 |
| CR4 | 2-{5-[amino(iminio)methyl]-1h-benzimidazol-2-yl}Benzenolate | 2 | 2 |
| CRC | CapricAcid | 2 | 2 |
| CSN | N,4-dihydroxy-n-oxo-3-(sulfooxy)benzenaminium | 2 | 2 |
| CTT | Cellotetraose | 2 | 2 |
| CXS | 3-cyclohexyl-1-propylsulfonicAcid | 2 | 2 |
| DCP | 2'-deoxycytidine-5'-triphosphate | 2 | 2 |
| DEG | ButylAlpha-d-mannopyranoside | 2 | 2 |
| DHK | 3-dehydroshikimate | 2 | 2 |
| DIF | 2-[2,6-dichlorophenyl)amino]benzeneaceticAcid | 2 | 2 |
| DIH | 3-hydroxy-4-hydroxymethyl-1-(4-oxo-4,4a,5,7a-Tetrahydro-3h-pyrrolo[3,2-d]pyrimidin-7-ylmethyl)-Pyrrolidinium | 2 | 2 |
| DMA | DimethylallylDiphosphate | 2 | 2 |
| DSO | Adamantane-1-carboxylicAcid-5-dimethylamino-Naphthalene-1-sulfonylamino-octyl-amide | 2 | 2 |
| E4P | Erythose-4-phosphate | 2 | 2 |
| FLF | 2-[[3-(trifluoromethyl)phenyl]amino]BenzoicAcid | 2 | 2 |
| GLP | Glucosamine6-phosphate | 2 | 2 |
| GOX | (2s,3s,4r,5r)-6-(hydroxyamino)-2-(hydroxymethyl)-2,3,4,5-tetrahydropyridine-3,4,5-triol | 2 | 2 |
| GPX | Guanosine5'-diphosphate2':3'-cyclicMonophosphate | 2 | 2 |
| GTG | 7-methyl-guanosine-5'-triphosphate-5'-guanosine | 2 | 2 |
| GYP | Methyl-alpha-d-glucopyranoside | 2 | 2 |
| HDF | 8-hydroxy-10-(d-ribo-2,3,4,5-tetrahydroxypentyl)-5-Deazaisoalloxazine | 2 | 2 |
| HGA | GlutamineHydroxamate | 2 | 2 |
| HHA | (2s,3s)-trans-2,3-dihydro-3-hydroxyanthranilicAcid | 2 | 2 |
| HMH | 4-amino-5-hydroxymethyl-2-methylpyrimidine | 2 | 2 |
| HPY | 4-hydroxy-3,4-dihydro-1h-pyrimidin-2-one | 2 | 2 |
| IDC | 5-hydroxymethyl-5,6,7,8-tetrahydro-imidazo[1,2-a]Pyridin-6yl-7,8-diol-glucopyranoside | 2 | 2 |
| IMN | Indomethacin | 2 | 2 |
| ING | D-[(amino)carbonyl]phenylalanine | 2 | 2 |
| ISN | Isatin | 2 | 2 |
| IVA | IsovalericAcid | 2 | 2 |
| IXM | (z)-1h,1'h-[2,3']biindolylidene-3,2'-dione-3-oxime | 2 | 2 |
| KAN | KanamycinA | 2 | 2 |
| LAU | DodecanoicAcid | 2 | 2 |
| LLA | L-2-hydroxy-3-phenyl-propionicAcid | 2 | 2 |
| LUM | Lumichrome | 2 | 2 |
| LX1 | L-xylulose5-phosphate | 2 | 2 |
| LY2 | 2-morpholin-4-yl-7-phenyl-4h-chromen-4-one | 2 | 2 |
| MAK | Alpha-ketomalonicAcid | 2 | 2 |
| MF3 | 2-amino-4-trifluoromethylsulfanyl-butyricAcid | 2 | 2 |
| MPH | (1-amino-3-methylsulfanyl-propyl)-phosphonicAcid | 2 | 2 |
| MTM | (3s,4r)-2-(4-amino-5h-pyrrolo[3,2-d]pyrimidin-7-yl)-5-[(methylsulfanyl)methyl]pyrrolidine-3,4-diol | 2 | 2 |
| NCA | 2-nitro-p-cresol | 2 | 2 |
| NCT | (s)-3-(1-methylpyrrolidin-2-yl)pyridine | 2 | 2 |
| NFG | 2,4-dinitrophenyl2-deoxy-2-fluoro-beta-d-Allopyranoside | 2 | 2 |
| NGH | N-isobutyl-n-[4-methoxyphenylsulfonyl]glycylHydroxamicAcid | 2 | 2 |
| NPR | 2-amino-7,8-dihydro-6-(1,2,3-trihydroxypropyl)-4(1h)-Pteridinone | 2 | 2 |
| OLO | Olomoucine | 2 | 2 |
| PAP | -phosphate-adenosine-5'-diphosphate | 2 | 2 |
| PCG | CyclicGuanosineMonophosphate | 2 | 2 |
| PCI | Pentachlorophenol | 2 | 2 |
| PCP | 1-alpha-pyrophosphoryl-2-alpha,3-alpha-dihydroxy-4-Beta-cyclopentane-methanol-5-phosphate | 2 | 2 |
| PIL | 3-(cyclopentyloxy)-n-(3,5-dichloropyridin-4-yl)-4-Methoxybenzamide | 2 | 2 |
| PPF | PhosphonoformicAcid | 2 | 2 |
| PRP | Alpha-phosphoribosylpyrophosphoricAcid | 2 | 2 |
| PT1 | PteroicAcid | 2 | 2 |
| RAG | Argifin | 2 | 2 |
| RIG | Argadin | 2 | 2 |
| RNS | L-rhamnose | 2 | 2 |
| ROF | 3-(cyclopropylmethoxy)-n-(3,5-dichloropyridin-4-yl)-4-(difluoromethoxy)benzamide | 2 | 2 |
| RP5 | Ribose-5-phosphate,PyranoseForm | 2 | 2 |
| SAL | 2-hydroxybenzoicAcid | 2 | 2 |
| SAP | Adenosine-5'-diphosphateMonothiophosphate | 2 | 2 |
| SLB | 5-n-acetyl-beta-d-neuraminicAcid | 2 | 2 |
| SLT | 5-(acetylamino)-3,5-dideoxynon-2-ulopyranonosyl-(2->3)-beta-d-lyxo-hexopyranosyl-(1->4)hexopyranose | 2 | 2 |
| SOG | 2-hydroxymethyl-6-octylsulfanyl-tetrahydro-pyran-3,4,5-triol | 2 | 2 |
| TBS | 4,5,6,7-tetrabromobenzotriazole | 2 | 2 |
| TDX | Thymidine-5'-diphospho-beta-d-xylose | 2 | 2 |
| TFB | Tetrahydrofuran-2-carboxylicAcid | 2 | 2 |
| TFP | 10-[3-(4-methyl-piperazin-1-yl)-propyl]-2-Trifluoromethyl-10h-phenothiazine | 2 | 2 |
| THB | Tetrahydrobiopterin | 2 | 2 |
| THR | Threonine | 2 | 2 |
| TLA | L(+)-tartaricAcid | 2 | 2 |
| TRA | AconitateIon | 2 | 2 |
| TSA | 8-hydroxy-2-oxa-bicyclo[3.3.1]non-6-ene-3,5-DicarboxylicAcid | 2 | 2 |
| UGA | Uridine-5'-diphosphate-glucuronicAcid | 2 | 2 |
| UM3 | 2'-deoxyuridine3'-monophosphate | 2 | 2 |
| UPF | Uridine-5'-diphosphate-2-deoxy-2-fluorogalactose | 2 | 2 |
| URF | 5-fluorouracil | 2 | 2 |
| VK3 | Menadione | 2 | 2 |
| ZMR | Zanamivir | 2 | 2 |
| 2AM | Adenosine-2'-monophosphate | 3 | 2 |
| ADX | Adenosine-5'-phosphosulfate | 3 | 2 |
| AMG | Alpha-methyl-d-galactoside | 3 | 2 |
| BE2 | 2-aminobenzoicAcid | 3 | 2 |
| BES | 2-(3-amino-2-hydroxy-4-phenyl-butyrylamino)-4-methyl-PentanoicAcid | 3 | 2 |
| BUA | ButanoicAcid | 3 | 2 |
| CHT | CholineIon | 3 | 2 |
| CTR | Cellotriose | 3 | 2 |
| D16 | Tomudex | 3 | 2 |
| D3T | 2',3'-dideoxy-thymidine-5'-triphosphate | 3 | 2 |
| DCZ | 2'-deoxycytidine | 3 | 2 |
| DES | Diethylstilbestrol | 3 | 2 |
| DHY | 2-(3,4-dihydroxyphenyl)aceticAcid | 3 | 2 |
| DXP | 1-deoxy-d-xylulose-5-phosphate | 3 | 2 |
| FON | FolinicAcid | 3 | 2 |
| G3H | Glyceraldehyde-3-phosphate | 3 | 2 |
| GEN | Genistein | 3 | 2 |
| IOM | (diaminomethyl-methyl-amino)-aceticAcid | 3 | 2 |
| IPL | Indole-3-propanolPhosphate | 3 | 2 |
| KEU | N-{4-[(1r)-4-[(2r,4r,5s)-2,4-diamino-6-Oxohexahydropyrimidin-5-yl]-1-(2,2,2-trifluoro-1,1-Dihydroxyethyl)butyl]benzoyl}-d-glutamicAcid | 3 | 2 |
| KIF | Kifunensine | 3 | 2 |
| LEU | Leucine | 3 | 2 |
| MFU | Alpha-l-methyl-fucose | 3 | 2 |
| MGL | O1-methyl-glucose | 3 | 2 |
| MLR | Maltotriose | 3 | 2 |
| MTA | 5'-deoxy-5'-methylthioadenosine | 3 | 2 |
| NAC | 3-acetylPyridineAdenineDinucleotide | 3 | 2 |
| NOV | Novobiocin | 3 | 2 |
| OHT | 4-hydroxytamoxifen | 3 | 2 |
| PNN | PenicillinG | 3 | 2 |
| RIT | Ritonavir | 3 | 2 |
| S3P | Shikimate-3-phosphate | 3 | 2 |
| SPD | Spermidine | 3 | 2 |
| STI | 4-(4-methyl-piperazin-1-ylmethyl)-n-[4-methyl-3-(4-Pyridin-3-yl-pyrimidin-2-ylamino)-phenyl]-benzamide | 3 | 2 |
| TAP | 7-thionicotinamide-adenine-dinucleotidePhosphate | 3 | 2 |
| TPS | ThiaminPhosphate | 3 | 2 |
| U3P | 3'-uridinemonophosphate | 3 | 2 |
| VIA | 5-{2-ethoxy-5-[(4-methylpiperazin-1-yl)sulfonyl]Phenyl}-1-methyl-3-propyl-1h,6h,7h-pyrazolo[4,3-d]Pyrimidin-7-one | 3 | 2 |
| VIB | 3-(4-amino-2-methyl-pyrimidin-5-ylmethyl)-5-(2-hydroxy-ethyl)-4-methyl-thiazol-3-ium | 3 | 2 |
| 5RP | Ribulose-5-phosphate | 4 | 2 |
| AP2 | PhosphomethylphosphonicAcidAdenosylEster | 4 | 2 |
| AS1 | Argininosuccinate | 4 | 2 |
| BAT | 4-(n-hydroxyamino)-2r-isobutyl-2s-(2-Thienylthiomethyl)succinyl-l-phenylalanine-n-Methylamide | 4 | 2 |
| BCN | Bicine | 4 | 2 |
| DMJ | 1-deoxymannojirimycin | 4 | 2 |
| FER | 3-(4-hydroxy-3-methoxyphenyl)-2-propenoicAcid | 4 | 2 |
| GBX | 2-amino-4-[1-(carboxymethyl-carbamoyl)-2-(9-hydroxy-7,8-dioxo-7,8,9,10-tetrahydro-benzo[def]chrysen-10-Ylsulfanyl)-ethylcarbamoyl]-butyricAcid | 4 | 2 |
| GLL | Glycoluril | 4 | 2 |
| IBM | 3-isobutyl-1-methylxanthine | 4 | 2 |
| M6P | Alpha-d-mannose-6-phosphate | 4 | 2 |
| NAJ | Nicotinamide-adenine-dinucleotide(acidicForm) | 4 | 2 |
| NTZ | NojirimycineTetrazole | 4 | 2 |
| PAB | 4-aminobenzoicAcid | 4 | 2 |
| QUS | (s)-2-amino-3-(3,5-dioxo-[1,2,4]oxadiazolidin-2-yl)-PropionicAcid | 4 | 2 |
| SMA | StigmatellinA | 4 | 2 |
| UFP | 5-fluoro-2'-deoxyuridine-5'-monophosphate | 4 | 2 |
| XV6 | [4r-(4alpha,5alpha,6beta,7beta)]-3,3'-[[tetrahydro-5,6-dihydroxy-2-oxo-4,7-bis(phenylmethyl)-1h-1,3-Diazepine-1,3(2h)-diyl]Bis(methylene)]bis[n-2-Thiazolylbenzamide] | 4 | 2 |
| ATG | PhosphothiophosphoricAcid-adenylateEster | 5 | 2 |
| CRS | M-cresol | 5 | 2 |
| DUT | Deoxyuridine-5'-triphosphate | 5 | 2 |
| FK5 | 8-deethyl-8-[but-3-enyl]-ascomycin | 5 | 2 |
| FMC | Formycin | 5 | 2 |
| HSM | Histamine | 5 | 2 |
| NIO | NicotinicAcid | 5 | 2 |
| RIB | Ribose | 5 | 2 |
| ROL | Rolipram | 5 | 2 |
| TYM | Tryptophanyl-5'amp | 5 | 2 |
| 130 | 2-(2-hydroxy-biphenyl)-1h-benzoimidazole-5-Carboxamidine | 6 | 2 |
| ATM | 3'-azido-3'-deoxythymidine-5'-monophosphate | 6 | 2 |
| BET | TrimethylGlycine | 6 | 2 |
| HPA | Hypoxanthine | 6 | 2 |
| MCN | PterinCytosineDinucleotide | 6 | 2 |
| ORO | OroticAcid | 6 | 2 |
| PAL | N-(phosphonacetyl)-l-asparticAcid | 6 | 2 |
| 7HP | 7-hydroxy-pyrazolo[4,3-d]pyrimidine | 7 | 2 |
| THM | Thymidine | 8 | 2 |
| UP6 | 6-azaUridine5'-monophosphate | 8 | 2 |
| MA4 | Cyclohexyl-hexyl-beta-d-maltoside | 8 | 2 |
| NAI | 1,4-dihydronicotinamideAdenineDinucleotide | 8 | 2 |
| XYL | D-xylitol | 8 | 2 |
| OXM | OxamicAcid | 9 | 2 |
| FOL | FolicAcid | 11 | 2 |
| CAP | 2-carboxyarabinitol-1,5-diphosphate | 14 | 2 |
| 2GP | Guanosine-2'-monophosphate | 39 | 2 |
| 144 | Tris-hydroxymethyl-methyl-ammonium | 3 | 3 |
| ANL | Aniline | 3 | 3 |
| ASC | AscorbicAcid | 3 | 3 |
| BI1 | 3-{1-[3-(dimethylamino)propyl]-1h-indol-3-yl}-4-(1h-Indol-3-yl)-1h-pyrrole-2,5-dione | 3 | 3 |
| BZS | L-benzylsuccinicAcid | 3 | 3 |
| DG3 | 2'-3'-dideoxyguanosine-5'-triphosphate | 3 | 3 |
| DTD | DithianeDiol | 3 | 3 |
| FBP | Fructose-1,6-diphosphate | 3 | 3 |
| GMP | Guanosine | 3 | 3 |
| GTA | P1-7-methylguanosine-p3-adenosine-5',5'-triphosphate | 3 | 3 |
| HCS | 2-amino-4-mercapto-butyricAcid | 3 | 3 |
| INR | 2',3-dioxo-1,1',2',3-tetrahydro-2,3'-biindole-5'-SulfonicAcid | 3 | 3 |
| MED | D-methionine | 3 | 3 |
| MTE | PhosphonicAcidmono-(2-amino-5,6-dimercapto-4-oxo-3,7,8a,9,10,10a-hexahydro-4h-8-oxa-1,3,9,10-tetraaza-Anthracen-7-ylmethyl)ester | 3 | 3 |
| NAR | Naringenin | 3 | 3 |
| OAA | OxaloacetateIon | 3 | 3 |
| PGU | 4-[(3-hydroxy-2-methyl-5-phosphonooxymethyl-pyridin-4-Ylmethyl)-amino]-butyricAcid | 3 | 3 |
| SER | Serine | 3 | 3 |
| SSA | 5'-o-(n-(l-seryl)-sulfamoyl)adenosine | 3 | 3 |
| STL | Resveratrol | 3 | 3 |
| U2P | PhosphoricAcidMono-[2-(2,4-dioxo-3,4-dihydro-2h-Pyrimidin-1-yl)-4-hydroxy-5-hydroxymethyl-tetrahydro-Furan-3-yl]Ester | 3 | 3 |
| UTP | Uridine5'-triphosphate | 3 | 3 |
| VAL | Valine | 3 | 3 |
| VDN | 2-{2-ethoxy-5-[(4-ethylpiperazin-1-yl)sulfonyl]phenyl}-5-methyl-7-propylimidazo[5,1-f][1,2,4]triazin-4(1h)-One | 3 | 3 |
| XYP | Beta-d-xylopyranose | 3 | 3 |
| CIR | Citrulline | 4 | 3 |
| CRB | [1r-(1alpha,3beta,4alpha,5beta)]-5-(phosphonomethyl)-1,3,4-trihydroxycyclohexane-1-carboxylicAcid | 4 | 3 |
| DGP | 2'-deoxyguanosine-5'-monophosphate | 4 | 3 |
| GNT | (-)-galanthamine | 4 | 3 |
| MCO | 1-(3-mercapto-2-methyl-propionyl)-pyrrolidine-2-CarboxylicAcid | 4 | 3 |
| NOJ | 1-deoxynojirimycin | 4 | 3 |
| PIN | Piperazine-n,n'-bis(2-ethanesulfonicAcid) | 4 | 3 |
| R5P | Ribose-5-phosphate | 4 | 3 |
| XMP | Xanthosine-5'-monophosphate | 4 | 3 |
| 017 | (3r,3as,6ar)-hexahydrofuro[2,3-b]furan-3-yl(1s,2r)-3-[[(4-aminophenyl)sulfonyl](isobutyl)amino]-1-benzyl-2-Hydroxypropylcarbamate | 5 | 3 |
| BIO | Biopterin | 5 | 3 |
| CDP | Cytidine-5'-diphosphate | 5 | 3 |
| DAU | 2'deoxy-thymidine-5'-diphospho-alpha-d-glucose | 5 | 3 |
| ETF | Trifluoroethanol | 5 | 3 |
| IMH | 1,4-dideoxy-4-aza-1-(s)-(9-deazahypoxanthin-9-yl)-d-Ribitol | 5 | 3 |
| LAC | LacticAcid | 5 | 3 |
| MTT | Maltotetraose | 5 | 3 |
| NMN | Beta-nicotinamideRiboseMonophosphate | 5 | 3 |
| UMA | Uridine-5'-diphosphate-n-acetylmuramoyl-l-alanine | 5 | 3 |
| DND | NicotinicAcidAdenineDinucleotide | 6 | 3 |
| PYZ | 4-iodopyrazole | 6 | 3 |
| RIP | Ribose(pyranoseForm) | 6 | 3 |
| SCP | [3-hydroxy-2-methyl-5-phosphonooxymethyl-pyridin-4-Ylmethyl]-n,o-cycloserylamide | 6 | 3 |
| 2PG | 2-phosphoglycericAcid | 7 | 3 |
| 4IP | Inositol-(1,3,4,5)-tetrakisphosphate | 7 | 3 |
| HCI | HydrocinnamicAcid | 7 | 3 |
| HIS | Histidine | 7 | 3 |
| KAI | 3-carboxymethyl-4-isopropyl-pyrrolidine-2-carboxylicAcid | 7 | 3 |
| GTO | PhosphomethylphosphonicAcid-guanylateEster | 9 | 3 |
| GPS | (9s,10s)-9-(s-glutathionyl)-10-hydroxy-9,10-Dihydrophenanthrene | 10 | 3 |
| PMP | 4'-deoxy-4'-aminopyridoxal-5'-phosphate | 10 | 3 |
| FUM | FumaricAcid | 12 | 3 |
| PGH | PhosphoglycolohydroxamicAcid | 13 | 3 |
| ICT | IsocitricAcid | 15 | 3 |
| DAN | 2-deoxy-2,3-dehydro-n-acetyl-neuraminicAcid | 16 | 3 |
| GTX | S-hexylglutathione | 16 | 3 |
| PGA | 2-phosphoglycolicAcid | 19 | 3 |
| CAM | Camphor | 22 | 3 |
| BTN | Biotin | 28 | 3 |
| DCT | 2',3'-dideoxycytidine5'-triphosphate | 4 | 4 |
| GUA | GlutaricAcid | 4 | 4 |
| KIV | 3-methyl-2-oxobutanoicAcid | 4 | 4 |
| NOS | Inosine | 4 | 4 |
| PHE | Phenylalanine | 4 | 4 |
| PP3 | Alanyl-pyridoxal-5'-phosphate | 4 | 4 |
| RBF | Riboflavine | 4 | 4 |
| TRE | Trehalose | 4 | 4 |
| 6PG | 6-phosphogluconicAcid | 5 | 4 |
| CBS | Di(n-acetyl-d-glucosamine) | 5 | 4 |
| DHB | 3,4-dihydroxybenzoicAcid | 5 | 4 |
| IND | Indole | 5 | 4 |
| MAN | Alpha-d-mannose | 5 | 4 |
| NHE | 2-[n-cyclohexylamino]ethaneSulfonicAcid | 5 | 4 |
| PA5 | 5-phosphoarabinonicAcid | 5 | 4 |
| XYS | Xylopyranose | 5 | 4 |
| LYS | Lysine | 6 | 4 |
| NGA | N-acetyl-d-galactosamine | 6 | 4 |
| HHP | 6-hydroxymethyl-7,8-dihydropterin | 7 | 4 |
| I3P | D-myo-inositol-1,4,5-triphosphate | 7 | 4 |
| MPO | 3[n-morpholino]propaneSulfonicAcid | 7 | 4 |
| ACR | Acarbose | 8 | 4 |
| IMP | InosinicAcid | 8 | 4 |
| SIA | O-sialicAcid | 8 | 4 |
| URA | Uracil | 14 | 4 |
| UMP | 2'-deoxyuridine5'-monophosphate | 18 | 4 |
| AKG | 2-oxyglutaricAcid | 19 | 4 |
| MGD | 2-amino-5,6-dimercapto-7-methyl-3,7,8a,9-tetrahydro-8-Oxa-1,3,9,10-tetraaza-anthracen-4-oneGuanosineDinucleotide | 21 | 4 |
| UPG | Uridine-5'-diphosphate-glucose | 25 | 4 |
| BEN | Benzamidine | 47 | 4 |
| GDU | Galactose-uridine-5'-diphosphate | 5 | 5 |
| GLN | Glutamine | 5 | 5 |
| MLT | MalateIon | 5 | 5 |
| CBI | Cellobiose | 7 | 5 |
| G1P | Alpha-d-glucose-1-phosphate | 7 | 5 |
| POC | Phosphocholine | 7 | 5 |
| HBI | 7,8-dihydrobiopterin | 8 | 5 |
| TYR | Tyrosine | 8 | 5 |
| 3PG | 3-phosphoglycericAcid | 9 | 5 |
| MAM | 1-o-methyl-alpha-d-mannose | 9 | 5 |
| TYD | Thymidine-5'-Diphosphate | 9 | 5 |
| LAT | Lactose | 14 | 5 |
| STO | Staurosporine | 14 | 5 |
| MAE | MaleicAcid | 17 | 5 |
| PEP | Phosphoenolpyruvate | 18 | 5 |
| UD1 | Uridine-diphosphate-n-acetylglucosamine | 20 | 5 |
| MTX | Methotrexate | 24 | 5 |
| PHB | P-hydroxybenzoicAcid | 24 | 5 |
| PTP | Thymidine-3',5'-diphosphate | 28 | 5 |
| ASP | AsparticAcid | 6 | 6 |
| GUN | Guanine | 6 | 6 |
| HAE | AcetohydroxamicAcid | 6 | 6 |
| FUC | Fucose | 7 | 6 |
| G6P | Alpha-d-glucose-6-phosphate | 7 | 6 |
| ACP | PhosphomethylphosphonicAcidAdenylateEster | 8 | 6 |
| GSP | 5'-guanosine-diphosphate-monothiophosphate | 11 | 6 |
| C5P | Cytidine-5'-monophosphate | 14 | 6 |
| APC | DiphosphomethylphosphonicAcidAdenosylEster | 21 | 6 |
| TMP | Thymidine-5'-phosphate | 21 | 6 |
| F6P | Fructose-6-phosphate | 34 | 6 |
| SAH | S-adenosyl-l-homocysteine | 59 | 6 |
| PQQ | PyrroloquinolineQuinone | 8 | 7 |
| TTP | Thymidine-5'-triphosphate | 8 | 7 |
| ORN | Ornithine | 9 | 7 |
| APR | Adenosine-5-diphosphoribose | 15 | 7 |
| CTP | Cytidine-5'-triphosphate | 17 | 7 |
| A3P | Adenosine-3'-5'-diphosphate | 18 | 7 |
| TRP | Tryptophan | 19 | 7 |
| UDP | Uridine-5'-diphosphate | 35 | 7 |
| GNP | PhosphoaminophosphonicAcid-guanylateEster | 48 | 7 |
| SIN | SuccinicAcid | 10 | 8 |
| CMP | Adenosine-3',5'-cyclic-monophosphate | 13 | 8 |
| U5P | Uridine-5'-monophosphate | 15 | 8 |
| MAL | Maltose | 17 | 8 |
| PYR | PyruvicAcid | 17 | 8 |
| GSH | Gluthathione | 46 | 8 |
| MET | Methionine | 11 | 9 |
| MLA | MalonicAcid | 12 | 9 |
| IPH | Phenol | 15 | 9 |
| SUC | Sucrose | 34 | 9 |
| GLY | Glycine | 12 | 10 |
| PLP | Pyridoxal-5'-phosphate | 17 | 10 |
| TDP | ThiaminDiphosphate | 36 | 10 |
| ARG | Arginine | 17 | 11 |
| ADE | Adenine | 19 | 11 |
| ADN | Adenosine | 20 | 11 |
| 5GP | Guanosine-5'-monophosphate | 19 | 12 |
| NAG | N-acetyl-d-glucosamine | 22 | 12 |
| GAL | D-galactose | 26 | 12 |
| SAM | S-adenosylmethionine | 41 | 13 |
| GLC | Glucose | 47 | 13 |
| GLU | GlutamicAcid | 17 | 14 |
| COA | CoenzymeA | 29 | 14 |
| GTP | Guanosine-5'-triphosphate | 26 | 15 |
| AMP | AdenosineMonophosphate | 29 | 15 |
| NAP | NadpNicotinamide-adenine-dinucleotidePhosphate | 73 | 16 |
| FMN | FlavinMononucleotide | 122 | 16 |
| NDP | NadphDihydro-nicotinamide-adenine-dinucleotidePhosphate | 44 | 18 |
| ANP | PhosphoaminophosphonicAcid-adenylateEster | 59 | 19 |
| FAD | Flavin-adenineDinucleotide | 152 | 21 |
| NAD | Nicotinamide-adenine-dinucleotide | 149 | 27 |
| GDP | Guanosine-5'-diphosphate | 86 | 29 |
| ADP | Adenosine-5'-diphosphate | 137 | 31 |
| ATP | Adenosine-5'-triphosphate | 97 | 35 |

^a^ To remove the redundancy in domain dataset, only one representative was chosen from each kind of protein to count the ligand-binding domains.
