# Supplementary material for: Distribution patterns of small-molecule ligands in the protein universe and implications for origin of life and drug discovery
Source: Genome Biol. 2007 Aug 29;8(8):R176. doi: 10.1186/gb-2007-8-8-r176 (PMC2375006; doi:10.1186/gb-2007-8-8-r176)
Supplement: Additional data file 2 — Power-law behaviors of metabolism-relevant ligands. [file gb-2007-8-8-r176-S2.doc]

**Additional data file 2**

**Fig. 1 (a)**

**Fig. 1 (b)**

##### Figure 1. Power-law behaviors of metabolism-relevant ligands. The number of ligands (*N*) decays with the increase of number (*L*) of domains (a) and folds (b) that bind the ligand and follows the equation *N* = *aL^-b^*. The figure illustrates that a few ligands cover tens of protein domains or folds, while most of ligands are owned by only one domain or fold.
