# Supplementary material for: Distribution patterns of small-molecule ligands in the protein universe and implications for origin of life and drug discovery
Source: Genome Biol. 2007 Aug 29;8(8):R176. doi: 10.1186/gb-2007-8-8-r176 (PMC2375006; doi:10.1186/gb-2007-8-8-r176)
Supplement: Additional data file 3 — Building blocks and ownerships of metabolism-relevant ligands. [file gb-2007-8-8-r176-S3.doc]

**Additional data file 3**

Building blocks and ownerships of metabolism-relevant ligands.

| **Ligands** | **Full name** | **Building block** | Ownership | | **Occurrence in fold universe** |
| --- | --- | --- | --- | --- | --- |
| ATP | Adenosine-5'-triphosphate | Adenosine | *E. coli* | Yeast | 35 |
| ADP | Adenosine-5'-diphosphate | Adenosine | *E. coli* | Yeast | 31 |
| GDP | Guanosine-5'-diphosphate | Guanosine | *E. coli* | Yeast | 29 |
| NAD | Nicotinamide-adenine-dinucleotide | Adenine | *E. coli* | Yeast | 27 |
| FAD | Flavin-adenineDinucleotide | Adenine | *E. coli* | Yeast | 21 |
| NDP | NadphDihydro-nicotinamide-adenine-dinucleotidePhosphate | Adenine | *E. coli* | Yeast | 18 |
| NAP | NadpNicotinamide-adenine-dinucleotidePhosphate | Adenine | *E. coli* | Yeast | 16 |
| FMN | FlavinMononucleotide | - | *E. coli* | Yeast | 16 |
| AMP | AdenosineMonophosphate | Adenosine | *E. coli* | Yeast | 15 |
| COA | CoenzymeA | Adenosine | *E. coli* | Yeast | 14 |
| SAM | S-adenosylmethionine | Adenosine & methionine | *E. coli* | Yeast | 13 |
| GLC | Glucose | Glucose | *E. coli* | Yeast | 13 |
| NAG | N-acetyl-d-glucosamine | Glucose | *E. coli* | Yeast | 12 |
| GAL | D-galactose | Galactose | *E. coli* | Yeast | 12 |
| ADE | Adenine | Adenine | *E. coli* | Yeast | 11 |
| ADN | Adenosine | Adenosine | *E. coli* | Yeast | 11 |
| GLY | Glycine | Glycine | *E. coli* | Yeast | 10 |
| PLP | Pyridoxal-5'-phosphate | - | *E. coli* | Yeast | 10 |
| TDP | ThiaminDiphosphate | Thiamin | *E. coli* | Yeast | 10 |
| IPH | Phenol | - | *E. coli* | Yeast | 9 |
| SUC | Sucrose | Sucrose | *E. coli* | Yeast | 9 |
| CMP | Adenosine-3',5'-cyclic-monophosphate | Adenosine | *E. coli* | Yeast | 8 |
| MAL | Maltose | Maltose | *E. coli* | Yeast | 8 |
| TRP | Tryptophan | Tryptophan | *E. coli* | Mouse | 7 |
| UDP | Uridine-5'-diphosphate | Uridine | *E. coli* | Yeast | 7 |
| PQQ | PyrroloquinolineQuinone | - | *E. coli* | Yeast | 7 |
| GUN | Guanine | Guanine | *E. coli* | Yeast | 6 |
| FUC | Fucose | Fucose | *E. coli* | *Homo sapiens* | 6 |
| MLT | MalateIon | - | *E. coli* | Yeast | 5 |
| CBI | Cellobiose | Cellobiose | *E. coli* | Yeast | 5 |
| LAT | Lactose | Lactose | *E. coli* | Yeast | 5 |
| PEP | Phosphoenolpyruvate | - | *E. coli* | Yeast | 5 |
| MTX | Methotrexate | - | *E. coli* | Yeast | 5 |
| STO | Staurosporine | - | *E. coli* | Yeast | 5 |
| NOS | Inosine | Inosine | *E. coli* | Yeast | 4 |
| RBF | Riboflavine | - | *E. coli* | Yeast | 4 |
| TRE | Trehalose | Trehalose | *E. coli* | Yeast | 4 |
| IND | Indole | - | *E. coli* | Yeast | 4 |
| NGA | N-acetyl-d-galactosamine | Galactose | *E. coli* | *Oryza sativa* | 4 |
| URA | Uracil | Uracil | *E. coli* | Yeast | 4 |
| FBP | Fructose-1,6-diphosphate | Fructose | *E. coli* | *Homo sapiens* | 3 |
| GMP | Guanosine | Guanosine | *E. coli* | Yeast | 3 |
| MED | D-methionine | Methionine | *E. coli* | Yeast | 3 |
| OAA | OxaloacetateIon | - | *E. coli* | Yeast | 3 |
| CIR | Citrulline | Citrulline | *E. coli* | Yeast | 3 |
| MTT | Maltotetraose | - | *E. coli* | Yeast | 3 |
| BTN | Biotin | - | *E. coli* | Yeast | 3 |
| ANL | Aniline | - | *E. coli* | Yeast | 3 |
| NAR | Naringenin | - | - | *Homo sapiens* | 3 |
| STL | Resveratrol | - | - | *Homo sapiens* | 3 |
| NOJ | 1-deoxynojirimycin | - | - | Yeast | 3 |
| 16G | N-acetyl-d-glucosamine-6-phosphate | Glucose | *E. coli* | Yeast | 2 |
| CAQ | Catechol | - | *E. coli* | Yeast | 2 |
| LX1 | L-xylulose5-phosphate | Xylulose | *E. coli* | Yeast | 2 |
| RNS | L-rhamnose | Rhamnose | *E. coli* | *Arabidopsis thaliana* | 2 |
| PNN | PenicillinG | - | *E. coli* | *Arabidopsis thaliana* | 2 |
| S3P | Shikimate-3-phosphate | - | *E. coli* | Yeast | 2 |
| SPD | Spermidine | - | *E. coli* | *Homo sapiens* | 2 |
| TPS | Thiamin Phosphate | Thiamin | *E. coli* | Yeast | 2 |
| FMC | Formycin | - | *E. coli* | *Homo sapiens* | 2 |
| HPA | Hypoxanthine | Xanthine | *E. coli* | *Homo sapiens* | 2 |
| FOL | Folic Acid | - | *E. coli* | Yeast | 2 |
| ISN | Isatin | - | - | *Homo sapiens* | 2 |
| LUM | Lumichrome | - | - | *Homo sapiens* | 2 |
| PCI | Pentachlorophenol | - | - | *Homo sapiens* | 2 |
| VK3 | Menadione | - | - | *Homo sapiens* | 2 |
| CTR | Cellotriose | - | - | *Homo sapiens* | 2 |
| GEN | Genistein | - | - | *Homo sapiens* | 2 |
| MLR | Maltotriose | Maltotriose | *E. coli* | Yeast | 2 |
| HSM | Histamine | Histidine | *E. coli* | Yeast | 2 |
| CDI | 2c-methyl-d-erythritol2,4-cyclodiphosphate | Erythritol | *E. coli* | *Arabidopsis thaliana* | 1 |
| HSO | Histidinol | Histidine | *E. coli* | Yeast | 1 |
| MLC | Malonyl-coenzymeA | Adenosine | *E. coli* | Yeast | 1 |
| PXP | Pyridoxine-5'-phosphate | - | *E. coli* | *Homo sapiens* | 1 |
| T3P | Thymidine-3'-phosphate | Thymidine | *E. coli* | *Homo sapiens* | 1 |
| T6P | Trehalose-6-phosphate | Trehalose | *E. coli* | Yeast | 1 |
| TDR | Thymine | Thymine | *E. coli* | Yeast | 1 |
| CLM | Chloramphenicol | - | *E. coli* | Yeast | 1 |
| NLG | N-acetyl-l-glutamate | Glutamate | *E. coli* | Yeast | 1 |
| UVW | Acetylphosphate | - | *E. coli* | *Homo sapiens* | 1 |
| 191 | PropionylCoenzymeA | Adenosine | - | *Homo sapiens* | 1 |
| ASE | N-acetylSerotonin | - | - | *Homo sapiens* | 1 |
| BMD | Butyramide | - | - | *Homo sapiens* | 1 |
| BNF | N-benzylformamide | - | - | *Homo sapiens* | 1 |
| C3P | Cytidine-3'-monophosphate | Cytidine | - | *Homo sapiens* | 1 |
| CAH | 5-exo-hydroxycamphor | - | - | *Homo sapiens* | 1 |
| CXL | Cyclohexanol | - | - | *Homo sapiens* | 1 |
| CYH | Cyclohexanone | - | *Enterobacter cloacae* | *-* | 1 |
| KDG | 2-keto-3-deoxygluconate | - | *Thermus thermophilus* | - | 1 |
| P1C | Deacetoxycephalosporin-c | - | *Streptomyces clavuligerus* | - | 1 |
| PCR | P-cresol | - | *Rhizobium loti* | *-* | 1 |
| D6G | 2-deoxy-glucose-6-phosphate | Glucose | - | Yeast | 1 |
| DA2 | Ng,ng-dimethyl-l-arginine | Arginine | - | *Homo sapiens* | 1 |
| DCY | D-cysteine | Cysteine | - | *Homo sapiens* | 1 |
| DGN | D-glutamine | Glutamine | - | *Homo sapiens* | 1 |
| DLY | D-lysine | Lysine | - | *Homo sapiens* | 1 |
| DSN | D-serine | Serine | - | *Homo sapiens* | 1 |
| DTR | D-tryptophan | Tryptophan | - | *Homo sapiens* | 1 |
| DTY | D-tyrosine | Tyrosine | - | *Homo sapiens* | 1 |
| DZN | Daidzin | - | - | *Homo sapiens* | 1 |
| ETR | N-ethylRetinamide | - | - | Cow | 1 |
| FME | N-formylmethionine | Methionine | - | *Homo sapiens* | 1 |
| FPC | D-fructose-6-phosphate(openForm) | Fructose | - | *Homo sapiens* | 1 |
| FRU | Fructose | Fructose | - | *Homo sapiens* | 1 |
| ISC | Isochorismate | - | - | Oryza sativa | 1 |
| LTN | L-tryptophanamide | Tryptophan | - | *Homo sapiens* | 1 |
| MYT | Metyrapone | - | - | *Homo sapiens* | 1 |
| NPO | P-nitrophenol | - | - | *Homo sapiens* | 1 |
| OCH | Quinolin-2(1h)-One | - | - | Yeast | 1 |
| POA | Phosphonoacetaldehyde | - | - | *Leishmania major* | 1 |
| PPR | Phosphonopyruvate | - | - | Sugarcane | 1 |
| PTO | Pseudotropine | - | - | *Solanum tuberosum* | 1 |
| S6P | D-Sorbitol-6-Phosphate | Sorbitol | - | *Homo sapiens* | 1 |
| SPV | Sulfopyruvate | - | - | *Tetrahymena pyriformis* | 1 |
| TPO | Phosphothreonine | - | - | *Homo sapiens* | 1 |
| 5AD | 5'-deoxyadenosine | Adenosine | - | *Homo sapiens* | 1 |
| AG2 | Agmatine | - | - | *Homo sapiens* | 1 |
| C2P | Cytidine-2'-monophosphate | Cytidine | - | *Homo sapiens* | 1 |
| CYS | Cysteine | Cysteine | - | *Homo sapiens* | 1 |
| DAR | D-arginine | Arginine | - | *Homo sapiens* | 1 |
| PYC | Pyrrole-2-carboxylate | - | - | *Homo sapiens* | 1 |
| RCO | Resorcinol | - | - | *Homo sapiens* | 1 |
| URI | Uridine | Uridine | - | *Homo sapiens* | 1 |
| PEL | 2-phenyl-ethanol |  | - | *Homo sapiens* | 1 |
| SOR | D-sorbitol | Sorbitol | - | *Homo sapiens* | 1 |
| ABN | Benzylamine | - | - | *Homo sapiens* | 1 |
| BPY | Biphenyl-2,3-diol | - | - | Mouse | 1 |
| THM | Thymidine | Thymidine | - | *Homo sapiens* | 1 |
