# Supplementary material for: Distribution patterns of small-molecule ligands in the protein universe and implications for origin of life and drug discovery
Source: Genome Biol. 2007 Aug 29;8(8):R176. doi: 10.1186/gb-2007-8-8-r176 (PMC2375006; doi:10.1186/gb-2007-8-8-r176)
Supplement: Additional data file 4 — Power-law behaviors of folds for proteins binding ATP, ADP and NAD. [file gb-2007-8-8-r176-S4.doc]

**Additional data file 4**

**Fig. S2 (a)**

**Fig. S2 (b)**

**Fig. S2 (c)**

Figure S2. Power-law behaviors of folds for proteins binding ATP (**a**), ADP (**b**) and NAD (**c**). The number of folds (*F*) decays with the increase of fold occurrence in domain space (*D*) and follows the equation: *F* = *aD^-b^*, which allows us to deduce the most ancestral host proteins that bind the ligands.
