# Supplementary material for: Distribution patterns of small-molecule ligands in the protein universe and implications for origin of life and drug discovery
Source: Genome Biol. 2007 Aug 29;8(8):R176. doi: 10.1186/gb-2007-8-8-r176 (PMC2375006; doi:10.1186/gb-2007-8-8-r176)
Supplement: Additional data file 5 — Building block usage of bio-ligands. [file gb-2007-8-8-r176-S5.doc]

**Additional data file 5**

Figure S3. Building block usage of bio-ligands. It can be found that nucleic acid bases are most frequently used building blocks, followed by carbohydrates and amino acids.
